# Functional trade‐offs and the phylogenetic dispersion of seed traits in a biodiversity hotspot of the Mountains of Southwest China

**DOI:** 10.1002/ece3.3805

**Published:** 2018-01-25

**Authors:** Kai Chen, Kevin S. Burgess, Xiang‐Yun Yang, Ya‐Huang Luo, Lian‐Ming Gao, De‐Zhu Li

**Affiliations:** ^1^ Germplasm Bank of Wild Species Kunming Institute of Botany Chinese Academy of Sciences Kunming China; ^2^ Kunming College of Life Science University of Chinese Academy of Sciences Kunming China; ^3^ School of Resources and the Environment Baoshan University Baoshan China; ^4^ Department of Biology College of Letters and Sciences Columbus State University University System of Georgia Columbus GA USA; ^5^ Key Laboratory for Plant diversity and Biogeography of East Asia Kunming Institute of Botany Chinese Academy of Sciences Kunming China

**Keywords:** functional traits, Mountains of Southwest China, seed mass, seed number, time to germination

## Abstract

The diversity of traits associated with plant regeneration is often shaped by functional trade‐offs where plants typically do not excel at every function because resources allocated to one function cannot be allocated to another. By analyzing correlations among seed traits, empirical studies have shown that there is a trade‐off between seedling development and the occupation of new habitats, although only a small range of taxa have been tested; whether such trade‐off exists in a biodiverse and complex landscape remains unclear. Here, we amassed seed trait data of 1,119 species from a biodiversity hotspot of the Mountains of Southwest China and analyzed the relationship between seed mass and the number of seeds and between seed mass and time to germination. Our results showed that seed mass was negatively correlated with seed number but positively correlated with time to germination. The same trend was found regardless of variation in life‐form and phylogenetic conservatism. Furthermore, the relation between seed mass and other seed traits was randomly dispersed across the phylogeny at both the order and family levels. Collectively, results suggest that there is a functional trade‐off between seedling development and new habitat occupation for seed plants in this region. Larger seeds tend to produce fewer seedlings but with greater fitness compared to those produced by smaller seeds, whereas smaller seeds tend to have a larger number of seeds that germinate faster compared to large‐seeded species. Apart from genetic constraints, species that produce large seeds will succeed in sites where resource availability is low, whereas species with high colonization ability (those that produce a high number of seeds per fruit) will succeed in new niches. This study provides a mechanistic explanation for the relatively high levels of plant diversity currently found in a heterogeneous region of the Mountains of Southwest China.

## 1 INTRODUCTION

Functional traits associated with species coexistence often result in trade‐offs due to disparate allocations of limited resources (Leishman, 2001; Liu & Ma, 2015; Reich, 2014). In seed plants, this ecological strategy has been demonstrated in the leaves, stems, fruits (referring to seedling recruitment), and seeds and at the whole plant level (Chave et al., 2009; Moles, Ackerly, Webb, Tweddle, Dickie, Pitman, et al., 2005; Muller‐Landau, 2010; Muñoz, Schaefer, Böhning‐Gaese, & Schleuning, 2016; Philipson et al., 2014; Wright et al., 2010; Wright et al., 2004). Trade‐offs associated with seed traits play an important role in explaining species coexistence (Muller‐Landau, 2010). It is widely known that seed traits are closely related to seedling development and new habitat occupation (Fenner & Thompson, 2005; Kleyheeg, Treep, de Jager, Nolet, & Soons, 2017). For example, seed mass can influence the initial size of the seedling by providing provisions during the early stages of a seedling's life (Coomes & Grubb, 2003; Hu, Zhang, Wu, & Baskin, 2017; Ozinga et al., 2005; Westoby, Falster, Moles, Vesk, & Wright, 2002). Alternatively, the number of seeds a plant produces can directly contribute to its colonization ability as the number of seeds available in the surrounding landscape will determine how many seeds land in suitable patches, given that all seeds have the same dispersal effectiveness (Coomes & Grubb, 2003; Coomes, Rees, Grubb, & Turnbull, 2002; Ozinga et al., 2005). Furthermore, rapid germination can provide a positive advantage for the survival of individuals as seeds gain a longer growing season for their seedlings and reduce the impact of neighboring plants at the colonization stage (Donohue, de Casas, Burghardt, Kovach, & Willis, 2010; Dubois & Cheptou, 2012); long germination times (slow germination) is a bet hedging strategy that can increase the risk of losing a cohort of seedlings (Norden et al., 2009). Analyzing correlations between seed traits can provide novel insights into the presence of trade‐offs between those associated with seedling development and those associated with new habitat occupation (Chave et al., 2009).

Previous studies have shown that there is a negative correlation between seed mass and the number of seeds at the interspecific or intraspecific level (Giorgis, Cingolani, Gurvich, & Astegiano, 2015; Greene & Johnson, 1994; Gundel, Garibaldi, Martínez‐Ghersa, & Ghersa, 2012; Guo, Mazer, & Du, 2010; Harper, Lovell, & Moore, 1970; Jakobsson & Eriksson, 2000; Salisbury, 1943; Shipley & Dion, 1992; Stocklin, 1999; Turnbull, Rees, & Crawley, 1999; Wang, Du, Guo, & Zhao, 2009) or that there is no relationship at all (Koenig, Knops, Carmen, & Sage, 2009; Willis & Hulme, 2004). Furthermore, theoretical models have shown that large seeds germinate faster than small seeds due to postdispersal selection for predator avoidance (Blate, Peart, & Leighton, 1998; Louda, 1989; Rees, 1994; Venable & Brown, 1988). Although there is evidence that seed mass is negatively correlated with time to germination (shorter germination times) (Wu, Du, & Shi, 2013), it has also been shown that seed mass is positively correlated with time to germination (Murali, 1997; Norden et al., 2009) or there is no trade‐off at all (Hill, Edwards, & Franks, 2012; Jurado & Flores, 2005). While large‐scale trends associated with the trade‐offs of seed mass versus seed number or seed mass versus time to germination remain elusive, results suggest that the magnitude of these ecological strategies may vary depending not only on habitat but also on the extent of the evolutionary relationships among the taxa being studied.

Most studies on the trade‐offs associated with seed traits have been obtained from a small range of taxa, with relatively little data and limited statistical power (Greene & Johnson, 1994; Jakobsson & Eriksson, 2000; Shipley & Dion, 1992; Turnbull et al., 1999). In addition, a plant's life history strategy (woody vs. nonwoody) has been shown to be an important factor influencing seed mass (Moles et al., 2007; Qi et al., 2014; Zheng, Guo, & Wang, 2017), and functional traits might not be independent due to phylogeny (Ackerly, 1999). Considering an evolutionary perspective, for example, may mean that more closely related species share similar genetic constraints associated with seed trait trade‐off, which, in turn, can lead to a nonindependent evaluation of traits that are actually phylogenetically conserved (Felsenstein, 1985). Here, phylogenetic conservatisms are manifest in phylogenetically related species that resemble each other for most aspects of the traits being measured (Blomberg, Garland, & Ives, 2003) in contrast to phylogenetic dispersion, which reflects a more scattered pattern for the relation between traits and phylogeny. Fortunately, phylogenetically independent contrasts (PICs) can detect these relationships in an unbiased way; an attractive feature of PICs is that it transforms phylogenetically nonindependent trait values to statistically independent contrasts according to clade length on the phylogenetic tree (Blomberg et al., 2003). But blindly correcting for phylogeny may not be appropriate due to differences in trait conservatism among species (Agrawal, 2007). Taking these factors into account underscores how the ecological and evolutionary role of traits associated with seeds remains poorly tested across a broad suite of species in contrasting environmental habitats.

The Mountains of Southwest China region is a global biodiversity hotspot that harbors at least 13,000 plant species and is arguably the most botanically rich temperate region in the world (Myers, Mittermeier, Mittermeier, da Fonseca, & Kent, 2000). Due to its complicated geological history and dramatic variations in local climate and topography, more than 29% of species are endemic (Li & Li, 1993; Sun, 2002; Wu, 1988), and this substantial component of the world remains largely understudied. This relatively high level of plant diversity and endemism provides an ideal opportunity to study the functional trade‐offs of seed traits at a regional scale where most ecological studies to date have focused on local processes that determine plant distribution and diversity. In such a hyperdiverse environment, we predict that small‐seeded species will produce more seeds and germinate faster than large‐seeded species. In this study, we examined three seed traits (seed mass, seed number, and time to germination) for 1,119 species of seed plants collected in the Mountains of Southwest China. We aimed to address the following questions: (1) What is the relation between seed mass and seed number? (2) Does seed mass influence time to germination? (3) Do relationships among seed traits vary with life‐form (woody vs. nonwoody)? (4) Are seed traits phylogenetically conserved?

## 2 MATERIALS AND METHODS

### 2.1 Study site

**Figure 1:**
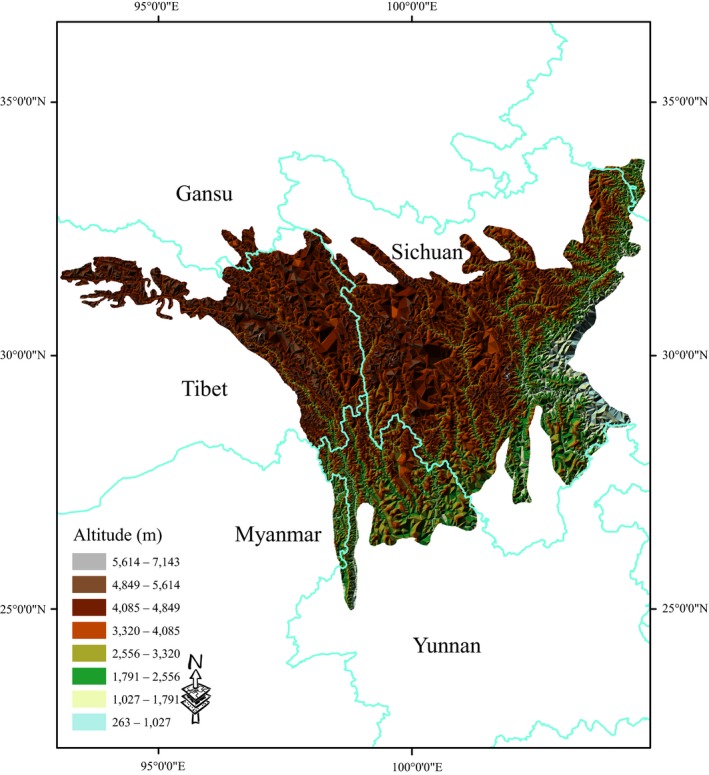
Range of plant collections from the Mountains of Southwest China that were included in our study

The Mountains of Southwest China region covers an area of approximately 262,400 km^2^, stretching from 25°0′ to 33°30′ N and from 92°30′ to 104°30′ E (Zachosl & Habel, 2011) (Figure 1). This area includes numerous mountain ranges and river systems that are oriented in a north–south direction, perpendicular to the main Himalayan chain (Zhang et al., 2014). The combined effects of geography, topography, and climate have resulted in a wide variety of vegetation types (including broad‐leaved and coniferous forests, bamboo groves, scrub communities, savanna, meadow, prairie, freshwater wetlands, and alpine scrub and scree communities) and high species richness (Zachosl & Habel, 2011).

### 2.2 Seed traits

We amassed seed trait data for 1,119 species of seed plants collected from the Mountains of Southwest China, representing 30 orders, 93 families, and 380 genera. Of these 1,119 species, 393 are woody species and the remaining 726 are nonwoody species. Our dataset represents approximately 15% of all seed plants, 30% of the genera, and around 40% of the families that occur in this region (Li & Li, 1993). Data for seed mass (based on the weight of 1,000 seeds per species) and time to germination were obtained from the Germplasm Bank of Wild Species (GBOWS). Mature seeds were dried in a drying room where the relative humidity and temperature were maintained at 15% and 15°C, respectively. Moisture was drawn out of the seeds until water content was the same as that in the air. After drying, seed mass was measured to the nearest 0.1 mg. Germination tests were conducted in incubators with a 12‐hr daily photoperiod, and each species was sown on a 1% agar medium. Temperature conditions were not uniform, as different species have different temperature range to germinate. The details of temperature conditions were listed in Table S1. All seeds that were used to test germination were stored for a year with the relative humidity at 15% and temperature at −20°C. Time to germination was calculated as TG = ∑(*D*i*Ni)/∑Ni), where Ni is the number of seeds that germinate on day Di (Saxena, Singh, & Joshi, 1996; Yu, Baskin, Baskin, Tang, & Cao, 2008). In this study, seed number was calculated as the number of seeds per fruit. We used this response variable because (1) it is very difficult to count all seeds of each species when the sample size is large; (2) previous studies showed that number of seeds per fruit differs significantly among species (Giorgis et al., 2015; Guo et al., 2010; Stocklin, 1999) and it is positively correlated with the number of fruits per plant (Kelly, 1984), a possible indicator of fecundity; and 3) empirical evidence has shown that the number of seeds per individual was more affected by plant size than seeds per fruit (which means the latter is likely a relatively stable index by comparison) (Guo et al., 2010). Most of these data were derived from online *Flora Reipublicae Popularis Sinicae*: In all cases, the maximum reported number of seeds per fruit was the value that was used for seed number in our analyses (Table S2). If *Flora Reipublicae Popularis Sinicae* did not have the record of seeds per fruit but had ovule number, ovule number was used (Table S2). Web links of these records are provided in Table S2. For 71 records, data were obtained from GBOWS (Table S2). They randomly selected five complete and ripe fruits and counted seed number of each fruit. Then, maximum value was recorded. If the seeds were too small to count, all seeds of each fruit were put on a 10 cm × 10 cm white paper and a photograph was taken (by Canon EOS‐70D, 20.20 MP). At last, ImageJ (http://imagej.net/Welcome) was used to count the seed number of each photograph.

### 2.3 Data analysis

#### 2.3.1 Phylogenetic tree construction

We built phylogenetic trees, resolved to the genus level, using the “TPL” function in “plantlist” package (Zhang, 2017) of R3.2.4 for Windows (R Core Team, 2016) and the supertree of Phylomatic (http://phylodiversity.net) based on the Angiosperm Phylogeny Group III. Branch lengths were assigned using the Bladj function of the Phylocom software, which assigned nodal ages down to the family level based on Wikström, Savolainen, and Chase (2001). Phylogenetic trees were generated at order, family, and species levels for all species as well as nonwoody and woody species separately. The “multi2d” function in “ape” package was then used to randomly resolve polytomies (Swenson, 2014).

#### 2.3.2 Seed trait analysis

We performed a series of Student's *t* tests to evaluate overall differences in each of the three seed traits among the two life history strategies (woody vs. nonwoody) represented in our dataset. Because closely related species tend to have similar traits and interspecific analyses can be biased by phylogenetic conservatism (Felsenstein, 1985; Lynch, 1991), we used Blomberg's *K* as an index of the phylogenetic conservatism. This metric was calculated using the “phylosig” function in the R package “phytools” (Swenson, 2014). Pearson correlation was performed to determine the relation between seed mass and seed number, as well as between seed mass and time to germination, using the “cor.test” function. We also analyzed our dataset using PIC correlations calculated from the three phylogenetic trees (Swenson, 2014). To meet the assumptions of normality for the Student's *t* test and the Pearson correlation, seed mass was log_10_‐transformed and seed number and time to germination were both square‐rooted before analysis.

We also wanted to test the phylogenetic dispersion of the relation between seed traits. Here, we scored each taxon as having one of three traits for the relation between seed mass and seed number: (1) a significant negative relation, (2) a significant positive relation, or (3) no relation. We also scored each taxon for the relation between seed mass and time to germination according to the same three traits. We quantified the parsimony Sankoff score for the three categorical traits arrayed on the phylogeny (Maddison & Slatkin, 1991) using “phylo.signal.disc” function (Montesinos‐Navarro, Segarra‐Moragues, Valiente‐Banuet, & Verdu, 2012). Traits were then mapped onto order‐ and family‐level phylogenetic trees to visually depict the results from the analysis. All statistical analyses were conducted with R3.2.4 for Windows (R Core Team, 2016).

## 3 RESULTS

**Figure 2:**
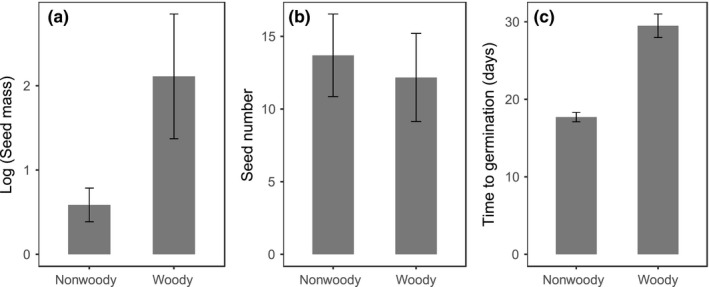
Variation in (a) seed mass, (b) seed number, and (c) time to germination for nonwoody species (726) and woody species (393) collected from our study sites in the Mountains of Southwest China

Across all 1,119 species, seed mass (based on the weight of 1,000 seeds per species) varied from 2.5 × 10^−2^ to 2.12 × 10^3 ^g (mean = 18.94 g), and on average, there were 13 seeds per fruit (seed number varied from 1 to 1,330 seeds). Time to germination ranged from 0.63 to 274.33 days, with most seeds germinating in ~21 days (Tables S3 and S4). The average seed mass of woody species was greater than that for nonwoody species (*t* = 15.42, *p *<* *.001; Figure 2a), but the mean number of seeds per fruit was not significantly different between these two life history strategies (*t* = 1.03, *p = *.3; Figure 2b). Seeds of woody species, on average, required more time to germinate than those of nonwoody species (*t* = 27.62, *p *<* *.001; Figure 2c).

We next considered phylogenetic conservatism among the three seed traits (Table 1). Across all species, the phylogenetic signals for all three seed traits were significant. The Blomberg *K*‐value was highest for the seed number and lowest for time to germination; seed mass had an intermediate value. At the order and family levels, the phylogenetic signals of all three seed traits were not significant. When nonwoody and woody species were analyzed separately, the same trends were found as that for all species.

**Table 1:** Phylogenetic signals (*K*) of seed mass, seed number, and time to germination for order, family, and species levels in the Mountains of Southwest China. *K = *1 indicates that the observed trait distribution matches the model of Brownian motion for trait evolution across the phylogenetic tree; *K *<* *1 indicates that the trait shows greater convergence than expected under the Brownian model of evolution; *p* < .05 means that the trait is more conserved compared to a random association with the phylogeny (Blomberg et al., 2003)

Trait	Order		Family		All species		Nonwoody		Woody	
	*K*	*p*	*K*	*p*	*K*	*p*	*K*	*p*	*K*	*p*
Seed mass (SM)	0.66	.839	0.38	.816	0.37	<.001	0.46	.002	0.38	.004
Seed number per fruit (SN)	0.80	.290	0.51	.457	0.48	<.001	0.53	.004	0.49	.007
Time to germination (TG)	0.71	.713	0.52	.451	0.25	<.001	0.24	.011	0.26	.003

**Figure 3:**
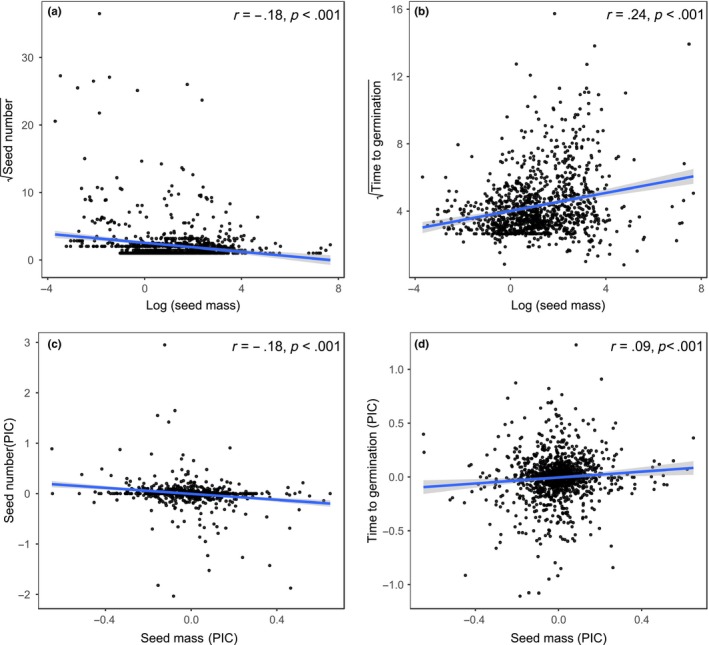
Ordinary Pearson correlations between seed mass and seed number (a) as well as between seed mass and time to germination (b) for 1,119 seed plants collected from the Mountains of Southwest China. (c) and (d) represent Ordinary Pearson correlations with phylogenetically independent contrasts for the same response variables. The gray areas represent 95% confidence intervals of models

**Figure 4:**
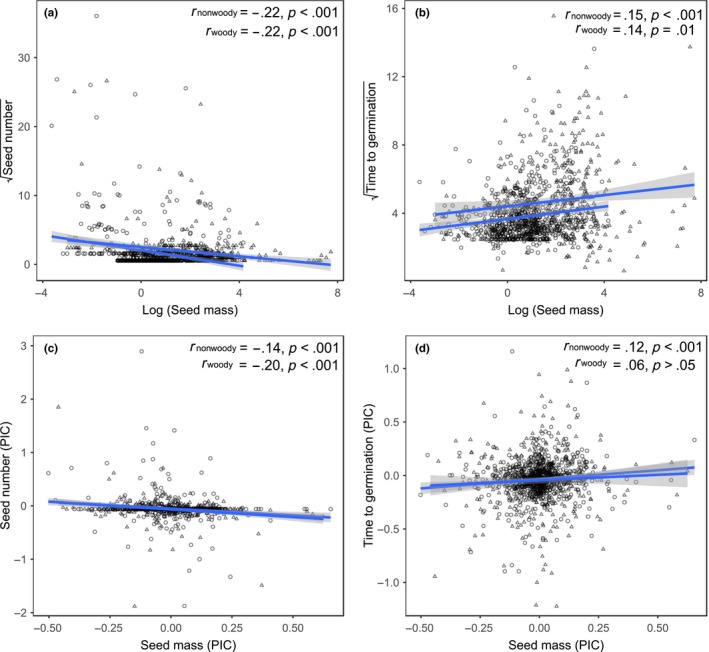
Ordinary Pearson correlations between seed mass and seed number (a) as well as between seed mass and time to germination (b) for 726 nonwoody (circles) and 393 woody species (triangles) from the Mountains of Southwest China. (c) and (d) represent ordinary Pearson correlations with phylogenetically independent contrasts for the same response variables and sources of variation. The gray areas represent 95% confidence intervals of models

Across all species, seed mass was negatively correlated with seed number (*r* = −.18 *p *<* *.001; Figures 3a and S1a) and positively correlated with the time to germination (*r* = .24, *p *<* *.001; Figures 3b and S1b). After correcting for phylogenetic effects (PICs), there was still a negative relation between seed mass and seed number across all species (*r* = −.18, *p *<* *.001; Figures 3c and S1c) as well as a positive relation between seed mass and time to germination (*r* = .09, *p *<* *.05; Figures 3d and S1d). The same trends were found for nonwoody (seed mass *r*_nonwoody_ = −.22, *p *<* *.001; time to germination *r*_nonwoody_ = .15, *p *<* *.001; Figures 4a,b and S2a,b) and woody species (seed mass *r*_woody_ = −.22, *p *<* *.001; time to germination *r*_woody_ = .14, *p *<* *.001; Figures 4a,b and S2a,b). Similarly, after correcting for phylogenetic effects (PICs), there was still a negative relation between seed mass and seed number for both nonwoody (*r*_nonwoody_ = −.14, *p *<* *.001) and woody (*r*_woody_ = −.20, *p *<* *.001) taxa (Figures 4c and S2c). Furthermore, a positive relation between seed mass and time to germination was also found for both nonwoody (*r*_nonwoody_ = .12, *p *<* *.001) and woody (*r*_woody_ = .06, *p *>* *.05) taxa (Figures 4d and S2d).

Ordinary Pearson correlation showed that the relation between seed mass and seed number as well as seed mass and time to germination varied across orders (Table 2). For example, when considering only orders that contain more than 30 species, seed mass was negatively correlated with seed number in the Asterales and Dipsacales (Table 2; Figure S3) but was positively correlated in the Apiales (Table 2 and Figure S3); no significant relationships were found for the Caryophyllales, Fabales, Lamiales, Ranunculales, and Rosales (Table 2; Figure S3). A positive relation between seed mass and time to germination was found in the Apiales, Dipsacales, and Lamiales, but no relation was detected in the Asterales, Caryophyllales, Fabales, Poales, Ranunculales, and Rosales (Table 2 and Figure S4). Similar results were obtained at the family level (Table S5). There was a positive relation between seed mass and seed number in the Lamiaceae, but no relation was found in the Berberidaceae, Fabaceae, Ranunculaceae, and Rosaceae (Table S5; Figure S5). A positive relation between seed mass and time to germination was detected in the Apiaceae, Asteraceae, and Rosaceae, no significant relationships were found in the Berberidaceae, Fabaceae, Lamiaceae, Poaceae, Polygonaceae, and Ranunculaceae (Table S5 and Figure S6).

**Table 2:** Ordinary Pearson correlations between seed mass and seed number as well as between seed mass and time to germination at the order level for 1,119 plant species from the Mountains of Southwest China. NA indicates insufficient data to complete the analysis

Order	Number of species	Traits			
		Seed mass by seed number		Seed mass by time to germination	
		Coefficient	*p*‐Value	Coefficient	*p*‐Value
Alismatales	5	0.50	>.05	0.01	>.05
Apiales	83	0.24	.031	0.31	.005
Asparagales	9	−0.89	.001	0.84	.005
Asterales	194	−0.62	<.001	0.07	>.05
Boraginales	11	NA	NA	0.12	>.05
Brassicales	10	−0.84	.002	0.06	>.05
Caryophyllales	66	0.04	>.05	−0.02	>.05
Celastrales	5	0.86	>.05	0.74	>.05
Cornales	6	0.66	>.05	−0.66	>.05
Dioscoreales	4	0.65	>.05	0.42	>.05
Dipsacales	31	−0.65	<.001	0.52	.003
Ericales	23	−0.65	<.001	−0.15	>.05
Fabales	58	0.19	>.05	0.04	>.05
Fagales	4	NA	NA	−0.28	>.05
Gentianales	18	−0.52	.029	0.38	>.05
Gnetales	3	NA	NA	0.99	>.05
Lamiales	114	−0.07	>.05	0.31	<.001
Liliales	25	−0.01	>.05	0.62	.001
Magnoliales	3	−0.11	>.05	−0.99	>.05
Malpighiales	13	−0.71	.007	−0.30	>.05
Malvales	11	−0.32	>.05	0.23	>.05
Pinales	7	−0.31	>.05	0.17	>.05
Poales	80	NA	NA	−0.20	>.05
Ranunculales	87	−0.03	>.05	0.09	>.05
Rosales	184	−0.12	>.05	0.18	>.05
Sapindales	28	−0.30	>.05	0.15	>.05
Saxifragales	3	−0.40	>.05	−0.79	>.05
Solanales	8	−0.23	>.05	−0.43	>.05
Vitales	9	0.40	>.05	0.45	>.05
Zingiberales	3	0.98	>.05	0.15	>.05

**Figure 5:**
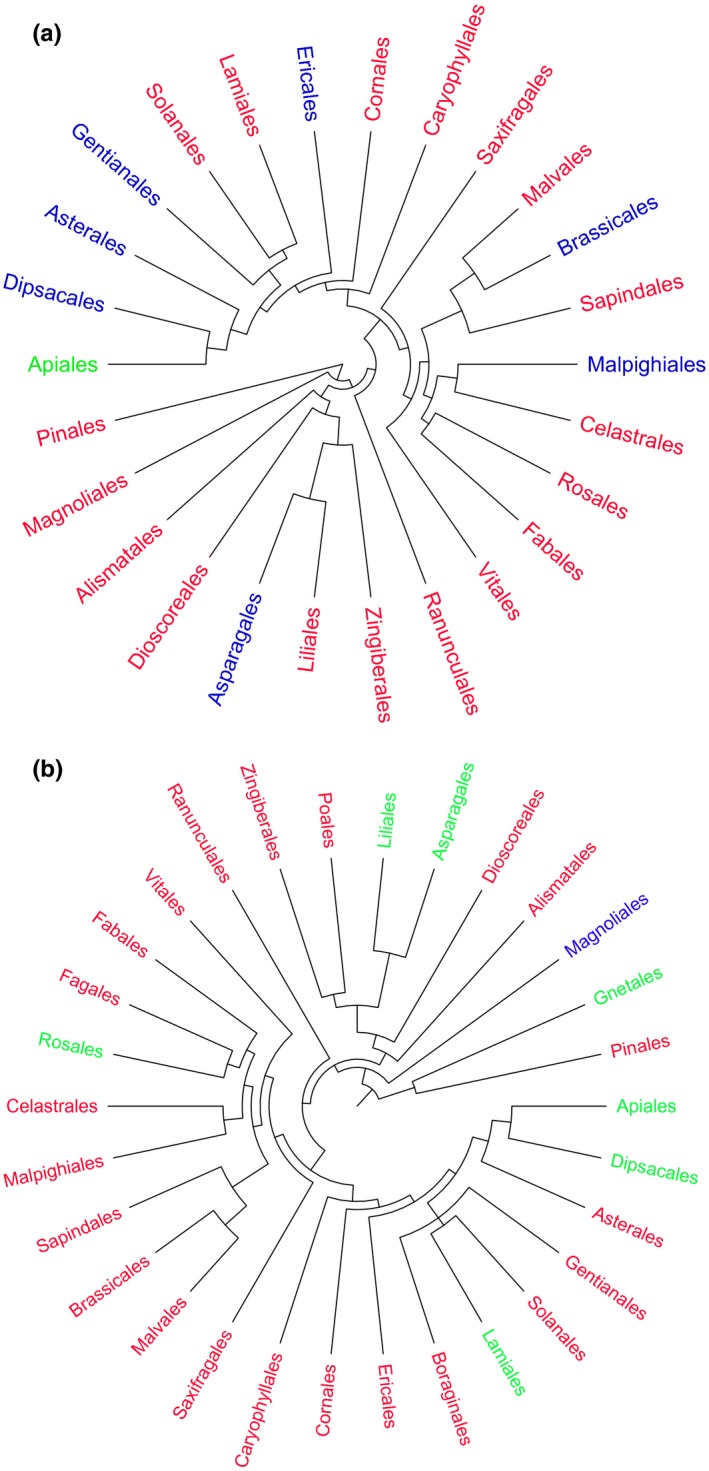
Phylogenetic tree based on APGIII at the order level for 1,119 samples collected from our study site in the Mountains of Southwest China. Tree depicts the phylogenetic dispersion of the relation between (a) seed mass and seed number as well as (b) seed mass and time to germination. The taxa highlighted in blue indicate the presence of a significant negative relation, while the taxa highlighted in green indicate the occurrence of a significant positive relation. The taxa highlighted in red did not show a significant relation among seed traits

We did not detect a phylogenetic signal for the relation between seed mass and seed number. The relation between seed mass and time to germination also showed a random distribution across the phylogeny. More specifically, for both relationships, the number of observed evolutionary transitions did not differ significantly from the mean number of evolutionary transitions under a null model at both the order and family levels (Table 3; Figure 5 and Figures S7 and S8).

**Table 3:** Phylogenetic signal for the distribution of three possible states for the relation between seed mass and seed number as well as the relation between seed mass and time to germination at the order and family levels for 1,119 samples collected in the Mountains of Southwest China. Each taxon was classified as having a negative, a positive, or a lack of relation for each source of variation. (Observed transitions = the number of observed evolutionary transitions. The expected mean null = mean number of transitions under a null model in which data were reshuffled 1,000 times across the tips of the phylogeny. The *p*‐value in each case is based on the comparison between the observed vs expected values)

	Traits					
	Seed mass by seed number			Seed mass by time to germination		
	Observed transitions	Mean null	*p*‐Value	Observed transitions	Mean null	*p*‐Value
Order	6	7	.119	6	8	.100
Family	9	9	.999	9	10	.466

## 4 DISCUSSION

### 4.1 Seed traits and phylogenetic relatedness

Overall, two of the three seed traits analyzed in this study, seed mass and time to germination, differed significantly among woody and nonwoody species in the Mountains of Southwest China. Similar trends have been found at both local (Mexican desert; Flores & Briones, 2001) and global (Díaz et al., 2016; Moles et al., 2007) scales. In the present study, differences in overall seed mass and time to germination represent different modes of resource investment (such as energy and time), confirming that global distribution patterns of woody and nonwoody species are uneven in both form and function (Díaz et al., 2016).

Phylogenetic signals (*K*) for seed mass, seed number, and time to germination reveal that seed traits are phylogenetically conserved at the species level, verifying previous findings from independent datasets (Cao et al., 2013; Kraft & Ackerly, 2010; Moles, Ackerly, Webb, Tweddle, Dickie, Pitman, et al., 2005; Norden et al., 2009; Rathcke & Lacey, 1985). This result indicates that related species share similar genetic constraints that shape their potential evolutionary responses to the environment (Rathcke & Lacey, 1985). Furthermore, because phylogenetic constraints on seed traits are regional properties of taxa, closely related species should have similar modes of energy storage, dispersal, and germination regardless of their specific geographic location, which may be a driver for the coexistence of related species (Du et al., 2015). The fact that phylogenetic constraints on seed traits may therefore be stronger than local selective pressures suggests that each species (woody or nonwoody) may employ a relatively stable seed trait strategy to assure reproductive success. It is interesting that the phylogenetic signals of seed mass, seed number, and time to germination in our study were not detected at the order and family levels (Blomberg's *K*‐values were not significant). One explanation for this is that the evolution of seed traits across the phylogeny may be not under the Brownian model of selection at the order and family levels (Blomberg et al., 2003). Collectively, our results provide a potential mechanistic explanation for seed trait variation that is shaped by evolutionary history among the plant species in the Mountains of Southwest China.

### 4.2 Relationships of seed mass with seed number and time to germination

Across all 1,119 species, we found a negative relation between seed mass and seed number. Previous studies investigating seed trait variation among species (Giorgis et al., 2015; Guo et al., 2010) or within a particular species (Agren, 1989; Vaughton & Ramsey, 1998) have found similar results at different study scales, although a lack of relation between seed mass and seed number has also been found, for example, *Impatiens glandulifera* (Willis & Hulme, 2004) and *Quercus lobata* (Koenig et al., 2009). There are two possibilities for such discrepancies among seed plants. First, some species might escape the seed mass–number trade‐off by changing the chemical or physical composition of their seeds due to selection by dispersal agents such as animals, wind or water (Lokesha, Hegde, Shaanker, & Ganeshaiah, 1992). Second, the intraspecific relationship between seed mass and seed number might be affected by stress, as species allocate more resources to reproduction under stress and thus allocate more resources to bigger or more seeds under such conditions (Koivunen, Saikkonen, Vuorisalo, & Mutikainen, 2004). In addition to these two resource constraints during seed provisioning (Vaughton & Ramsey, 1998), the trade‐off between seed mass and seed number could, to some extent, be explained by conflicts over the allocation of maternal resources within flowers prior to seed production, which often results in a trade‐off between ovule size and number (Lloyd, 1980; Westoby & Rice, 1982). Regardless of these sources of variation, overall the proportion of resources allocated to reproduction does not vary greatly among species, and plant mass and seed mass can explain most seed production variation in plant species (Shipley & Dion, 1992). That our results show the same but weaker trends compared to those of previous studies at different scales indicates that the negative relation between seed mass and seed number is likely scale‐independent (Díaz et al., 2016; Donoso, Schleuning, García, & Fründ, 2017), but the strength of this relation may be affected by the environment heterogeneity and the composition of highly diverse taxa. It is worth noting that the method to quantify seed number may be limited in our study. When we use the number of seeds per fruit as the seed number, some factors affecting species seed number, such as inflorescence architecture, fruit number, fruit size, and dispersal mode, to name a few, are not controlled for. Therefore, we suggest that field investigations that can contribute to the standardization of seed trait data should broaden our understanding of how reproductive success varies among species and across heterogeneous environments.

We found a positive relation between seed mass and time to germination. Compared to other studies, the strength of the relation between seed mass and time to germination appears to vary according the type of forest system under study (Jurado & Flores, 2005). While results similar to ours have been shown in tropical forests (Murali, 1997; Norden et al., 2009), Hill et al. (2012) studied 15 species from a seasonal tropical forest and found that a direct relation between seed mass (size) and time to germination is lacking where pregermination viability of desiccation‐sensitive seeds may not be solely determined by seed size. Such physiological constraints, however, cannot explain the positive relationship we found between seed mass and time to germination for a broad suite of plant species spanning diverse but relatively wet habitats in the Mountains of Southwest China. First, before germination, seeds need water for seed coat rupture, cell elongation, and nutrient hydrolyzation (Fenner & Thompson, 2005; Vazquez‐Yanes & Orozco‐Segovia, 1993). The longer times to germination of large seeds found in our study might be due to their higher water requirements and slower water absorption capacity due to a smaller surface area‐to‐mass ratio as well as the need to hydrolyze relatively more nutrients to support a longer germination process (Kikuzawa & Koyama, 1999). Second, species with large seeds here may have tougher physical defenses (thick endocarp or seed coat) (Blate et al., 1998; Fenner, 1983). Our results, then, are inconsistent with the general hypothesis that large seeds need to germinate quickly to avoid postdispersal predation (Janzen, 1971; Louda, 1989). The negative relationship between seed mass and postdispersal survivorship has not, however, been verified across diverse habitats, and it is possible that large seeds are protected from predation by toxic or unpalatable compounds, despite containing more nutrients (Blate et al., 1998; Finkelstein & Grubb, 2002; Janzen, 1969; Moles, Warton, & Westoby, 2003; Osunkoya, Ash, Hopkins, & Graham, 1994). Based on our results, it seems plausible that the positive relation between seed mass and time to germination has resulted from regional adaptation to wet environments, although this relation remains to be tested at the local scale.

Given that seed traits of broad phylogenetic dispersion appear to be coordinated at a regional scale in our study, it is not surprising that we found the relation between seed mass, seed number, and time to germination persisted when phylogenetic nonindependence (PIC) was accounted for in our analysis, suggesting evolutionary associations for seed traits. Our results are in line with available evidence that indicates seed traits have likely coevolved with other functional traits (Liu, Barot, El‐Kassaby, & Loeuille, 2017; Moles, Ackerly, Webb, Tweddle, Dickie, Westoby, et al., 2005). Collectively, correlated evolutionary divergence of seed mass, seed number, and time to germination might have occurred at a phylogenetic branch point deep in the past, with the trait combinations persisting within each of the descendant linages (Westoby et al., 2002). This is consistent with the fact that the mean number of evolutionary transitions under a null model (random dispersion) for the relation between seed mass and seed number as well as for the relation between seed mass and time to germination did not differ significantly from the number of observed evolutionary transitions at both the order and family levels. Thus, it appears likely that groups of seed plants under pressure from limited resources in the Mountains of Southwest China have evolved a diversity of seed trait combinations and relationships to adapt to different habitats. This point is underscored by the fact that our correlation coefficients were weaker than those found in most previous studies (Guo et al., 2010; Norden et al., 2009). The fact that we found a twofold variation in seed trait associations across the diverse habitats is certainly telling given that the correlations, although weak, were still significant (see Wang et al., 2014, 2016; Wu et al., 2013).

### 4.3 Functional traits and trade‐off strategies

Measuring the traits of a particular species and analyzing correlations among these traits is the most common approach to verify the presence of a particular functional trade‐off among species (Chave et al., 2009; Muller‐Landau, 2008). Our study provides evidence for the presence of a possible functional trade‐off between producing seedlings with high fitness and occupying new habitats among seed plants in the Mountains of Southwest China. Specifically, we found that seed mass was negatively correlated with seed number but positively correlated with time to germination. Empirical studies have shown that a short time to germination is an advantage for occupying new habitats, due, in part, to gaining a longer growing season and reducing the impact of neighboring plants (Donohue et al., 2010; Dubois & Cheptou, 2012). This trade‐off suggests that seeds found in large numbers with fast germinating ability are good at occupying new habitats, but may produce weak seedlings, whereas seeds of large size are good at producing seedlings with high fitness, but may exhibit poor ability at invading new habitats. For example, large seeds can be found in habitats with hazards, such as shade, mineral shortages, drought, and high competitiveness, but small seeds are more important in open and disturbed environments (Dwyer & Laughlin, 2017; Gross, 1984; Metcalfe, Grubb, & Turner, 1998; Westoby, Leishman, & Lord, 1996; Westoby et al., 2002). Our results support a competition–colonization trade‐off that appears to be ubiquitous among seed plants in our study region.

Although seedling establishment was not directly measured in all the habitats associated with this study, it seems likely that such trade‐off strategies coupled with spatial variation in resource availability may contribute to species coexistence (Muller‐Landau, 2010) and distribution patterns in the Mountains of Southwest China. Seed plants and communities sensitive to habitat patch dynamics, including areas of insufficient light, water, and nutrition, are able to maintain abundance by succeeding in different spatial niches (patches) (Bossuyt & Honnay, 2006; Herben & Soderstrom, 1992; Jankowska‐Blaszczuk & Daws, 2007). Species that produce seedlings with high fitness will succeed in sites where resource availability is low, whereas species with high colonization ability will succeed in new patches (Levine & Rees, 2002; Muller‐Landau, 2010). At a global scale, large‐seeded species are mainly distributed in tropical rain forests near the equator where seedlings with relatively high fitness can flourish, but small‐seeded species have wider distribution ranges due to the superior colonization ability of their seeds (Moles et al., 2007; Morin & Chuine, 2006). High spatial variation in resource availability is one of the most important characteristics of the Mountains of Southwest China. Large‐seeded species that produce seedlings with high fitness can successfully establish and reproduce in closed or shaded habitats, while small‐seeded species with high colonization ability can succeed in open environments where light is more available (Jankowska‐Blaszczuk & Daws, 2007; Muñoz et al., 2016; Westoby et al., 2002). This functional trade‐off mediates species coexistence in this region by driving different species to occupy different habitats, leading to high plant diversity.

## 5 CONCLUSIONS

Plants allocate limited resources among different functions by employing a variety of trade‐off strategies. The present study highlights potential trade‐offs between the production of seedlings with relatively fitness and the capacity to occupy new habitat over a regional flora of broad phylogenetic dispersion. This was reflected by a negative relation between seed mass and seed number as well as a positive relation between seed mass and time to germination. This functional trade‐off may provide a causal explanation for current patterns of plant abundance and diversity in biodiversity hotspots such as the Mountains of Southwest China. Although it is beyond the scope of this study to discern seed trait variation among the diversity of environments in our study region, this source of variation as well as potential evolutionary associations among taxa that occupy specific microhabitats should be considered in future studies.

## CONFLICT OF INTEREST

None declared.

## AUTHOR CONTRIBUTIONS

D.Z.L., L.M.G., and K.S.B. designed the study; K.C. and X.Y.Y. collected the data; K.C. conducted statistical analysis and generated the graphs; Y.H.L. interpreted the data; K.C., K.S.B., and L.M.G. wrote the manuscript; and K.C., K.S.B., D.Z.L., L.M.G., Y.H.L., and X.Y.Y. revised the manuscript. All authors reviewed and approved the final manuscript.
