# Supplementary figures and images for: Functional trade‐offs and the phylogenetic dispersion of seed traits in a biodiversity hotspot of the Mountains of Southwest China
Source: Ecol Evol. 2018 Jan 25;8(4):2218–30. doi: 10.1002/ece3.3805 (PMC5817125; doi:10.1002/ece3.3805)

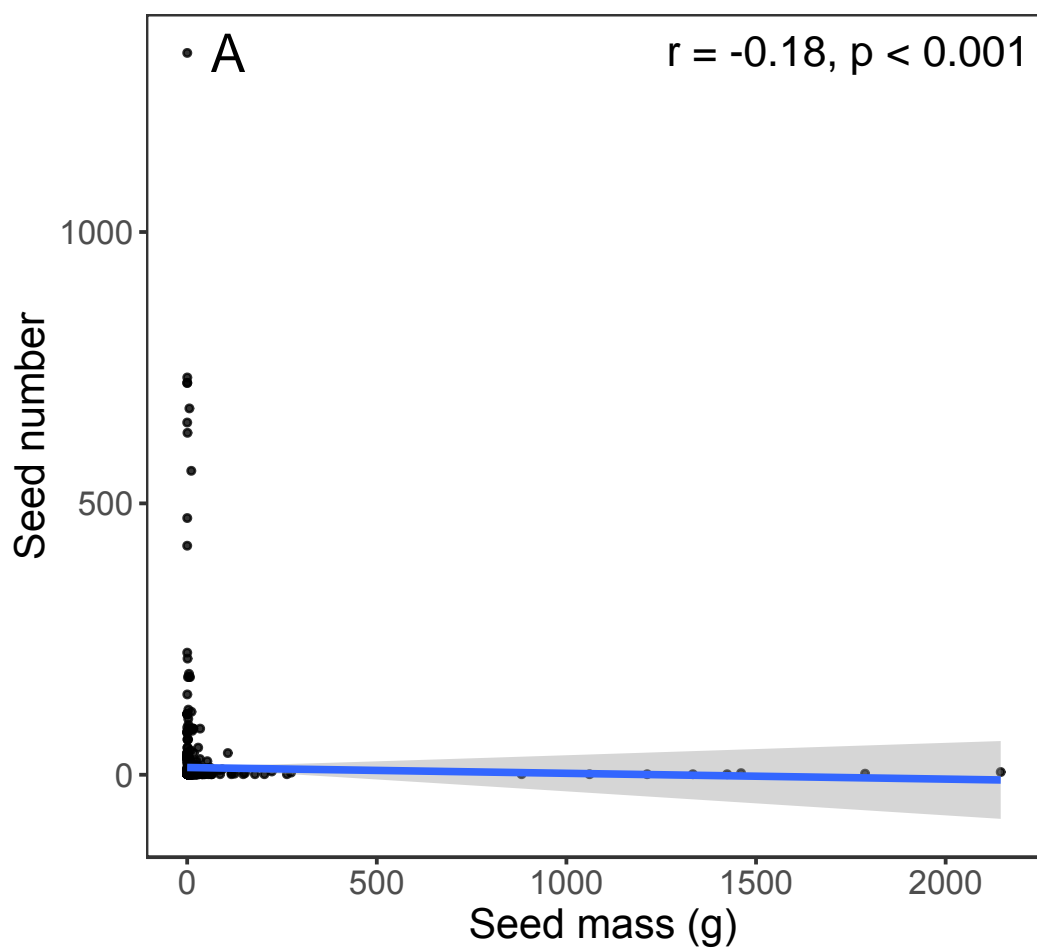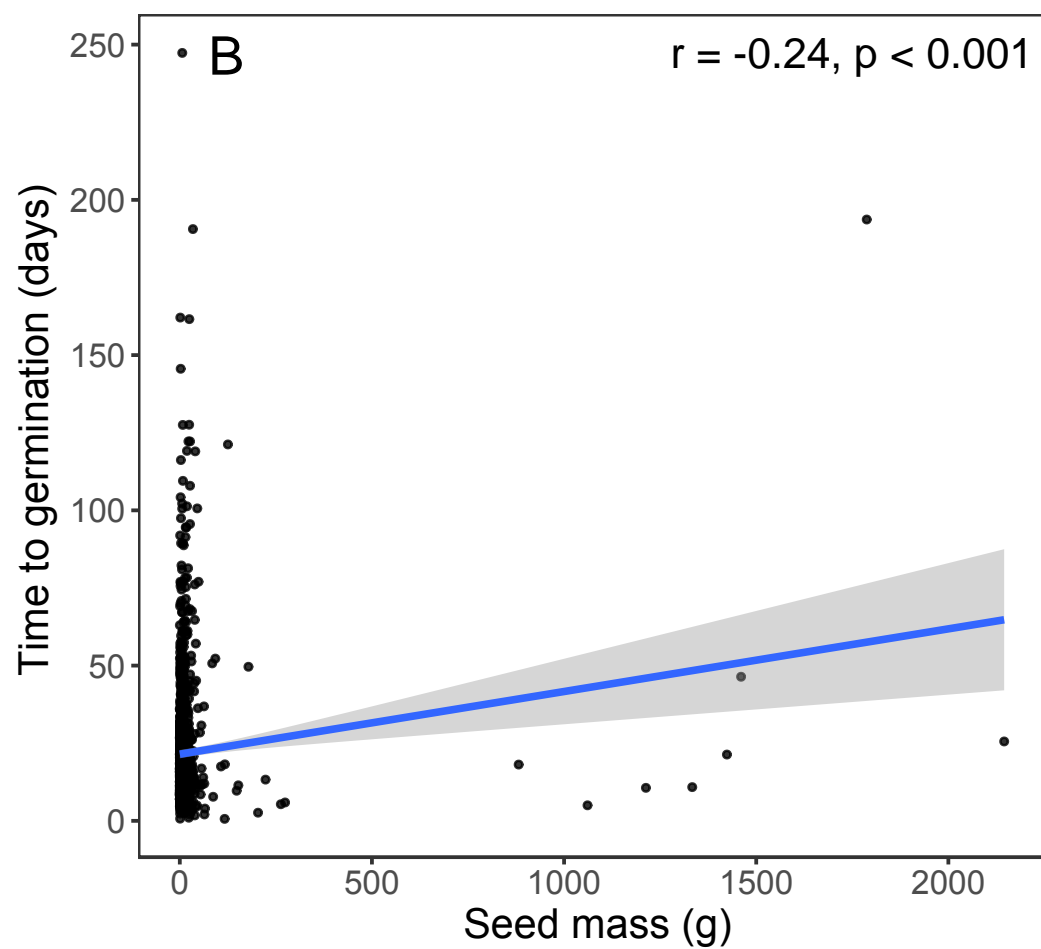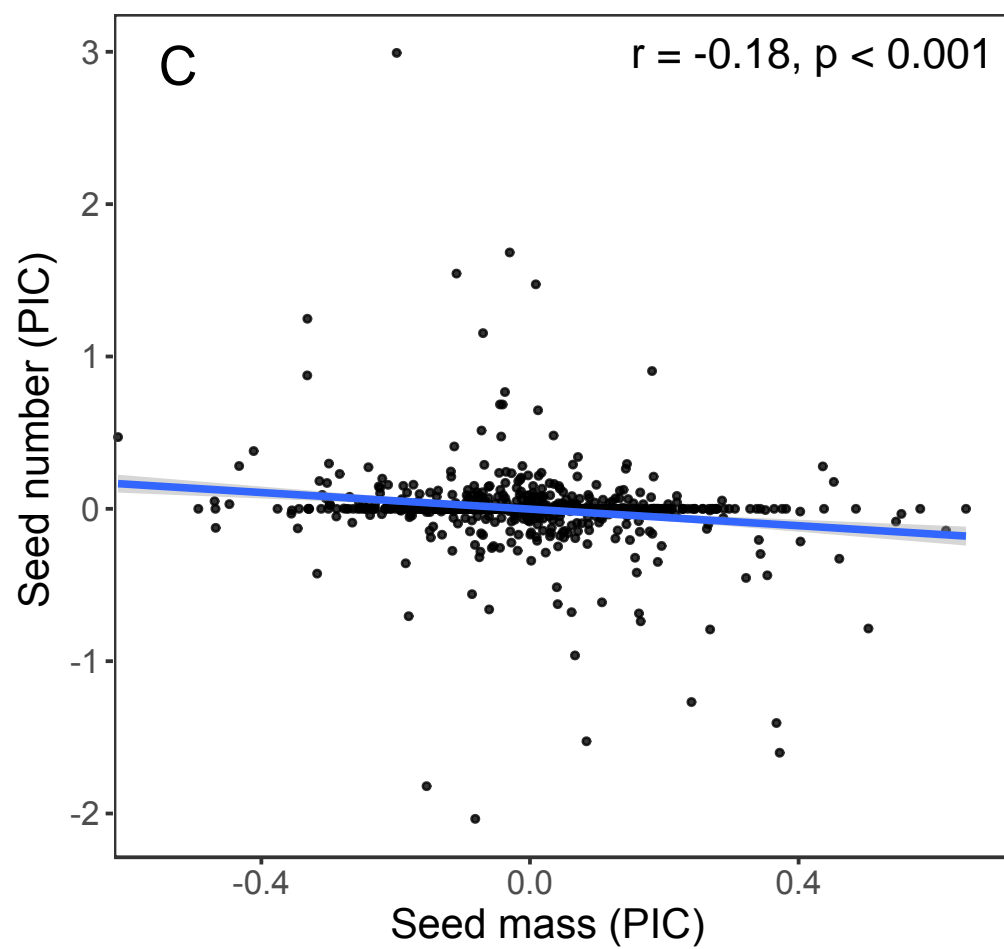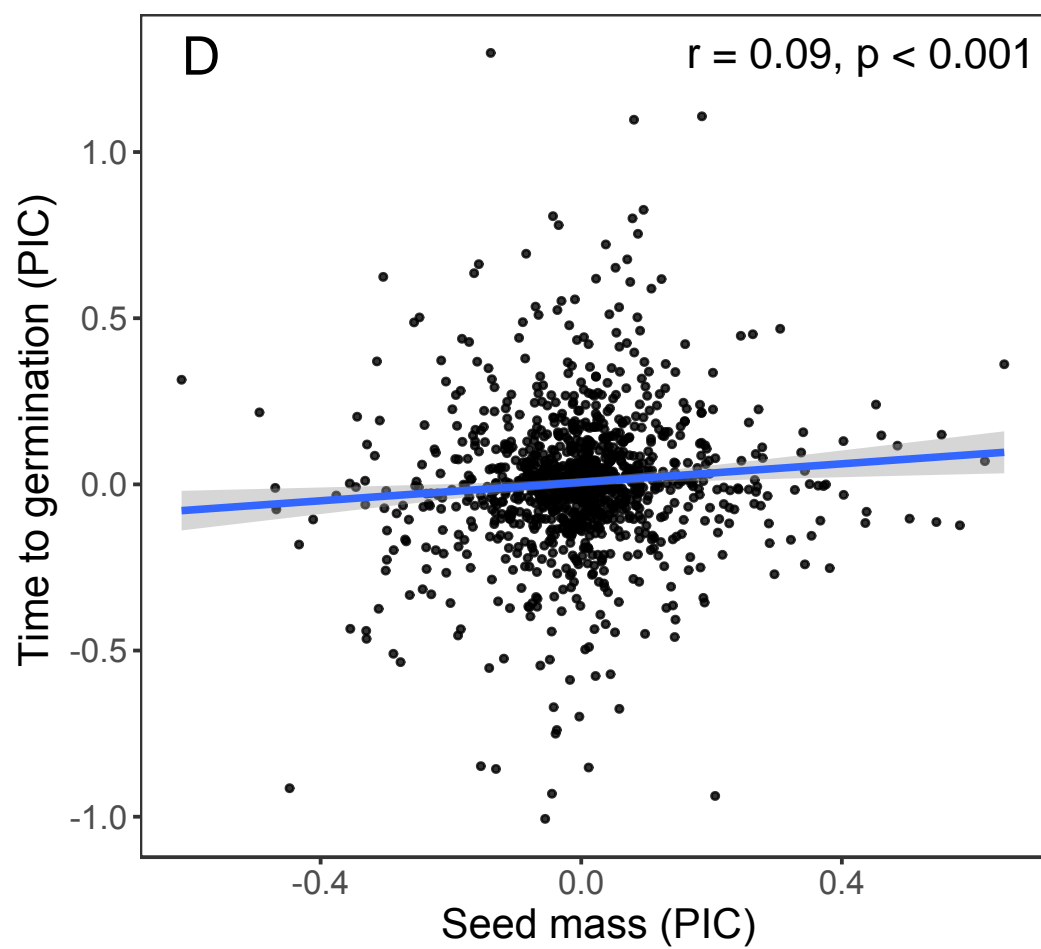

Supplement: Supplementary file 1 [file ECE3-8-2218-s001.pdf]

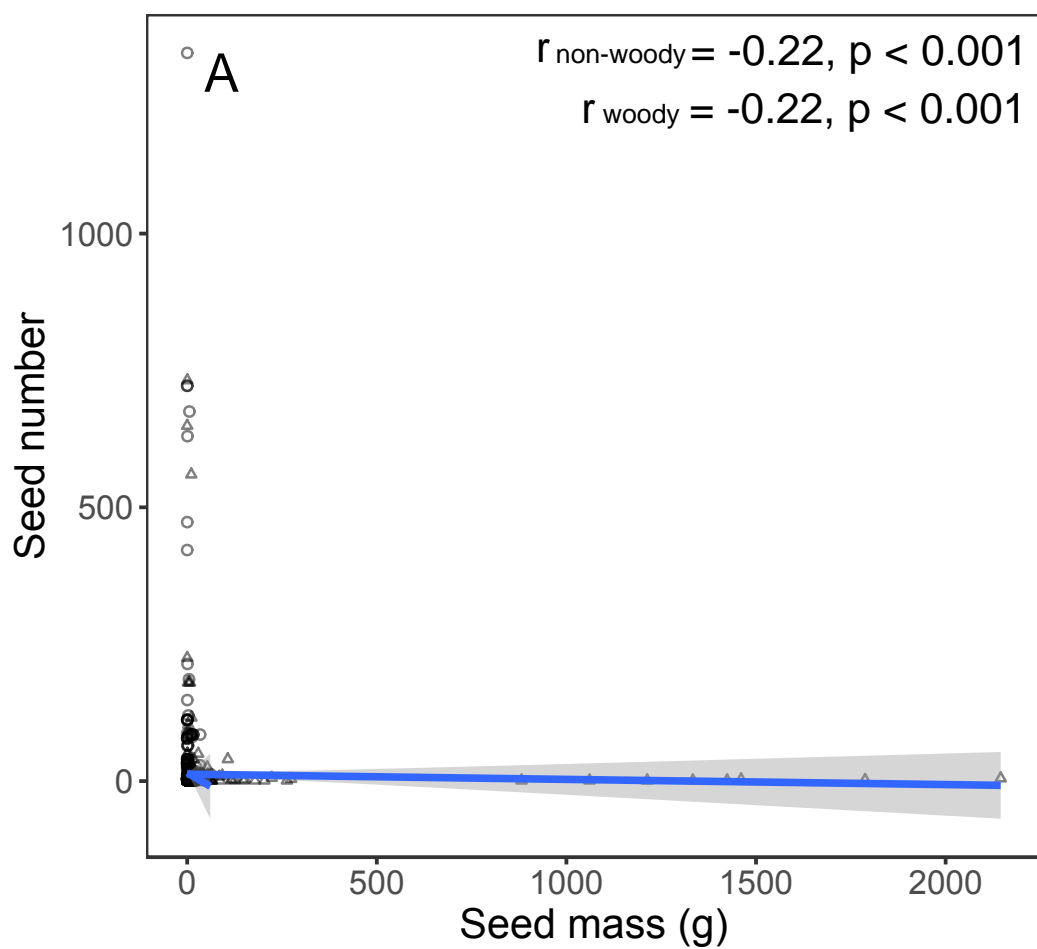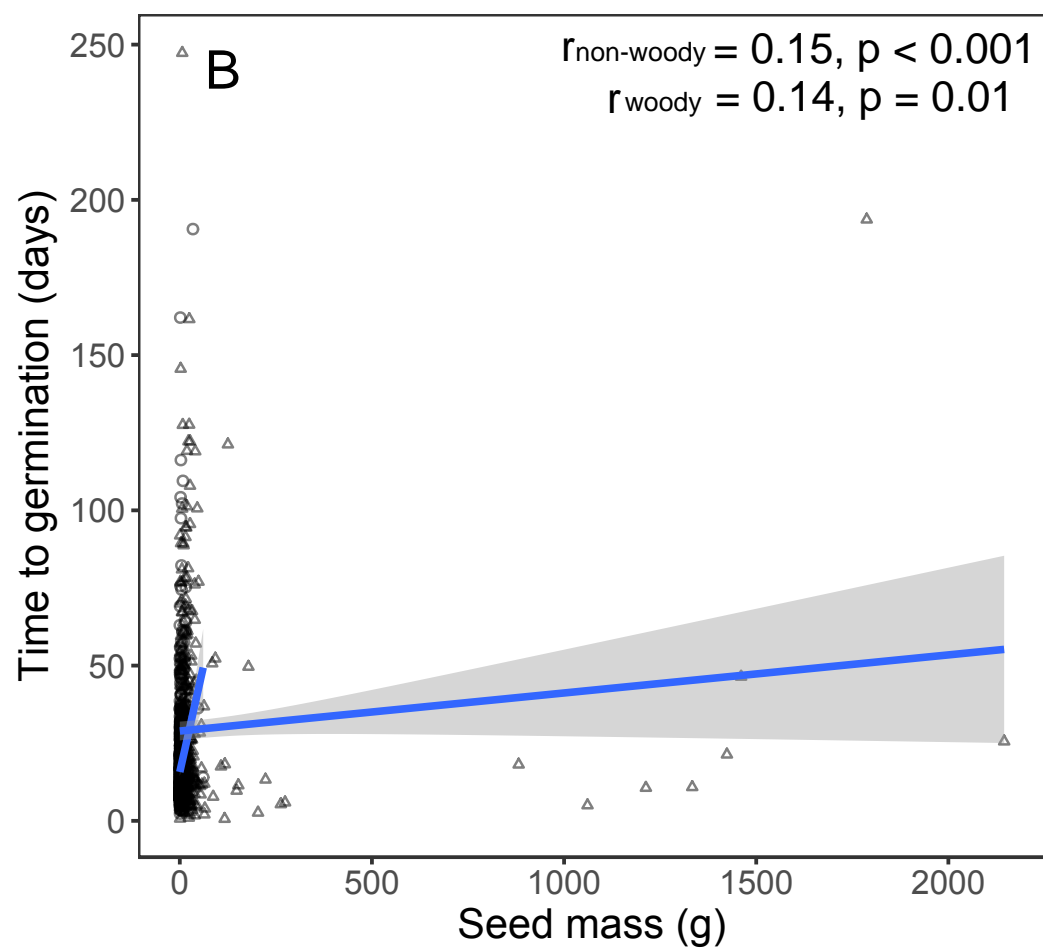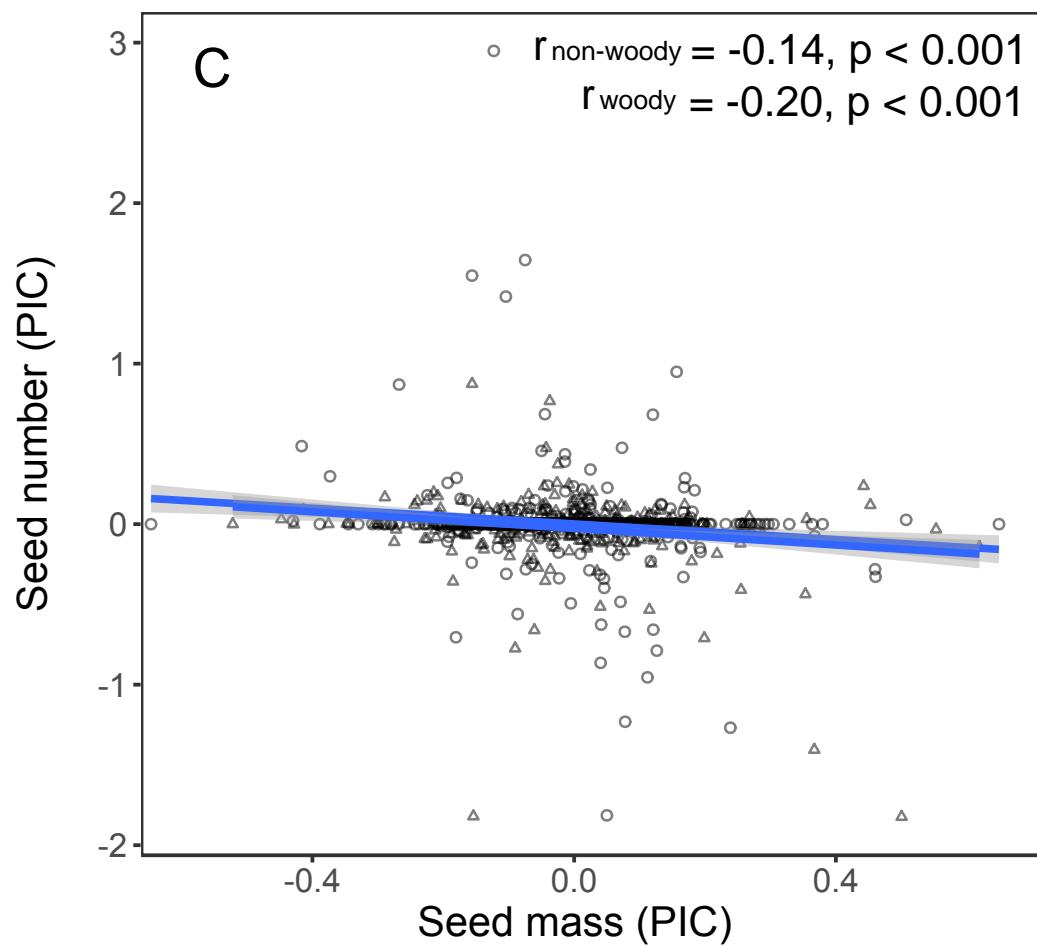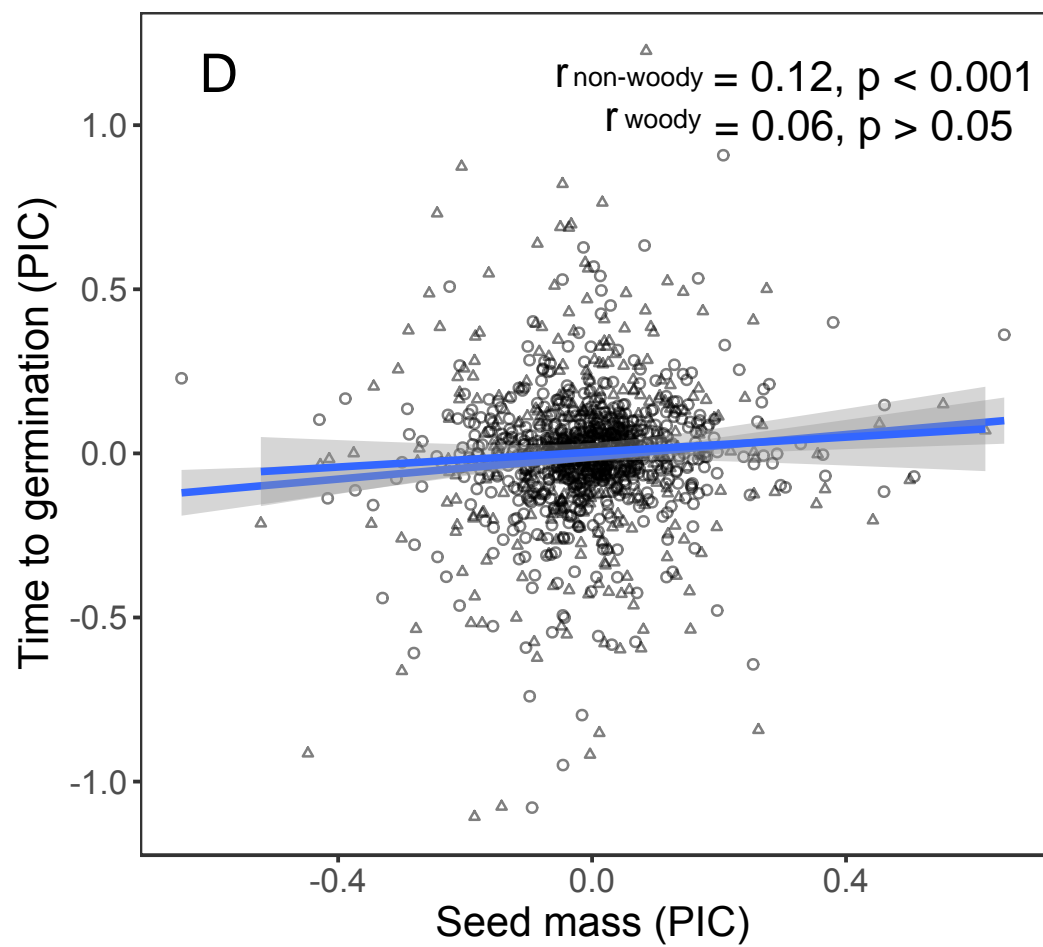

Supplement: Supplementary file 2 [file ECE3-8-2218-s002.pdf]

Seed number

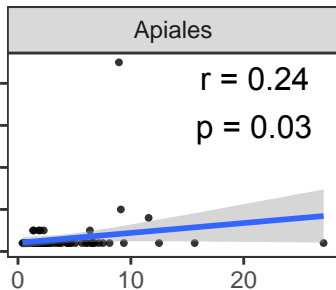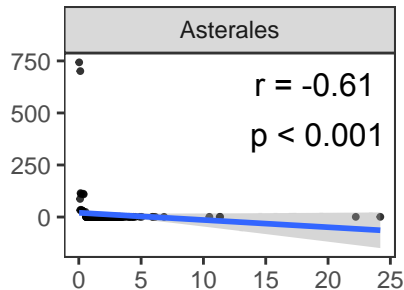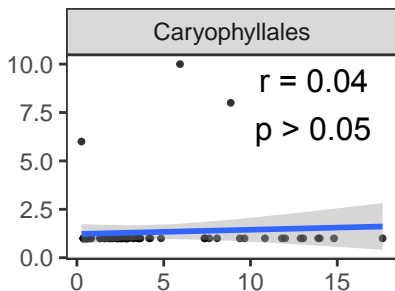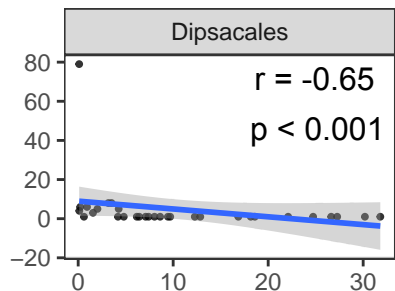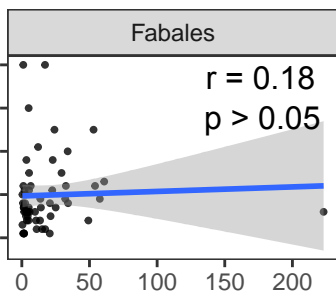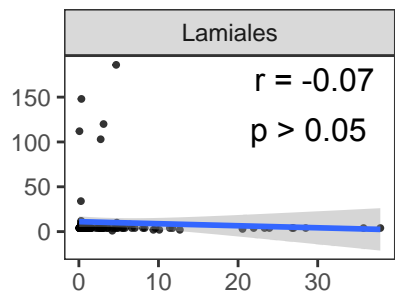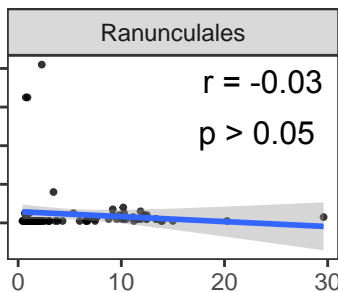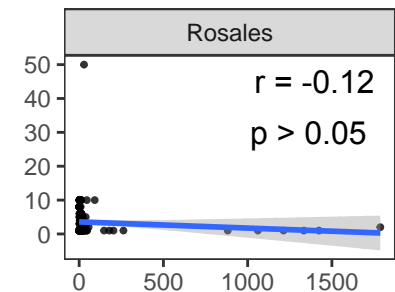

Seed mass(g)

Supplement: Supplementary file 3 [file ECE3-8-2218-s003.pdf]

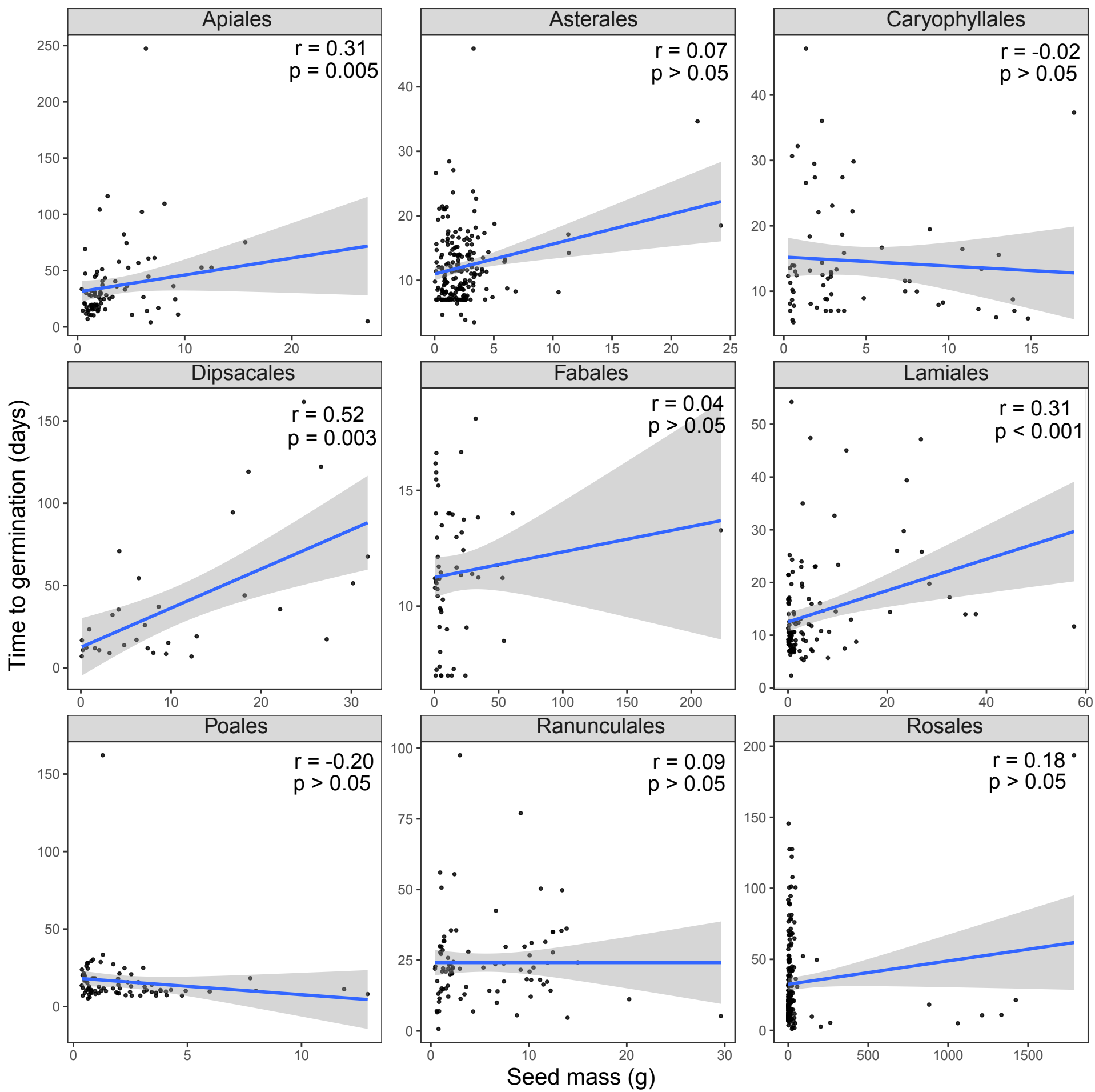

Supplement: Supplementary file 4 [file ECE3-8-2218-s004.pdf]

Seed number

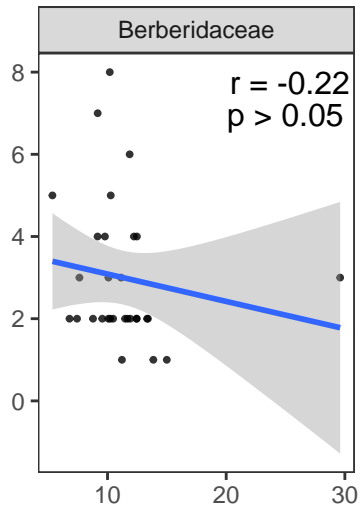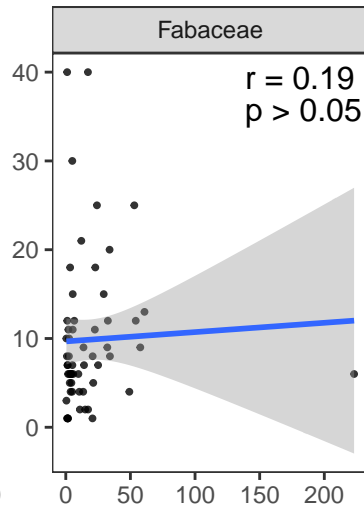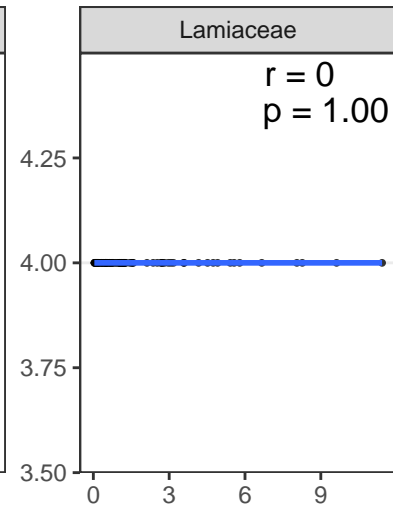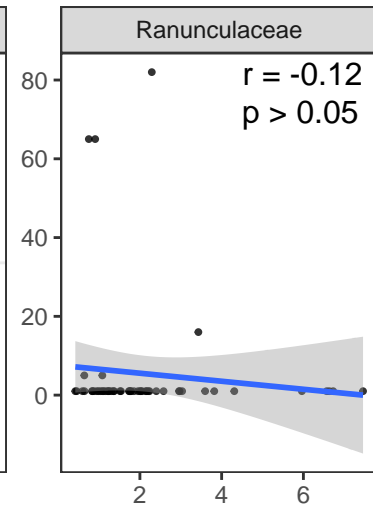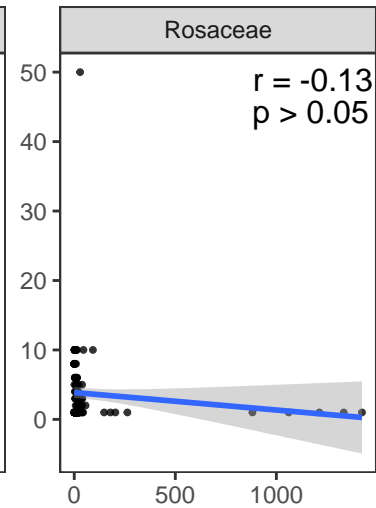

Seed mass(g)

Supplement: Supplementary file 5 [file ECE3-8-2218-s005.pdf]

Time to germination (days)

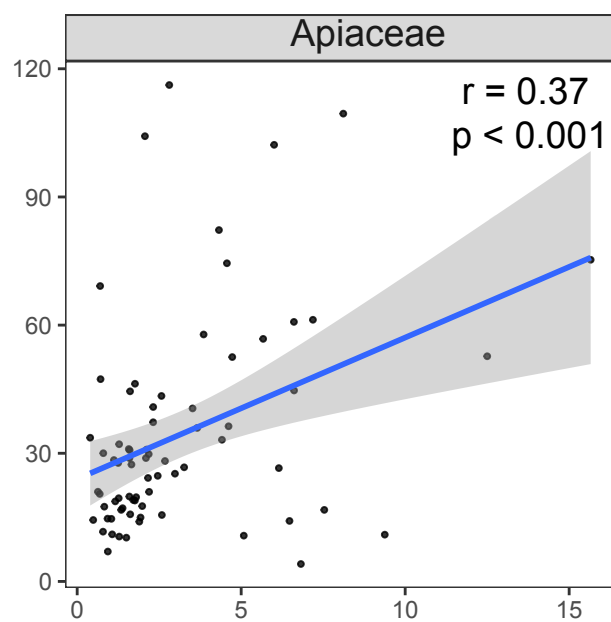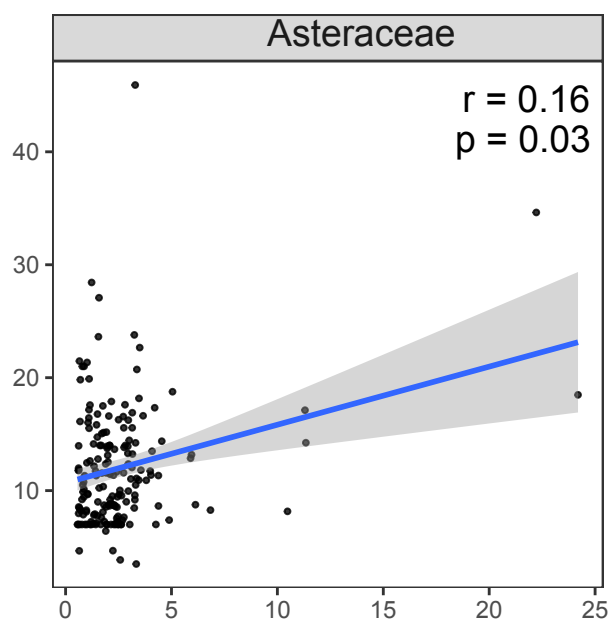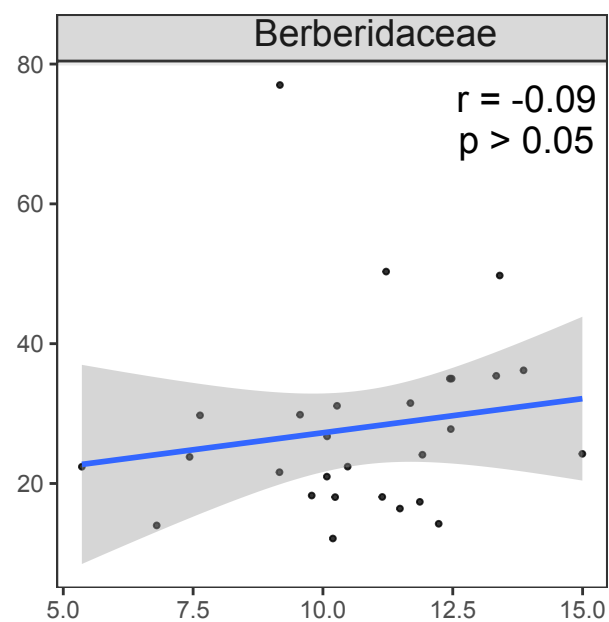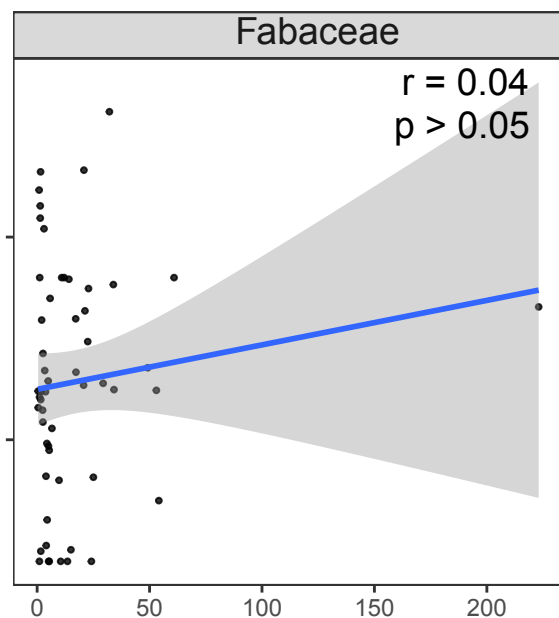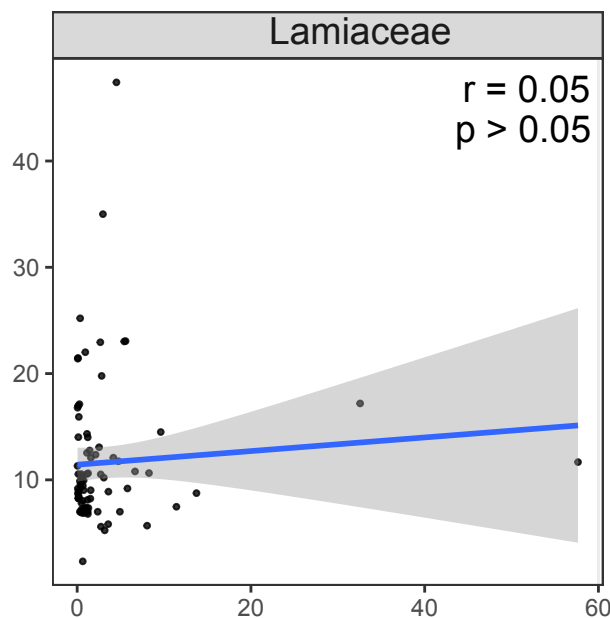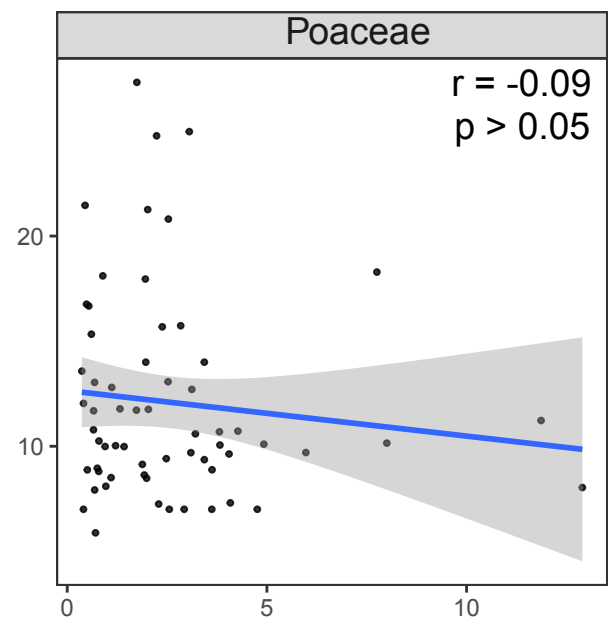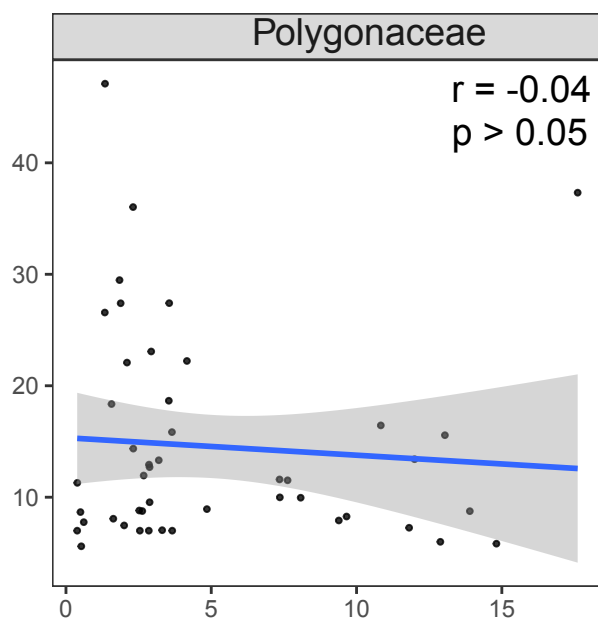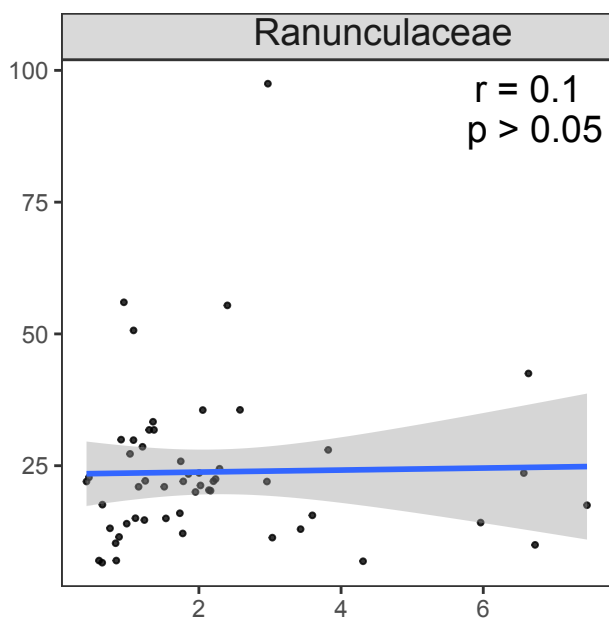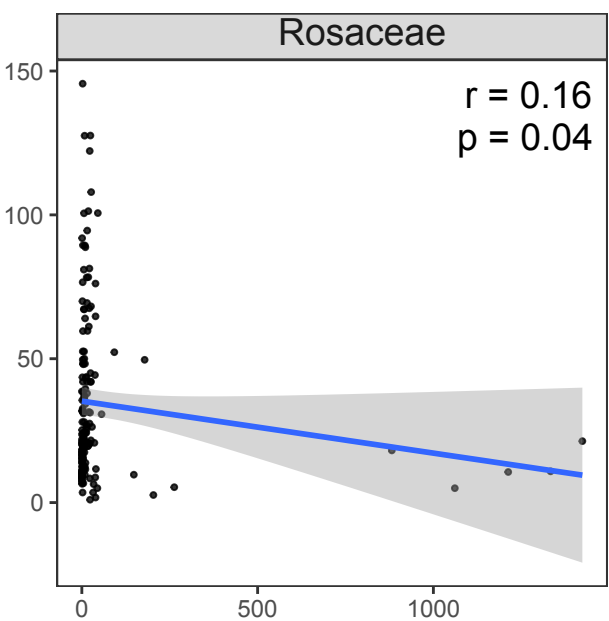

Seed mass (g)

Supplement: Supplementary file 6 [file ECE3-8-2218-s006.pdf]

A

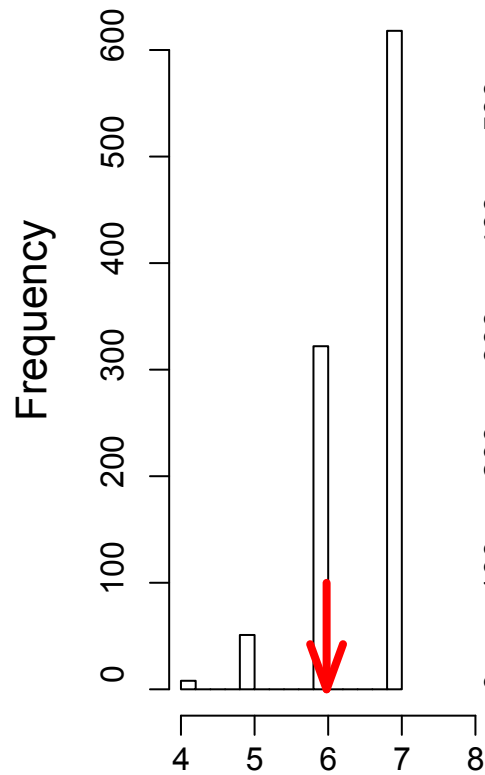

B

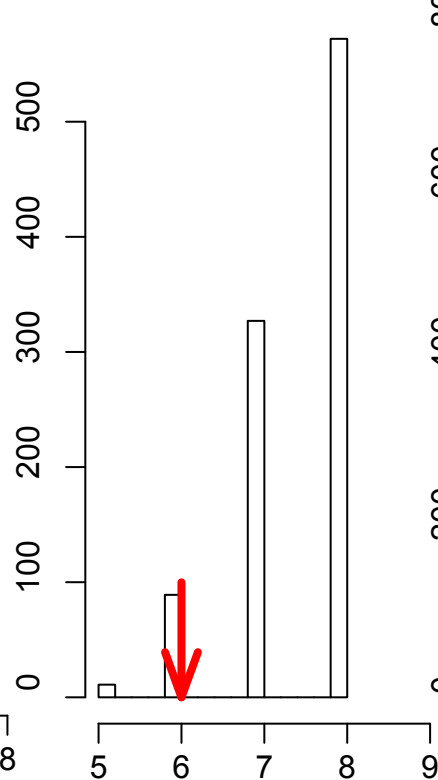

C

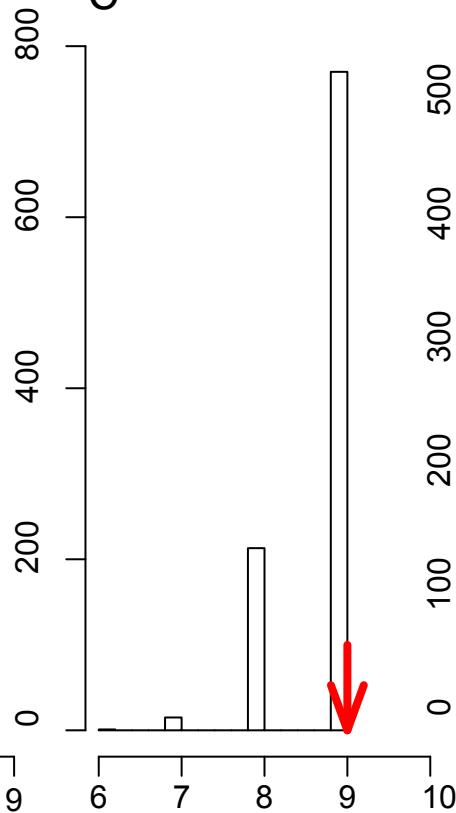

D

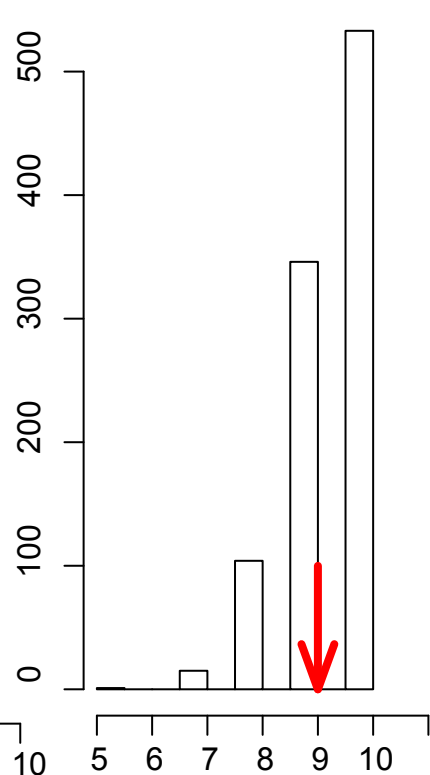

Transitions in randomizations

Supplement: Supplementary file 7 [file ECE3-8-2218-s007.pdf]

A

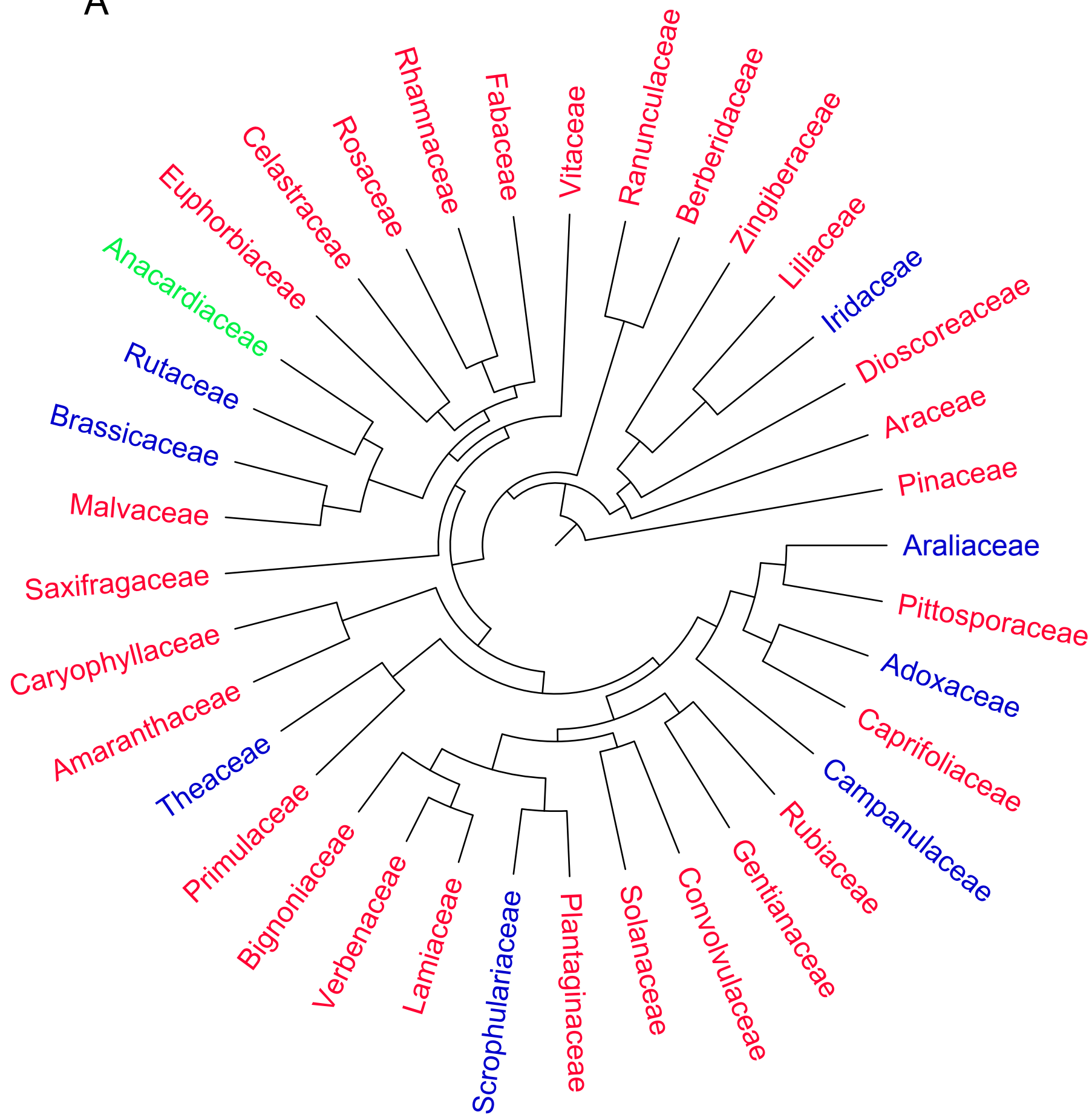

Supplement: Supplementary file 8 [file ECE3-8-2218-s008.pdf]

# B

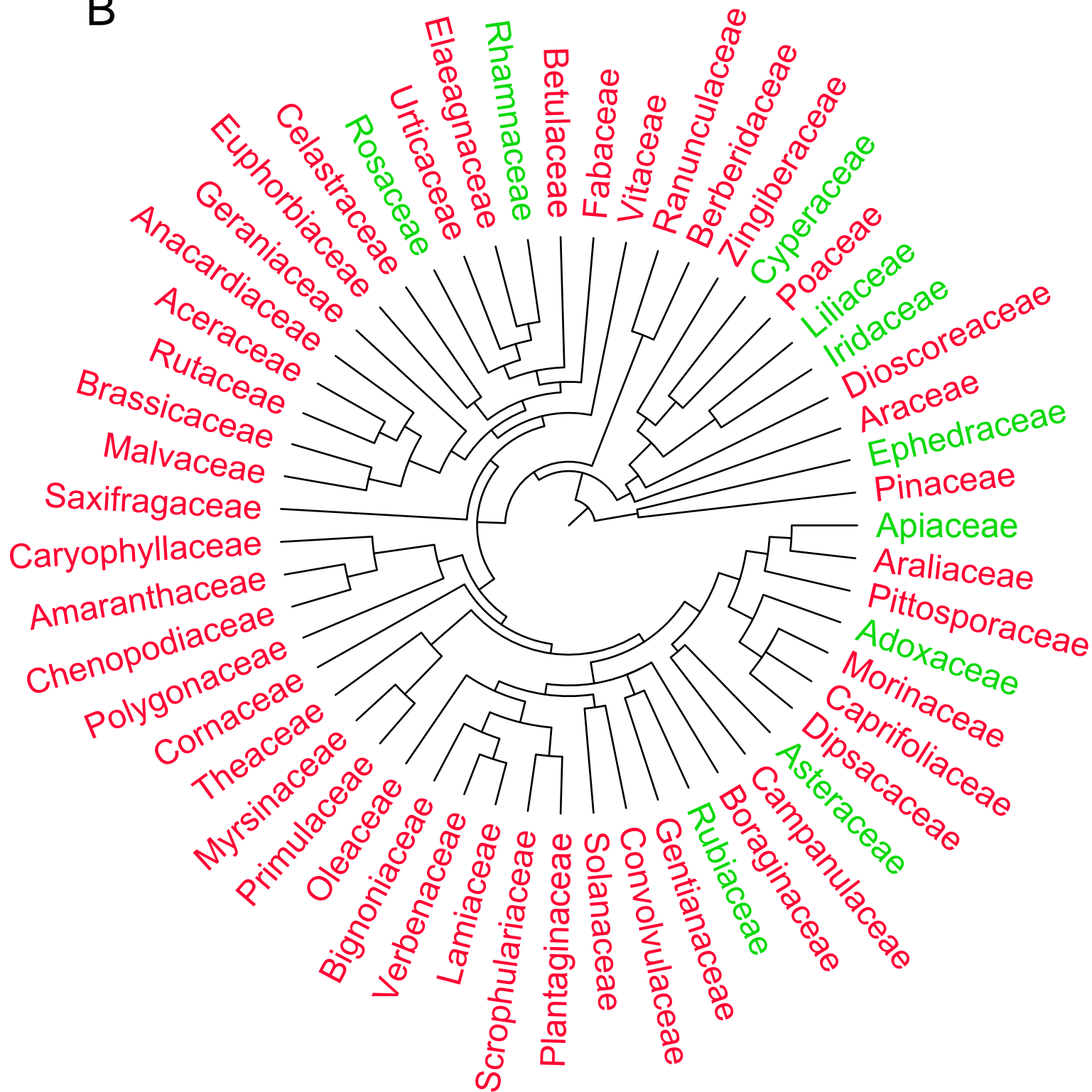

Supplement: Supplementary file 9 [file ECE3-8-2218-s009.pdf]
