# Supplementary material for: Functional trade‐offs and the phylogenetic dispersion of seed traits in a biodiversity hotspot of the Mountains of Southwest China
Source: Ecol Evol. 2018 Jan 25;8(4):2218–30. doi: 10.1002/ece3.3805 (PMC5817125; doi:10.1002/ece3.3805)
Supplement: Supplementary file 10 [file ECE3-8-2218-s010.docx]

**APPENDIX TABLE S1** Germination temperatures of each species in this study. The check marks represent temperatures used in germination tests.

| **SN** | **Species** | **Germination Temperature (daytime/night)** | | | | |
| --- | --- | --- | --- | --- | --- | --- |
|  |  | **15℃/15℃** | **20℃/15℃** | **25℃/15℃** | **30℃/20℃** | **35℃/20℃** |
| 1  2  3  4  5  6  7  8  9  10  11  12  13  14  15  16  17  18  19  20  21  22  23  24  25  26  27  28  29  30  31  32  33  34  35  36  37  38  39  40  41  42  43  44  45  46  47  48  49  50  51  52  53  54  55  56  57  58  59  60  61  62  63  64  65  66  67  68  69  70  71  72  73  74  75  76  77  78  79  80  81  82  83  84  85  86  87  88  89  90  91  92  93  94  95  96  97  98  99  100  101  102  103  104  105  106  107  108  109  110  111  112  113  114  115  116  117  118  119  120  121  122  123  124  125  126  127  128  129  130  131  132  133  134  135  136  137  138  139  140  141  142  143  144  145  146  147  148  149  150  151  152  153  154  155  156  157  158  159  160  161  162  163  164  165  166  167  168  169  170  171  172  173  174  175  176  177  178  179  180  181  182  183  184  185  186  187  188  189  190  191  192  193  194  195  196  197  198  199  200  201  202  203  204  205  206  207  208  209  210  211  212  213  214  215  216  217  218  219  220  221  222  223  224  225  226  227  228  229  230  231  232  233  234  235  236  237  238  239  240  241  242  243  244  245  246  247  248  249  250  251  252  253  254  255  256  257  258  259  260  261  262  263  264  265  266  267  268  269  270  271  272  273  274  275  276  277  278  279  280  281  282  283  284  285  286  287  288  289  290  291  292  293  294  295  296  297  298  299  300  301  302  303  304  305  306  307  308  309  310  311  312  313  314  315  316  317  318  319  320  321  322  323  324  325  326  327  328  329  330  331  332  333  334  335  336  337  338  339  340  341  342  343  344  345  346  347  348  349  350  351  352  353  354  355  356  357  358  359  360  361  362  363  364  365  366  367  368  369  370  371  372  373  374  375  376  377  378  379  380  381  382  383  384  385  386  387  388  389  390  391  392  393  394  395  396  397  398  399  400  401  402  403  404  405  406  407  408  409  410  411  412  413  414  415  416  417  418  419  420  421  422  423  424  425  426  427  428  429  430  431  432  433  434  435  436  437  438  439  440  441  442  443  444  445  446  447  448  449  450  451  452  453  454  455  456  457  458  459  460  461  462  463  464  465  466  467  468  469  470  471  472  473  474  475  476  477  478  479  480  481  482  483  484  485  486  487  488  489  490  491  492  493  494  495  496  497  498  499  500  501  502  503  504  505  506  507  508  509  510  511  512  513  514  515  516  517  518  519  520  521  522  523  524  525  526  527  528  529  530  531  532  533  534  535  536  537  538  539  540  541  542  543  544  545  546  547  548  549  550  551  552  553  554  555  556  557  558  559  560  561  562  563  564  565  566  567  568  569  570  571  572  573  574  575  576  577  578  579  580  581  582  583  584  585  586  587  588  589  590  591  592  593  594  595  596  597  598  599  600  601  602  603  604  605  606  607  608  609  610  611  612  613  614  615  616  617  618  619  620  621  622  623  624  625  626  627  628  629  630  631  632  633  634  635  636  637  638  639  640  641  642  643  644  645  646  647  648  649  650  651  652  653  654  655  656  657  658  659  660  661  662  663  664  665  666  667  668  669  670  671  672  673  674  675  676  677  678  679  680  681  682  683  684  685  586  687  688  689  690  691  692  693  694  695  696  697  698  699  700  701  702  703  704  705  706  707  708  709  710  711  712  713  714  715  716  717  718  719  720  721  722  723  724  725  726  727  728  729  730  731  732  733  734  735  736  737  738  739  740  741  742  743  744  745  746  747  748  749  750  751  752  753  754  755  756  757  758  759  760  761  762  763  764  765  766  767  768  769  770  771  772  773  774  775  776  777  778  779  780  781  782  783  784  785  786  787  788  789  790  791  792  793  794  795  796  797  798  799  800  801  802  803  804  805  806  807  808  809  810  811  812  813  814  815  816  817  818  819  820  821  822  823  824  825  826  827  828  829  830  831  832  833  834  835  836  837  838  839  840  841  842  843  844  845  846  847  848  849  850  851  852  853  854  855  856  857  858  859  860  861  862  863  864  865  866  867  868  869  870  871  872  873  874  875  876  877  878  879  880  881  882  883  884  885  886  887  888  889  890  891  892  893  894  895  896  897  898  899  900  901  902  903  904  905  906  907  908  909  910  911  912  913  914  915  916  917  918  919  920  921  922  923  924  925  926  927  928  929  930  931  932  933  934  935  936  937  938  939  940  941  942  943  944  945  946  947  948  949  950  951  952  953  954  955  956  957  958  959  960  961  962  963  964  965  966  967  968  969  970  971  972  973  974  975  976  977  978  979  980  981  982  983  984  985  986  987  988  989  990  991  992  993  994  995  996  997  998  999  1000  1001  1002  1003  1004  1005  1006  1007  1008  1009  1010  1011  1012  1013  1014  1015  1016  1017  1018  1019  1020  1021  1022  1023  1024  1025  1026  1027  1028  1029  1030  1031  1032  1033  1034  1035  1036  1037  1038  1039  1040  1041  1042  1043  1044  1045  1046  1047  1048  1049  1050  1051  1052  1053  1054  1055  1056  1057  1058  1059  1060  1061  1062  1063  1064  1065  1066  1067  1068  1069  1070  1071  1072  1073  1074  1075  1076  1077  1078  1079  1080  1081  1082  1083  1084  1085  1086  1087  1088  1089  1090  1091  1092  1093  1094  1095  1096  1097  1098  1099  1100  1101  1102  1103  1104  1105  1106  1107  1108  1109  1110  1111  1112  1113  1114  1115  1116  1117  1118  1119 | *Acer amplum*  *Acer caesium*  *Acer cappadocicum*  *Acer caudatum*  *Acer davidii*  *Acer flabellatum*  *Acer kuomeii*  *Acer laxiflorum*  *Acer palmatum*  *Acer sinense*  *Acer stachyophyllum*  *Sambucus adnata*  *Sambucus williamsii*  *Viburnum betulifolium*  *Viburnum chinshanense*  *Viburnum congestum*  *Viburnum cylindricum*  *Viburnum dilatatum*  *Viburnum erubescens*  *Viburnum foetidum*  *Viburnum glomeratum*  *Viburnum hupehense*  *Viburnum punctatum*  *Viburnum utile*  *Achyranthes bidentata*  *Achyranthes longifolia*  *Amaranthus blitum*  *Amaranthus caudatus*  *Amaranthus spinosus*  *Amaranthus tricolor*  *Celosia argentea*  *Cyathula capitata*  *Cyathula officinalis*  *Deeringia amaranthoides*  *Choerospondias axillaris*  *Pistacia chinensis*  *Pistacia weinmanniifolia*  *Rhus chinensis*  *Rhus potaninii*  *Rhus punjabensis*  *Toxicodendron grandiflorum*  *Toxicodendron succedaneum*  *Toxicodendron vernicifluum*  *Toxicodendron wallichii*  *Polyalthia cerasoides*  *Acronema astrantiifolium*  *Acronema schneideri*  *Angelica apaensis*  *Angelica decursiva*  *Angelica laxifoliata*  *Angelica omeiensis*  *Angelica oncosepala*  *Angelica pseudoselinum*  *Angelica sinensis*  *Anthriscus sylvestris*  *Bupleurum candollei*  *Bupleurum commelynoideum*  *Bupleurum hamiltonii*  *Bupleurum marginatum*  *Bupleurum triradiatum*  *Bupleurum yunnanense*  *Carum buriaticum*  *Carum carvi*  *Centella asiatica*  *Chamaesium paradoxum*  *Coriandrum sativum*  *Cyclorhiza waltonii*  *Daucus carota*  *Ferula kingdon-wardii*  *Foeniculum vulgare*  *Heracleum acuminatum*  *Heracleum bivittatum*  *Heracleum candicans*  *Heracleum millefolium*  *Heracleum moellendorffii*  *Heracleum nyalamense*  *Heracleum scabridum*  *Ligusticum acuminatum*  *Ligusticum angelicifolium*  *Ligusticum brachylobum*  *Ligusticum daucoides*  *Ligusticum oliverianum*  *Ligusticum pteridophyllum*  *Ligusticum scapiforme*  *Ligusticum sikiangense*  *Ligusticum thomsonii*  *Notopterygium incisum*  *Oenanthe hookeri*  *Oenanthe javanica*  *Oenanthe sinensis*  *Peucedanum praeruptorum*  *Peucedanum rubricaule*  *Peucedanum turgeniifolium*  *Peucedanum violaceum*  *Physospermopsis muliensis*  *Pimpinella bisinuata*  *Pimpinella candolleana*  *Pimpinella diversifolia*  *Pimpinella rubescens*  *Pimpinella smithii*  *Pleurospermum amabile*  *Pleurospermum angelicoides*  *Pleurospermum aromaticum*  *Pleurospermum franchetianum*  *Pleurospermum hookeri*  *Pleurospermum linearilobum*  *Pleurospermum nanum*  *Pleurospermum pulszkyi*  *Pleurospermum wilsonii*  *Pleurospermum wrightianum*  *Pternopetalum davidii*  *Selinum cryptotaenium*  *Sinolimprichtia alpina*  *Sphallerocarpus gracilis*  *Tongoloa silaifolia*  *Torilis japonica*  *Torilis scabra*  *Vicatia thibetica*  *Ilex polyneura*  *Ilex wattii*  *Arisaema erubescens*  *Arisaema flavum*  *Arisaema heterophyllum*  *Arisaema tortuosum*  *Aralia caesia*  *Aralia chinensis*  *Aralia decaisneana*  *Aralia echinocaulis*  *Brassaiopsis glomerulata*  *Eleutherococcus giraldii*  *Schefflera minutistellata*  *Adenocaulon himalaicum*  *Ainsliaea latifolia*  *Ainsliaea spicata*  *Anaphalis chungtienensis*  *Arctium lappa*  *Arctium tomentosum*  *Aster auriculatus*  *Aster batangensis*  *Aster diplostephioides*  *Aster dolichopodus*  *Aster flaccidus*  *Aster handelii*  *Aster oreophilus*  *Aster souliei*  *Aster tataricus*  *Aster tongolensis*  *Aster yunnanensis*  *Bidens biternata*  *Bidens parviflora*  *Bidens pilosa*  *Bidens tripartita*  *Carduus acanthoides*  *Carduus crispus*  *Carduus nutans*  *Carpesium cernuum*  *Carpesium divaricatum*  *Carpesium lipskyi*  *Carpesium nepalense*  *Carpesium scapiforme*  *Carpesium triste*  *Chaetoseris dolichophylla*  *Cirsium arvense*  *Cirsium eriophoroides*  *Cirsium henryi*  *Cirsium japonicum*  *Cirsium leo*  *Cirsium periacanthaceum*  *Cirsium shansiense*  *Cirsium souliei*  *Cremanthodium brachychaetum*  *Cremanthodium calcicola*  *Cremanthodium campanulatum*  *Cremanthodium citriflorum*  *Cremanthodium ellisii*  *Cremanthodium helianthus*  *Cremanthodium lineare*  *Cremanthodium principis*  *Cremanthodium reniforme*  *Cremanthodium stenactinium*  *Cremanthodium suave*  *Dolomiaea calophylla*  *Dolomiaea souliei*  *Doronicum oblongifolium*  *Doronicum stenoglossum*  *Dubyaea amoena*  *Erigeron breviscapus*  *Eupatorium japonicum*  *Gerbera nivea*  *Gerbera piloselloides*  *Gynura cusimbua*  *Hieracium umbellatum*  *Inula racemosa*  *Leibnitzia anandria*  *Leibnitzia pusilla*  *Leibnitzia ruficoma*  *Ligularia achyrotricha*  *Ligularia alatipes*  *Ligularia atroviolacea*  *Ligularia brassicoides*  *Ligularia confertiflora*  *Ligularia cremanthodioides*  *Ligularia cyathiceps*  *Ligularia cymbulifera*  *Ligularia dictyoneura*  *Ligularia duciformis*  *Ligularia fangiana*  *Ligularia fischeri*  *Ligularia hodgsonii*  *Ligularia hookeri*  *Ligularia intermedia*  *Ligularia kanaitzensis*  *Ligularia konkalingensis*  *Ligularia lamarum*  *Ligularia lankongensis*  *Ligularia lapathifolia*  *Ligularia latihastata*  *Ligularia liatroides*  *Ligularia longihastata*  *Ligularia melanocephala*  *Ligularia muliensis*  *Ligularia myriocephala*  *Ligularia nelumbifolia*  *Ligularia odontomanes*  *Ligularia pleurocaulis*  *Ligularia potaninii*  *Ligularia przewalskii*  *Ligularia purdomii*  *Ligularia rumicifolia*  *Ligularia sagitta*  *Ligularia sibirica*  *Ligularia stenoglossa*  *Ligularia subspicata*  *Ligularia tenuicaulis*  *Ligularia tongolensis*  *Ligularia tsangchanensis*  *Ligularia veitchiana*  *Ligularia vellerea*  *Ligularia virgaurea*  *Ligularia yunnanensis*  *Olgaea tangutica*  *Parasenecio latipes*  *Parasenecio palmatisectus*  *Parasenecio roborowskii*  *Pertya berberidoides*  *Pertya phylicoides*  *Picris hieracioides*  *Picris japonica*  *Saussurea arenaria*  *Saussurea caudata*  *Saussurea cochlearifolia*  *Saussurea columnaris*  *Saussurea dzeurensis*  *Saussurea epilobioides*  *Saussurea erubescens*  *Saussurea globosa*  *Saussurea graminea*  *Saussurea graminifolia*  *Saussurea gyacaensis*  *Saussurea hieracioides*  *Saussurea integrifolia*  *Saussurea iodostegia*  *Saussurea japonica*  *Saussurea katochaete*  *Saussurea laniceps*  *Saussurea leontodontoides*  *Saussurea leucoma*  *Saussurea likiangensis*  *Saussurea longifolia*  *Saussurea loriformis*  *Saussurea macrota*  *Saussurea neofranchetii*  *Saussurea nigrescens*  *Saussurea nyalamensis*  *Saussurea obvallata*  *Saussurea pachyneura*  *Saussurea phaeantha*  *Saussurea pinetorum*  *Saussurea pinnatidentata*  *Saussurea polycolea*  *Saussurea poochlamys*  *Saussurea populifolia*  *Saussurea przewalskii*  *Saussurea retroserrata*  *Saussurea romuleifolia*  *Saussurea scabrida*  *Saussurea semifasciata*  *Saussurea semilyrata*  *Saussurea stella*  *Saussurea stoliczkae*  *Saussurea stricta*  *Saussurea subulata*  *Saussurea subulisquama*  *Saussurea sylvatica*  *Saussurea tatsienensis*  *Saussurea vestita*  *Saussurea yunnanensis*  *Senecio megalanthus*  *Senecio muliensis*  *Soroseris erysimoides*  *Syncalathium souliei*  *Synotis erythropappa*  *Synurus deltoides*  *Taraxacum borealisinense*  *Taraxacum chionophilum*  *Taraxacum dasypodum*  *Taraxacum lanigerum*  *Taraxacum leucanthum*  *Taraxacum lugubre*  *Youngia paleacea*  *Impatiens lateristachys*  *Berberis agricola*  *Berberis amoena*  *Berberis approximata*  *Berberis concinna*  *Berberis davidii*  *Berberis dictyoneura*  *Berberis dictyophylla*  *Berberis ferdinandi-coburgii*  *Berberis franchetiana*  *Berberis gyalaica*  *Berberis henryana*  *Berberis ignorata*  *Berberis insignis*  *Berberis jamesiana*  *Berberis julianae*  *Berberis kansuensis*  *Berberis lecomtei*  *Berberis metapolyantha*  *Berberis minutiflora*  *Berberis muliensis*  *Berberis obovatifolia*  *Berberis papillifera*  *Berberis pruinosa*  *Berberis reticulinervis*  *Berberis sublevis*  *Berberis tsarongensis*  *Berberis wilsonae*  *Berberis yunnanensis*  *Mahonia fortunei*  *Nandina domestica*  *Alnus cremastogyne*  *Alnus nepalensis*  *Betula platyphylla*  *Betula potaninii*  *Catalpa ovata*  *Incarvillea arguta*  *Incarvillea lutea*  *Incarvillea mairei*  *Antiotrema dunnianum*  *Cynoglossum amabile*  *Cynoglossum lanceolatum*  *Microula floribunda*  *Microula forrestii*  *Microula sikkimensis*  *Onosma exsertum*  *Onosma multiramosum*  *Onosma paniculatum*  *Onosma sinicum*  *Onosma waddellii*  *Arabis hirsuta*  *Capsella bursa-pastoris*  *Cardamine impatiens*  *Cardamine macrophylla*  *Cardamine scutata*  *Cardamine tangutorum*  *Cardamine yunnanensis*  *Megacarpaea delavayi*  *Megacarpaea polyandra*  *Thlaspi arvense*  *Sarcococca hookeriana*  *Calycanthus floridus*  *Adenophora khasiana*  *Campanula chinensis*  *Campanula pallida*  *Codonopsis convolvulacea*  *Codonopsis foetens*  *Codonopsis gombalana*  *Codonopsis purpurea*  *Cyananthus delavayi*  *Cyananthus formosus*  *Cyananthus hookeri*  *Cyananthus incanus*  *Cyananthus inflatus*  *Cyananthus longiflorus*  *Cyananthus macrocalyx*  *Cyananthus microphyllus*  *Cannabis sativa*  *Lonicera caerulea*  *Lonicera cyanocarpa*  *Lonicera ligustrina*  *Lonicera maackii*  *Arenaria yunnanensis*  *Silene himalayensis*  *Stellaria yunnanensis*  *Celastrus angulatus*  *Celastrus gemmatus*  *Celastrus rosthornianus*  *Euonymus grandiflorus*  *Euonymus nanoides*  *Acroglochin persicarioides*  *Chenopodium album*  *Chenopodium ficifolium*  *Chenopodium glaucum*  *Chenopodium hybridum*  *Microgynoecium tibeticum*  *Circaeaster agrestis*  *Hypericum monogynum*  *Commelina paludosa*  *Cuscuta chinensis*  *Cuscuta europaea*  *Dinetus racemosus*  *Ipomoea purpurea*  *Merremia hederacea*  *Coriaria nepalensis*  *Coriaria terminalis*  *Cornus capitata*  *Cornus hemsleyi*  *Cornus macrophylla*  *Cornus oblonga*  *Platycladus orientalis*  *Blysmus sinocompressus*  *Carex atrata*  *Carex baccans*  *Carex composita*  *Carex cruciata*  *Carex filicina*  *Carex haematostoma*  *Carex hirtelloides*  *Carex kansuensis*  *Carex laeta*  *Carex lehmannii*  *Carex schneideri*  *Eleocharis yokoscensis*  *Fimbristylis ovata*  *Kobresia cuneata*  *Kobresia kansuensis*  *Kobresia setschwanensis*  *Schoenoplectus tabernaemontani*  *Dioscorea bulbifera*  *Dioscorea collettii*  *Dioscorea hemsleyi*  *Dioscorea kamoonensis*  *Dipsacus asper*  *Dipsacus atratus*  *Dipsacus chinensis*  *Dipsacus inermis*  *Dipsacus japonicus*  *Pterocephalus bretschneideri*  *Pterocephalus hookeri*  *Diospyros lotus*  *Elaeagnus delavayi*  *Elaeagnus lanceolata*  *Elaeagnus umbellata*  *Hippophae neurocarpa*  *Hippophae rhamnoides*  *Hippophae tibetana*  *Ephedra gerardiana*  *Ephedra likiangensis*  *Ephedra minuta*  *Rhododendron decorum*  *Rhododendron yunnanense*  *Eucommia ulmoides*  *Euphorbia esula*  *Euphorbia fischeriana*  *Euphorbia griffithii*  *Euphorbia jolkinii*  *Euphorbia micractina*  *Euphorbia stracheyi*  *Excoecaria acerifolia*  *Macaranga indica*  *Phyllanthus cochinchinensis*  *Phyllanthus emblica*  *Triadica sebifera*  *Euptelea pleiosperma*  *Acacia pennata*  *Apios carnea*  *Astragalus adsurgens*  *Astragalus degensis*  *Astragalus ernestii*  *Astragalus floridus*  *Astragalus monbeigii*  *Astragalus strictus*  *Astragalus tongolensis*  *Bauhinia brachycarpa*  *Cajanus cajan*  *Crotalaria pallida*  *Crotalaria sessiliflora*  *Dalbergia mimosoides*  *Dendrolobium triangulare*  *Desmodium elegans*  *Desmodium heterocarpon*  *Desmodium multiflorum*  *Desmodium sequax*  *Desmodium styracifolium*  *Desmodium yunnanense*  *Flemingia latifolia*  *Hedysarum citrinum*  *Hedysarum tanguticum*  *Indigofera amblyantha*  *Indigofera balfouriana*  *Indigofera delavayi*  *Indigofera lenticellata*  *Indigofera nigrescens*  *Indigofera pendula*  *Kummerowia striata*  *Lespedeza bicolor*  *Lespedeza pilosa*  *Leucaena leucocephala*  *Lotus corniculatus*  *Medicago edgeworthii*  *Medicago lupulina*  *Medicago minima*  *Melilotus indicus*  *Piptanthus nepalensis*  *Rhynchosia minima*  *Robinia pseudoacacia*  *Salweenia wardii*  *Senna occidentalis*  *Senna tora*  *Sophora davidii*  *Sophora velutina*  *Thermopsis barbata*  *Thermopsis lanceolata*  *Tibetia himalaica*  *Trifolium repens*  *Vicia bungei*  *Vicia cracca*  *Vicia unijuga*  *Vigna vexillata*  *Gentiana atuntsiensis*  *Gentiana macrophylla*  *Gentianopsis paludosa*  *Swertia macrosperma*  *Veratrilla baillonii*  *Geranium erianthum*  *Geranium nepalense*  *Geranium pseudosibiricum*  *Geranium strictipes*  *Geranium wilfordii*  *Iris bulleyana*  *Iris chrysographes*  *Iris clarkei*  *Iris delavayi*  *Iris goniocarpa*  *Iris japonica*  *Iris lactea*  *Iris ruthenica*  *Iris subdichotoma*  *Triglochin palustris*  *Ajuga lupulina*  *Albizia bracteata*  *Albizia kalkora*  *Albizia lucidior*  *Anisomeles indica*  *Chelonopsis souliei*  *Clinopodium chinense*  *Clinopodium confine*  *Clinopodium gracile*  *Clinopodium megalanthum*  *Clinopodium polycephalum*  *Clinopodium repens*  *Clinopodium urticifolium*  *Craniotome furcata*  *Dracocephalum calophyllum*  *Dracocephalum heterophyllum*  *Dracocephalum propinquum*  *Dracocephalum tanguticum*  *Elsholtzia bodinieri*  *Elsholtzia capituligera*  *Elsholtzia ciliata*  *Elsholtzia cyprianii*  *Elsholtzia densa*  *Elsholtzia eriostachya*  *Elsholtzia flava*  *Elsholtzia fruticosa*  *Elsholtzia pilosa*  *Elsholtzia souliei*  *Elsholtzia strobilifera*  *Eriophyton wallichii*  *Galeopsis bifida*  *Geniosporum coloratum*  *Isodon bulleyanus*  *Isodon coetsa*  *Isodon hispidus*  *Isodon japonicus*  *Isodon lophanthoides*  *Isodon pharicus*  *Isodon sculponeatus*  *Lagopsis supina*  *Lamiophlomis rotata*  *Lamium barbatum*  *Leonurus japonicus*  *Leonurus sibiricus*  *Leucas ciliata*  *Melissa axillaris*  *Microtoena delavayi*  *Mosla cavaleriei*  *Nepeta cataria*  *Nepeta laevigata*  *Nepeta prattii*  *Nepeta sibirica*  *Nepeta souliei*  *Nepeta stewartiana*  *Nepeta tenuiflora*  *Notochaete hamosa*  *Ocimum gratissimum*  *Origanum vulgare*  *Perilla frutescens*  *Phlomis agraria*  *Phlomis atropurpurea*  *Phlomis medicinalis*  *Phlomis setifera*  *Phlomis strigosa*  *Phlomis tibetica*  *Phlomis younghushandii*  *Pogostemon brevicorollus*  *Pogostemon glaber*  *Pogostemon nigrescens*  *Prunella hispida*  *Prunella vulgaris*  *Salvia castanea*  *salvia digitaloides*  *Salvia flava*  *Salvia japonica*  *Salvia prattii*  *Salvia przewalskii*  *Salvia roborowskii*  *Salvia smithii*  *Salvia subpalmatinervis*  *Salvia trijuga*  *Salvia yunnanensis*  *Scutellaria chungtienensis*  *Allium beesianum*  *Allium carolinianum*  *Allium cyaneum*  *Allium cyathophorum*  *Allium fasciculatum*  *Allium forrestii*  *Allium hookeri*  *Allium macranthum*  *Allium mairei*  *Allium ovalifolium*  *Allium prattii*  *Allium przewalskianum*  *Allium ramosum*  *Allium rude*  *Allium sikkimense*  *Allium tanguticum*  *Allium tuberosum*  *Allium wallichii*  *Asparagus filicinus*  *Asparagus meioclados*  *Asparagus setaceus*  *Eremurus chinensis*  *Lilium lophophorum*  *Polygonatum cirrhifolium*  *Polygonatum verticillatum*  *Manglietia insignis*  *Aspidopterys glabriuscula*  *Abutilon indicum*  *Abutilon paniculatum*  *Abutilon theophrasti*  *Hibiscus trionum*  *Kydia calycina*  *Malva verticillata*  *Triumfetta cana*  *Triumfetta pilosa*  *Triumfetta rhomboidea*  *Urena lobata*  *Sinomenium acutum*  *Stephania hernandiifolia*  *Broussonetia papyrifera*  *Ficus chapaensis*  *Acanthocalyx alba*  *Morina chinensis*  *Morina kokonorica*  *Ardisia virens*  *Embelia floribunda*  *Embelia ribes*  *Myrsine africana*  *Myrsine semiserrata*  *Oxybaphus himalaicus*  *Camptotheca acuminata*  *Nyssa sinensis*  *Fraxinus chinensis*  *Fraxinus sikkimensis*  *Jasminum humile*  *Jasminum officinale*  *Jasminum subhumile*  *Ligustrum compactum*  *Ligustrum confusum*  *Ligustrum delavayanum*  *Ligustrum lucidum*  *Ligustrum quihoui*  *Ligustrum sempervirens*  *Syringa yunnanensis*  *Adenia cardiophylla*  *Phryma leptostachya*  *Phytolacca acinosa*  *Phytolacca americana*  *Abies squamata*  *Picea asperata*  *Picea likiangensis*  *Picea purpurea*  *Pinus densata*  *Piper mullesua*  *Piper thomsonii*  *Pittosporum brevicalyx*  *Pittosporum crispulum*  *Pittosporum heterophyllum*  *Plantago asiatica*  *Plantago cavaleriei*  *Plantago depressa*  *Plantago gentianoides*  *Plantago major*  *Plumbagella micrantha*  *Achnatherum inebrians*  *Achnatherum sibiricum*  *Aristida triseta*  *Arundinella hirta*  *Avena chinensis*  *Avena fatua*  *Avena sativa*  *Beckmannia syzigachne*  *Bothriochloa ischaemum*  *Brachypodium pratense*  *Brachypodium sylvaticum*  *Bromus japonicus*  *Bromus plurinodis*  *Bromus porphyranthos*  *Bromus remotiflorus*  *Bromus sinensis*  *Bromus tectorum*  *Capillipedium assimile*  *Capillipedium parviflorum*  *Cymbopogon distans*  *Cymbopogon goeringii*  *Dactylis glomerata*  *Deyeuxia flavens*  *Deyeuxia pulchella*  *Digitaria cruciata*  *Digitaria sanguinalis*  *Echinochloa colona*  *Echinochloa crusgalli*  *Echinochloa frumentacea*  *Elymus antiquus*  *Elymus dahuricus*  *Elymus excelsus*  *Elymus nutans*  *Elymus sibiricus*  *Eragrostis atrovirens*  *Eulalia speciosa*  *Festuca gigantea*  *Festuca leptopogon*  *Festuca rubra*  *Festuca vierhapperi*  *Festuca yunnanensis*  *Heteropogon contortus*  *Ischaemum barbatum*  *Lolium perenne*  *Lophatherum gracile*  *Pennisetum alopecuroides*  *Pennisetum flaccidum*  *Phaenosperma globosa*  *Piptatherum munroi*  *Poa alpina*  *Poa perennis*  *Polypogon fugax*  *Ptilagrostis dichotoma*  *Schizachyrium delavayi*  *Setaria pumila*  *Setaria viridis*  *Setaria yunnanensis*  *Stipa capillacea*  *Stipa penicillata*  *Stipa purpurea*  *Stipa regeliana*  *Stipa roborowskyi*  *Antenoron filiforme*  *Fagopyrum esculentum*  *Fagopyrum gracilipes*  *Fallopia aubertii*  *Fallopia dumetorum*  *Fallopia multiflora*  *Oxyria digyna*  *Oxyria sinensis*  *Polygonum amphibium*  *Polygonum aviculare*  *Polygonum capitatum*  *Polygonum cathayanum*  *Polygonum chinense*  *Polygonum forrestii*  *Polygonum glaciale*  *Polygonum griffithii*  *Polygonum hydropiper*  *Polygonum jucundum*  *Polygonum lapathifolium*  *Polygonum macrophyllum*  *Polygonum milletii*  *Polygonum molle*  *Polygonum orientale*  *Polygonum perfoliatum*  *Polygonum polystachyum*  *Polygonum posumbu*  *Polygonum suffultoides*  *Polygonum viviparum*  *Rheum acuminatum*  *Rheum alexandrae*  *Rheum delavayi*  *Rheum kialense*  *Rheum likiangense*  *Rheum nobile*  *Rheum officinale*  *Rheum palmatum*  *Rheum tanguticum*  *Rumex acetosa*  *Rumex acetosella*  *Rumex amurensis*  *Rumex crispus*  *Rumex dentatus*  *Rumex japonicus*  *Rumex maritimus*  *Rumex nepalensis*  *Rumex patientia*  *Lysimachia candida*  *Lysimachia chenopodioides*  *Lysimachia chungdienensis*  *Lysimachia lobelioides*  *Lysimachia platypetala*  *Primula aurantiaca*  *Primula bathangensis*  *Aconitum hemsleyanum*  *Aconitum pulchellum*  *Anemone davidii*  *Anemone delavayi*  *Anemone demissa*  *Anemone dichotoma*  *Anemone flaccida*  *Anemone obtusiloba*  *Anemone rivularis*  *Batrachium bungei*  *Cimicifuga foetida*  *Cimicifuga yunnanensis*  *Clematis akebioides*  *Clematis apiifolia*  *Clematis brevicaudata*  *Clematis delavayi*  *Clematis gouriana*  *Clematis gracilifolia*  *Clematis lasiandra*  *Clematis macropetala*  *Clematis montana*  *Clematis orientalis*  *Clematis parviloba*  *Clematis peterae*  *Clematis pseudopogonandra*  *Clematis ranunculoides*  *Clematis rehderiana*  *Clematis tangutica*  *Delphinium grandiflorum*  *Pulsatilla millefolium*  *Ranunculus cantoniensis*  *Ranunculus chinensis*  *Souliea vaginata*  *Thalictrum aquilegiifolium*  *Thalictrum atriplex*  *Thalictrum cultratum*  *Thalictrum delavayi*  *Thalictrum finetii*  *Thalictrum foetidum*  *Thalictrum foliolosum*  *Thalictrum fortunei*  *Thalictrum javanicum*  *Thalictrum leuconotum*  *Thalictrum microgynum*  *Thalictrum minus*  *Thalictrum petaloideum*  *Thalictrum przewalskii*  *Thalictrum rostellatum*  *Thalictrum rutifolium*  *Thalictrum scabrifolium*  *Thalictrum squamiferum*  *Thalictrum uncatum*  *Thalictrum yunnanense*  *Berchemia floribunda*  *Berchemia yunnanensis*  *Hovenia acerba*  *Hovenia dulcis*  *Rhamnus dumetorum*  *Rhamnus gilgiana*  *Rhamnus leptophylla*  *Rhamnus tangutica*  *Rhamnus utilis*  *Rhamnus virgata*  *Sageretia horrida*  *Ziziphus montana*  *Agrimonia nipponica*  *Agrimonia pilosa*  *Amygdalus mira*  *Armeniaca sibirica*  *Armeniaca vulgaris*  *Cerasus cerasoides*  *Cerasus conadenia*  *Cerasus pusilliflora*  *Cerasus serrula*  *Cerasus serrulata*  *Coluria longifolia*  *Cotoneaster acuminatus*  *Cotoneaster acutifolius*  *Cotoneaster adpressus*  *Cotoneaster buxifolius*  *Cotoneaster coriaceus*  *Cotoneaster dammeri*  *Cotoneaster dielsianus*  *Cotoneaster divaricatus*  *Cotoneaster foveolatus*  *Cotoneaster franchetii*  *Cotoneaster harrysmithii*  *Cotoneaster hebephyllus*  *Cotoneaster horizontalis*  *Cotoneaster langei*  *Cotoneaster microphyllus*  *Cotoneaster moupinensis*  *Cotoneaster multiflorus*  *Cotoneaster pannosus*  *Cotoneaster rotundifolius*  *Cotoneaster rubens*  *Cotoneaster salicifolius*  *Cotoneaster subadpressus*  *Cotoneaster tenuipes*  *Crataegus chungtienensis*  *Crataegus cuneata*  *Dichotomanthes tristaniicarpa*  *Docynia delavayi*  *Duchesnea chrysantha*  *Fragaria gracilis*  *Fragaria vesca*  *Geum aleppicum*  *Geum japonicum*  *Kerria japonica*  *Malus hupehensis*  *Malus kansuensis*  *Malus rockii*  *Malus toringoides*  *Malus transitoria*  *Malus yunnanensis*  *Neillia serratisepala*  *Neillia thyrsiflora*  *Osteomeles schwerinae*  *Padus napaulensis*  *Padus perulata*  *Photinia glomerata*  *Photinia integrifolia*  *Photinia prionophylla*  *Photinia prunifolia*  *Photinia serratifolia*  *Potentilla bifurca*  *Potentilla chinensis*  *Potentilla coriandrifolia*  *Potentilla cuneata*  *Potentilla eriocarpa*  *Potentilla fallens*  *Potentilla glabra*  *Potentilla leuconota*  *Potentilla macrosepala*  *Potentilla multicaulis*  *Potentilla peduncularis*  *Potentilla saundersiana*  *Potentilla stenophylla*  *Potentilla tatsienluensis*  *Prunus mume*  *Pyracantha angustifolia*  *Pyracantha atalantioides*  *Pyracantha crenulata*  *Pyracantha fortuneana*  *Pyrus betulifolia*  *Pyrus pashia*  *Pyrus phaeocarpa*  *Rosa banksiopsis*  *Rosa brunonii*  *Rosa cymosa*  *Rosa duplicata*  *Rosa farreri*  *Rosa giraldii*  *Rosa glomerata*  *Rosa graciliflora*  *Rosa helenae*  *Rosa hugonis*  *Rosa longicuspis*  *Rosa macrophylla*  *Rosa mairei*  *Rosa moyesii*  *Rosa multibracteata*  *Rosa multiflora*  *Rosa odorata*  *Rosa omeiensis*  *Rosa prattii*  *Rosa roxburghii*  *Rosa sericea*  *Rosa sikangensis*  *Rosa soulieana*  *Rosa spinosissima*  *Rosa sweginzowii*  *Rosa webbiana*  *Rosa willmottiae*  *Rubus alexeterius*  *Rubus biflorus*  *Rubus buergeri*  *Rubus corchorifolius*  *Rubus delavayi*  *Rubus ellipticus*  *Rubus ichangensis*  *Rubus innominatus*  *Rubus lambertianus*  *Rubus lineatus*  *Rubus lutescens*  *Rubus mesogaeus*  *Rubus niveus*  *Rubus paniculatus*  *Rubus pentagonus*  *Rubus phoenicolasius*  *Rubus pileatus*  *Rubus pseudopileatus*  *Rubus subornatus*  *Sibbaldia cuneata*  *Sibbaldia procumbens*  *Sorbus alnifolia*  *Sorbus coronata*  *Sorbus corymbifera*  *Sorbus epidendron*  *Sorbus folgneri*  *Sorbus hemsleyi*  *Sorbus hupehensis*  *Sorbus insignis*  *Sorbus koehneana*  *Sorbus oligodonta*  *Sorbus pallescens*  *Sorbus pohuashanensis*  *Sorbus prattii*  *Sorbus reducta*  *Sorbus rufopilosa*  *Sorbus setschwanensis*  *Sorbus vilmorinii*  *Sorbus wallichii*  *Sorbus wilsoniana*  *Spenceria ramalana*  *Spiraea alpina*  *Spiraea bella*  *Spiraea calcicola*  *Spiraea canescens*  *Spiraea chinensis*  *Spiraea japonica*  *Spiraea mollifolia*  *Spiraea mongolica*  *Diplospora fruticosa*  *Galium bungei*  *Galium uliginosum*  *Hedyotis uncinella*  *Leptodermis pilosa*  *Rubia alata*  *Rubia cordifolia*  *Rubia podantha*  *Boenninghausenia albiflora*  *Skimmia arborescens*  *Zanthoxylum acanthopodium*  *Zanthoxylum myriacanthum*  *Meliosma cuneifolia*  *Salix myrtillacea*  *Koelreuteria paniculata*  *Sapindus delavayi*  *Bergenia purpurascens*  *Saxifraga litangensis*  *Tiarella polyphylla*  *Hemiphragma heterophyllum*  *Lagotis clarkei*  *Pedicularis superba*  *Ailanthus altissima*  *Datura stramonium*  *Nicandra physalodes*  *Solanum pseudocapsicum*  *Euscaphis japonica*  *Symplocos paniculata*  *Cryptomeria japonica*  *Tetracentron sinense*  *Anneslea fragrans*  *Eurya brevistyla*  *Eurya nitida*  *Schima argentea*  *Schima wallichii*  *Ternstroemia gymnanthera*  *Stellera chamaejasme*  *Trema angustifolia*  *Laportea bulbifera*  *Oreocnide integrifolia*  *Urtica hyperborea*  *Urtica laetevirens*  *Patrinia monandra*  *Callicarpa formosana*  *Callicarpa pseudorubella*  *Callicarpa rubella*  *Caryopteris forrestii*  *Caryopteris tangutica*  *Caryopteris trichosphaera*  *Clerodendrum bungei*  *Verbena officinalis*  *Vitex negundo*  *Ampelopsis acutidentata*  *Cayratia japonica*  *Cayratia pedata*  *Parthenocissus semicordata*  *Tetrastigma obtectum*  *Tetrastigma rumicispermum*  *Tetrastigma serrulatum*  *Tetrastigma yunnanense*  *Vitis betulifolia*  *Hedychium yunnanense*  *Zingiber officinale*  *Zingiber striolatum* | √  √  √  √  √  √  √  √  √  √  √  √  √  √  √  √  √  √  √  √  √  √  √  √  √  √  √  √  √  √  √  √  √  √  √  √  √  √  √  √  √  √  √  √  √  √  √  √  √  √  √  √  √  √  √  √  √  √  √  √  √  √  √  √  √  √  √  √  √  √  √  √  √  √  √  √  √  √  √  √  √  √  √  √  √  √  √  √  √  √  √  √  √  √  √  √  √  √  √  √  √  √  √  √  √  √  √  √  √  √  √  √  √  √  √  √  √  √  √  √  √  √  √  √  √  √  √  √  √  √  √  √  √  √  √  √  √  √  √  √  √  √  √  √  √  √  √  √  √  √  √  √  √  √  √  √  √  √  √  √  √  √  √  √  √  √  √  √  √  √  √  √  √  √  √  √  √  √  √  √  √  √  √  √  √  √  √  √  √  √  √  √  √  √  √  √  √  √  √  √  √  √  √  √  √  √  √  √  √  √  √  √  √  √  √  √  √  √  √  √  √  √  √  √  √  √  √  √  √  √  √  √  √  √  √  √  √  √  √  √  √  √  √  √  √  √  √  √  √  √  √  √  √  √  √  √  √  √  √  √  √  √  √  √  √  √  √  √  √  √  √  √  √  √  √  √  √  √  √  √  √  √  √  √  √  √  √  √  √  √  √  √  √  √  √  √  √  √  √  √  √  √  √  √  √  √  √  √  √  √  √  √  √  √  √  √  √  √  √  √  √  √  √  √  √  √  √  √  √  √  √  √  √  √  √  √  √  √  √  √  √  √  √  √  √  √  √  √  √  √  √  √  √  √  √  √  √  √  √  √  √  √  √  √  √  √  √  √  √  √  √  √  √  √  √  √  √  √  √  √  √  √  √  √  √  √  √  √  √  √  √  √  √  √  √  √  √  √  √  √  √  √  √  √  √  √  √  √  √  √  √  √  √  √  √  √  √  √  √  √  √  √  √  √  √  √  √  √  √  √  √  √  √  √  √  √  √  √  √  √  √  √  √  √  √  √  √  √  √  √  √  √  √  √  √  √  √  √  √  √  √  √  √  √  √  √  √  √  √  √  √  √  √  √  √  √  √  √  √  √  √  √  √  √  √  √  √  √  √  √  √  √  √  √  √  √  √  √  √  √  √  √  √  √  √  √  √  √  √  √  √  √  √  √  √  √  √  √  √  √  √  √  √  √  √  √  √  √  √  √  √  √  √  √  √  √  √  √  √  √  √  √  √  √  √  √  √  √  √  √  √  √  √  √  √  √  √  √  √  √  √  √  √  √  √  √  √  √  √  √  √  √  √  √  √  √  √  √  √  √  √  √  √  √ | √  √  √  √  √  √  √  √  √  √  √  √  √  √  √  √  √  √  √  √  √  √  √  √  √  √  √  √  √  √  √  √  √  √  √  √  √  √  √  √  √  √  √  √  √  √  √  √  √  √  √  √  √  √  √  √  √  √  √  √  √  √  √  √  √  √  √  √  √  √  √  √  √  √  √  √  √  √  √  √  √  √  √  √  √  √  √  √  √  √  √  √  √  √  √  √  √  √  √  √  √  √  √  √  √  √  √  √  √  √  √  √  √  √  √  √  √  √  √  √  √  √  √  √  √  √  √  √  √  √  √  √  √  √  √  √  √  √  √  √  √  √  √  √  √  √  √  √  √  √  √  √  √  √  √  √  √  √  √  √  √  √  √  √  √  √  √  √  √  √  √  √  √  √  √  √  √  √  √  √  √  √  √  √  √  √  √  √  √  √  √  √  √  √  √  √  √  √  √  √  √  √  √  √  √  √  √  √  √  √  √  √  √  √  √  √  √  √  √  √  √  √  √  √  √  √  √  √  √  √  √  √  √  √  √  √  √  √  √  √  √  √  √  √  √  √  √  √  √  √  √  √  √  √  √  √  √  √  √  √  √  √  √  √  √  √  √  √  √  √  √  √  √  √  √  √  √  √  √  √  √  √  √  √  √  √  √  √  √  √  √  √  √  √  √  √  √  √  √  √  √  √  √  √  √  √  √  √  √  √  √  √  √  √  √  √  √  √  √  √  √  √  √  √  √  √  √  √  √  √  √  √  √  √  √  √  √  √  √  √  √  √  √  √  √  √  √  √  √  √  √  √  √  √  √  √  √  √  √  √  √  √  √  √  √  √  √  √  √  √  √  √  √  √  √  √  √  √  √  √  √  √  √  √  √  √  √  √  √  √  √  √  √  √  √  √  √  √  √  √  √  √  √  √  √  √  √  √  √  √  √  √  √  √  √  √  √  √  √  √  √  √  √  √  √  √  √  √  √  √  √  √  √  √  √  √  √  √  √  √  √  √  √  √  √  √  √  √  √  √  √  √  √  √  √  √  √  √  √  √  √  √  √  √  √  √  √  √  √  √  √  √  √  √  √  √  √  √  √  √  √  √  √  √  √  √  √  √  √  √  √  √  √  √  √  √  √  √  √  √  √  √  √  √  √  √  √  √  √  √  √  √  √  √  √  √  √  √  √  √  √  √  √  √  √  √  √  √  √  √  √  √  √  √  √  √  √  √  √  √  √  √  √  √  √  √  √  √  √  √  √  √  √  √  √  √  √  √  √  √  √  √  √  √  √  √  √  √  √  √  √  √  √  √  √  √  √  √  √  √  √  √  √  √  √  √  √  √  √  √  √  √  √  √  √  √  √  √  √  √  √  √  √  √  √  √  √  √  √  √  √  √  √  √  √  √  √  √  √  √  √  √  √  √  √  √  √  √  √  √  √  √  √  √  √  √  √  √  √  √  √  √  √  √  √  √  √  √  √  √  √  √  √  √  √  √  √  √  √  √  √  √  √  √  √  √  √  √  √  √  √  √  √  √  √  √  √  √  √  √  √  √  √  √  √  √  √  √  √  √  √  √  √  √  √  √  √  √  √  √  √  √  √  √  √  √  √  √  √  √  √  √  √  √  √  √  √  √  √  √  √  √  √  √  √  √  √  √  √  √  √  √  √  √  √  √  √  √  √  √  √  √  √  √  √  √  √  √  √  √  √  √  √  √  √  √  √  √  √  √  √  √  √  √  √  √  √  √  √  √  √  √  √  √  √  √  √  √  √  √  √  √  √  √  √  √  √  √  √  √  √  √  √  √  √  √  √  √  √  √  √  √  √  √  √  √  √  √  √  √  √  √  √  √  √  √  √  √  √  √  √  √  √  √  √  √  √  √  √  √  √  √  √  √  √  √  √  √  √  √  √  √  √  √  √  √  √  √  √  √  √  √  √  √  √  √  √  √  √  √  √  √  √  √  √  √  √  √  √  √  √  √  √  √  √  √  √  √  √  √  √  √  √  √  √  √  √  √  √  √  √  √  √  √  √  √  √  √  √  √  √  √  √  √  √  √  √  √  √  √  √  √  √  √  √  √  √  √  √  √  √  √  √  √  √  √  √  √  √  √  √  √  √  √  √  √  √  √  √  √  √  √  √  √  √  √  √  √  √  √  √  √  √  √  √  √  √  √  √  √  √  √  √  √  √  √  √  √  √  √  √  √  √  √  √  √  √  √  √  √  √  √  √  √  √  √  √  √  √  √  √  √  √  √  √  √  √  √  √  √  √  √  √  √  √  √  √  √  √  √  √  √  √  √  √  √  √  √  √  √  √  √  √  √  √  √  √  √  √  √  √  √  √  √  √  √  √  √  √  √  √  √  √  √  √  √  √  √  √  √  √  √  √  √  √  √  √  √  √  √  √ | √  √  √  √  √  √  √  √  √  √  √  √  √  √  √  √  √  √  √  √  √  √  √  √  √  √  √  √  √  √  √  √  √  √  √  √  √  √  √  √  √  √  √  √  √  √  √  √  √  √  √  √  √  √  √  √  √  √  √  √  √  √  √  √  √  √  √  √  √  √  √  √  √  √  √  √  √  √  √  √  √  √  √  √  √  √  √  √  √  √  √  √  √  √  √  √  √  √  √  √  √  √  √  √  √  √  √  √  √  √  √  √  √  √  √  √  √  √  √  √  √  √  √  √  √  √  √  √  √  √  √  √  √  √  √  √  √  √  √  √  √  √  √  √  √  √  √  √  √  √  √  √  √  √  √  √  √  √  √  √  √  √  √  √  √  √  √  √  √  √  √  √  √  √  √  √  √  √  √  √  √  √  √  √  √  √  √  √  √  √  √  √  √  √  √  √  √  √  √  √  √  √  √  √  √  √  √  √  √  √  √  √  √  √  √  √  √  √  √  √  √  √  √  √  √  √  √  √  √  √  √  √  √  √  √  √  √  √  √  √  √  √  √  √  √  √  √  √  √  √  √  √  √  √  √  √  √  √  √  √  √  √  √  √  √  √  √  √  √  √  √  √  √  √  √  √  √  √  √  √  √  √  √  √  √  √  √  √  √  √  √  √  √  √  √  √  √  √  √  √  √  √  √  √  √  √  √  √  √  √  √  √  √  √  √  √  √  √  √  √  √  √  √  √  √  √  √  √  √  √  √  √  √  √  √  √  √  √  √  √  √  √  √  √  √  √  √  √  √  √  √  √  √  √  √  √  √  √  √  √  √  √  √  √  √  √  √  √  √  √  √  √  √  √  √  √  √  √  √  √  √  √  √  √  √  √  √  √  √  √  √  √  √  √  √  √  √  √  √  √  √  √  √  √  √  √  √  √  √  √  √  √  √  √  √  √  √  √  √  √  √  √  √  √  √  √  √  √  √  √  √  √  √  √  √  √  √  √  √  √  √  √  √  √  √  √  √  √  √  √  √  √  √  √  √  √  √  √  √  √  √  √  √  √  √  √  √  √  √  √  √  √  √  √  √  √  √  √  √  √  √  √  √  √  √  √  √  √  √  √  √  √  √  √  √  √  √  √  √  √  √  √  √  √  √  √  √  √  √  √  √  √  √  √  √  √  √  √  √  √  √  √  √  √  √  √  √  √  √  √  √  √  √  √  √  √  √  √  √  √  √  √  √  √  √  √  √  √  √  √  √  √  √  √  √  √  √  √  √  √  √  √  √  √  √  √  √  √  √  √  √  √  √  √  √  √  √  √  √  √  √  √  √  √  √  √  √  √  √  √  √  √  √  √  √  √  √  √  √  √  √  √  √  √  √  √  √  √  √  √  √  √  √  √  √  √  √  √  √  √  √  √  √  √  √  √  √  √  √  √  √  √  √  √  √  √  √  √  √  √  √  √  √  √  √  √  √  √  √  √  √  √  √  √  √  √  √  √  √  √  √  √  √  √  √  √  √  √  √  √  √  √  √  √  √  √  √  √  √  √  √  √  √  √  √  √  √  √  √  √  √  √  √  √  √  √  √  √  √  √  √  √  √  √  √  √  √  √  √  √  √  √  √  √  √  √  √  √  √  √  √  √  √  √  √  √  √  √  √  √  √  √  √  √  √  √  √  √  √  √  √  √  √  √  √  √  √  √  √  √  √  √  √  √  √  √  √  √  √  √  √  √  √  √  √  √  √  √  √  √  √  √  √  √  √  √  √  √  √  √  √  √  √  √  √  √  √  √  √  √  √  √  √  √  √  √  √  √  √  √  √  √  √  √  √  √  √  √  √  √  √  √  √  √  √  √  √  √  √  √  √  √  √  √  √  √  √  √  √  √  √  √  √  √  √  √  √  √  √  √  √  √  √  √  √  √  √  √  √  √  √  √  √  √  √  √  √  √  √  √  √  √  √  √  √  √  √  √  √  √  √  √  √  √  √  √  √  √  √  √  √  √  √  √  √  √  √  √  √  √  √  √  √  √  √  √  √  √  √  √  √  √  √  √  √  √  √  √  √  √  √  √  √  √  √  √  √  √  √  √  √  √  √  √  √  √  √  √  √  √  √  √  √  √  √  √  √  √  √  √  √  √  √  √  √  √  √  √  √  √  √  √  √  √  √ | √  √  √  √  √  √  √  √  √  √  √  √  √  √  √  √  √  √  √  √  √  √  √  √  √  √  √  √  √  √  √  √  √  √  √  √  √  √  √  √  √  √  √  √  √  √  √  √  √  √  √  √  √  √  √  √  √  √  √  √  √  √  √  √  √  √  √  √  √  √  √  √  √  √  √  √  √  √  √  √  √  √  √  √  √  √  √  √  √  √  √  √  √  √  √  √  √  √  √  √  √  √  √  √  √  √  √  √  √  √  √  √  √  √  √  √  √  √  √  √  √  √  √  √  √  √  √  √  √  √  √  √  √  √  √  √  √  √  √  √  √  √  √  √  √  √  √  √  √  √  √  √  √  √  √  √  √  √  √  √  √  √  √  √  √  √  √  √  √  √  √  √  √  √  √  √  √  √  √  √  √  √  √  √  √  √  √  √  √  √  √  √  √  √  √  √  √  √  √  √  √  √  √  √  √  √  √  √  √  √  √  √  √  √ | √  √  √  √  √  √  √  √  √  √  √  √  √  √  √  √  √  √  √  √  √  √  √  √  √  √  √  √  √  √  √  √  √  √  √  √  √  √  √  √  √  √  √  √  √  √  √  √  √  √  √  √  √  √  √  √  √  √  √  √  √  √  √  √  √ |

**TABLE S2** The details of sources of seeds per fruit for each species. (FOS-a represents using the record of seeds per fruit; FOS-b represents using the record of ovules per ovary; GBOWS represents the record from Germplasm Bank of Wild Species.)

| **SN** | **Species** | **Number** | **Source** | **Link** |
| --- | --- | --- | --- | --- |
| 1  2  3  4  5  6  7  8  9  10  11  12  13  14  15  16  17  18  19  20  21  22  23  24  25  26  27  28  29  30  31  32  33  34  35  36  37  38  39  40  41  42  43  44  45  46  47  48  49  50  51  52  53  54  55  56  57  58  59  60  61  62  63  64  65  66  67  68  69  70  71  72  73  74  75  76  77  78  79  80  81  82  83  84  85  86  87  88  89  90  91  92  93  94  95  96  97  98  99  100  101  102  103  104  105  106  107  108  109  110  111  112  113  114  115  116  117  118  119  120  121  122  123  124  125  126  127  128  129  130  131  132  133  134  135  136  137  138  139  140  141  142  143  144  145  146  147  148  149  150  151  152  153  154  155  156  157  158  159  160  161  162  163  164  165  166  167  168  169  170  171  172  173  174  175  176  177  178  179  180  181  182  183  184  185  186  187  188  189  190  191  192  193  194  195  196  197  198  199  200  201  202  203  204  205  206  207  208  209  210  211  212  213  214  215  216  217  218  219  220  221  222  223  224  225  226  227  228  229  230  231  232  233  234  235  236  237  238  239  240  241  242  243  244  245  246  247  248  249  250  251  252  253  254  255  256  257  258  259  260  261  262  263  264  265  266  267  268  269  270  271  272  273  274  275  276  277  278  279  280  281  282  283  284  285  286  287  288  289  290  291  292  293  294  295  296  297  298  299  300  301  302  303  304  305  306  307  308  309  310  311  312  313  314  315  316  317  318  319  320  321  322  323  324  325  326  327  328  329  330  331  332  333  334  335  336  337  338  339  340  341  342  343  344  345  346  347  348  349  350  351  352  353  354  355  356  357  358  359  360  361  362  363  364  365  366  367  368  369  370  371  372  373  374  375  376  377  378  379  380  381  382  383  384  385  386  387  388  389  390  391  392  393  394  395  396  397  398  399  400  401  402  403  404  405  406  407  408  409  410  411  412  413  414  415  416  417  418  419  420  421  422  423  424  425  426  427  428  429  430  431  432  433  434  435  436  437  438  439  440  441  442  443  444  445  446  447  448  449  450  451  452  453  454  455  456  457  458  459  460  461  462  463  464  465  466  467  468  469  470  471  472  473  474  475  476  477  478  479  480  481  482  483  484  485  486  487  488  489  490  491  492  493  494  495  496  497  498  499  500  501  502  503  504  505  506  507  508  509  510  511  512  513  514  515  516  517  518  519  520  521  522  523  524  525  526  527  528  529  530  531  532  533  534  535  536  537  538  539  540  541  542  543  544  545  546  547  548  549  550  551  552  553  554  555  556  557  558  559  560  561  562  563  564  565  566  567  568  569  570  571  572  573  574  575  576  577  578  579  580  581  582  583  584  585  586  587  588  589  590  591  592  593  594  595  596  597  598  599  600  601  602  603  604  605  606  607  608  609  610  611  612  613  614  615  616  617  618  619  620  621  622  623  624  625  626  627  628  629  630  631  632  633  634  635  636  637  638  639  640  641  642  643  644  645  646  647  648  649  650  651  652  653  654  655  656  657  658  659  660  661  662  663  664  665  666  667  668  669  670  671  672  673  674  675  676  677  678  679  680  681  682  683  684  685  586  687  688  689  690  691  692  693  694  695  696  697  698  699  700  701  702  703  704  705  706  707  708  709  710  711  712  713  714  715  716  717  718  719  720  721  722  723  724  725  726  727  728  729  730  731  732  733  734  735  736  737  738  739  740  741  742  743  744  745  746  747  748  749  750  751  752  753  754  755  756  757  758  759  760  761  762  763  764  765  766  767  768  769  770  771  772  773  774  775  776  777  778  779  780  781  782  783  784  785  786  787  788  789  790  791  792  793  794  795  796  797  798  799  800  801  802  803  804  805  806  807  808  809  810  811  812  813  814  815  816  817  818  819  820  821  822  823  824  825  826  827  828  829  830  831  832  833  834  835  836  837  838  839  840  841  842  843  844  845  846  847  848  849  850  851  852  853  854  855  856  857  858  859  860  861  862  863  864  865  866  867  868  869  870  871  872  873  874  875  876  877  878  879  880  881  882  883  884  885  886  887  888  889  890  891  892  893  894  895  896  897  898  899  900  901  902  903  904  905  906  907  908  909  910  911  912  913  914  915  916  917  918  919  920  921  922  923  924  925  926  927  928  929  930  931  932  933  934  935  936  937  938  939  940  941  942  943  944  945  946  947  948  949  950  951  952  953  954  955  956  957  958  959  960  961  962  963  964  965  966  967  968  969  970  971  972  973  974  975  976  977  978  979  980  981  982  983  984  985  986  987  988  989  990  991  992  993  994  995  996  997  998  999  1000  1001  1002  1003  1004  1005  1006  1007  1008  1009  1010  1011  1012  1013  1014  1015  1016  1017  1018  1019  1020  1021  1022  1023  1024  1025  1026  1027  1028  1029  1030  1031  1032  1033  1034  1035  1036  1037  1038  1039  1040  1041  1042  1043  1044  1045  1046  1047  1048  1049  1050  1051  1052  1053  1054  1055  1056  1057  1058  1059  1060  1061  1062  1063  1064  1065  1066  1067  1068  1069  1070  1071  1072  1073  1074  1075  1076  1077  1078  1079  1080  1081  1082  1083  1084  1085  1086  1087  1088  1089  1090  1091  1092  1093  1094  1095  1096  1097  1098  1099  1100  1101  1102  1103  1104  1105  1106  1107  1108  1109  1110  1111  1112  1113  1114  1115  1116  1117  1118  1119 | *Acer amplum*  *Acer caesium*  *Acer cappadocicum*  *Acer caudatum*  *Acer davidii*  *Acer flabellatum*  *Acer kuomeii*  *Acer laxiflorum*  *Acer palmatum*  *Acer sinense*  *Acer stachyophyllum*  *Sambucus adnata*  *Sambucus williamsii*  *Viburnum betulifolium*  *Viburnum chinshanense*  *Viburnum congestum*  *Viburnum cylindricum*  *Viburnum dilatatum*  *Viburnum erubescens*  *Viburnum foetidum*  *Viburnum glomeratum*  *Viburnum hupehense*  *Viburnum punctatum*  *Viburnum utile*  *Achyranthes bidentata*  *Achyranthes longifolia*  *Amaranthus blitum*  *Amaranthus caudatus*  *Amaranthus spinosus*  *Amaranthus tricolor*  *Celosia argentea*  *Cyathula capitata*  *Cyathula officinalis*  *Deeringia amaranthoides*  *Choerospondias axillaris*  *Pistacia chinensis*  *Pistacia weinmanniifolia*  *Rhus chinensis*  *Rhus potaninii*  *Rhus punjabensis*  *Toxicodendron grandiflorum*  *Toxicodendron succedaneum*  *Toxicodendron vernicifluum*  *Toxicodendron wallichii*  *Polyalthia cerasoides*  *Acronema astrantiifolium*  *Acronema schneideri*  *Angelica apaensis*  *Angelica decursiva*  *Angelica laxifoliata*  *Angelica omeiensis*  *Angelica oncosepala*  *Angelica pseudoselinum*  *Angelica sinensis*  *Anthriscus sylvestris*  *Bupleurum candollei*  *Bupleurum commelynoideum*  *Bupleurum hamiltonii*  *Bupleurum marginatum*  *Bupleurum triradiatum*  *Bupleurum yunnanense*  *Carum buriaticum*  *Carum carvi*  *Centella asiatica*  *Chamaesium paradoxum*  *Coriandrum sativum*  *Cyclorhiza waltonii*  *Daucus carota*  *Ferula kingdon-wardii*  *Foeniculum vulgare*  *Heracleum acuminatum*  *Heracleum bivittatum*  *Heracleum candicans*  *Heracleum millefolium*  *Heracleum moellendorffii*  *Heracleum nyalamense*  *Heracleum scabridum*  *Ligusticum acuminatum*  *Ligusticum angelicifolium*  *Ligusticum brachylobum*  *Ligusticum daucoides*  *Ligusticum oliverianum*  *Ligusticum pteridophyllum*  *Ligusticum scapiforme*  *Ligusticum sikiangense*  *Ligusticum thomsonii*  *Notopterygium incisum*  *Oenanthe hookeri*  *Oenanthe javanica*  *Oenanthe sinensis*  *Peucedanum praeruptorum*  *Peucedanum rubricaule*  *Peucedanum turgeniifolium*  *Peucedanum violaceum*  *Physospermopsis muliensis*  *Pimpinella bisinuata*  *Pimpinella candolleana*  *Pimpinella diversifolia*  *Pimpinella rubescens*  *Pimpinella smithii*  *Pleurospermum amabile*  *Pleurospermum angelicoides*  *Pleurospermum aromaticum*  *Pleurospermum franchetianum*  *Pleurospermum hookeri*  *Pleurospermum linearilobum*  *Pleurospermum nanum*  *Pleurospermum pulszkyi*  *Pleurospermum wilsonii*  *Pleurospermum wrightianum*  *Pternopetalum davidii*  *Selinum cryptotaenium*  *Sinolimprichtia alpina*  *Sphallerocarpus gracilis*  *Tongoloa silaifolia*  *Torilis japonica*  *Torilis scabra*  *Vicatia thibetica*  *Ilex polyneura*  *Ilex wattii*  *Arisaema erubescens*  *Arisaema flavum*  *Arisaema heterophyllum*  *Arisaema tortuosum*  *Aralia caesia*  *Aralia chinensis*  *Aralia decaisneana*  *Aralia echinocaulis*  *Brassaiopsis glomerulata*  *Eleutherococcus giraldii*  *Schefflera minutistellata*  *Adenocaulon himalaicum*  *Ainsliaea latifolia*  *Ainsliaea spicata*  *Anaphalis chungtienensis*  *Arctium lappa*  *Arctium tomentosum*  *Aster auriculatus*  *Aster batangensis*  *Aster diplostephioides*  *Aster dolichopodus*  *Aster flaccidus*  *Aster handelii*  *Aster oreophilus*  *Aster souliei*  *Aster tataricus*  *Aster tongolensis*  *Aster yunnanensis*  *Bidens biternata*  *Bidens parviflora*  *Bidens pilosa*  *Bidens tripartita*  *Carduus acanthoides*  *Carduus crispus*  *Carduus nutans*  *Carpesium cernuum*  *Carpesium divaricatum*  *Carpesium lipskyi*  *Carpesium nepalense*  *Carpesium scapiforme*  *Carpesium triste*  *Chaetoseris dolichophylla*  *Cirsium arvense*  *Cirsium eriophoroides*  *Cirsium henryi*  *Cirsium japonicum*  *Cirsium leo*  *Cirsium periacanthaceum*  *Cirsium shansiense*  *Cirsium souliei*  *Cremanthodium brachychaetum*  *Cremanthodium calcicola*  *Cremanthodium campanulatum*  *Cremanthodium citriflorum*  *Cremanthodium ellisii*  *Cremanthodium helianthus*  *Cremanthodium lineare*  *Cremanthodium principis*  *Cremanthodium reniforme*  *Cremanthodium stenactinium*  *Cremanthodium suave*  *Dolomiaea calophylla*  *Dolomiaea souliei*  *Doronicum oblongifolium*  *Doronicum stenoglossum*  *Dubyaea amoena*  *Erigeron breviscapus*  *Eupatorium japonicum*  *Gerbera nivea*  *Gerbera piloselloides*  *Gynura cusimbua*  *Hieracium umbellatum*  *Inula racemosa*  *Leibnitzia anandria*  *Leibnitzia pusilla*  *Leibnitzia ruficoma*  *Ligularia achyrotricha*  *Ligularia alatipes*  *Ligularia atroviolacea*  *Ligularia brassicoides*  *Ligularia confertiflora*  *Ligularia cremanthodioides*  *Ligularia cyathiceps*  *Ligularia cymbulifera*  *Ligularia dictyoneura*  *Ligularia duciformis*  *Ligularia fangiana*  *Ligularia fischeri*  *Ligularia hodgsonii*  *Ligularia hookeri*  *Ligularia intermedia*  *Ligularia kanaitzensis*  *Ligularia konkalingensis*  *Ligularia lamarum*  *Ligularia lankongensis*  *Ligularia lapathifolia*  *Ligularia latihastata*  *Ligularia liatroides*  *Ligularia longihastata*  *Ligularia melanocephala*  *Ligularia muliensis*  *Ligularia myriocephala*  *Ligularia nelumbifolia*  *Ligularia odontomanes*  *Ligularia pleurocaulis*  *Ligularia potaninii*  *Ligularia przewalskii*  *Ligularia purdomii*  *Ligularia rumicifolia*  *Ligularia sagitta*  *Ligularia sibirica*  *Ligularia stenoglossa*  *Ligularia subspicata*  *Ligularia tenuicaulis*  *Ligularia tongolensis*  *Ligularia tsangchanensis*  *Ligularia veitchiana*  *Ligularia vellerea*  *Ligularia virgaurea*  *Ligularia yunnanensis*  *Olgaea tangutica*  *Parasenecio latipes*  *Parasenecio palmatisectus*  *Parasenecio roborowskii*  *Pertya berberidoides*  *Pertya phylicoides*  *Picris hieracioides*  *Picris japonica*  *Saussurea arenaria*  *Saussurea caudata*  *Saussurea cochlearifolia*  *Saussurea columnaris*  *Saussurea dzeurensis*  *Saussurea epilobioides*  *Saussurea erubescens*  *Saussurea globosa*  *Saussurea graminea*  *Saussurea graminifolia*  *Saussurea gyacaensis*  *Saussurea hieracioides*  *Saussurea integrifolia*  *Saussurea iodostegia*  *Saussurea japonica*  *Saussurea katochaete*  *Saussurea laniceps*  *Saussurea leontodontoides*  *Saussurea leucoma*  *Saussurea likiangensis*  *Saussurea longifolia*  *Saussurea loriformis*  *Saussurea macrota*  *Saussurea neofranchetii*  *Saussurea nigrescens*  *Saussurea nyalamensis*  *Saussurea obvallata*  *Saussurea pachyneura*  *Saussurea phaeantha*  *Saussurea pinetorum*  *Saussurea pinnatidentata*  *Saussurea polycolea*  *Saussurea poochlamys*  *Saussurea populifolia*  *Saussurea przewalskii*  *Saussurea retroserrata*  *Saussurea romuleifolia*  *Saussurea scabrida*  *Saussurea semifasciata*  *Saussurea semilyrata*  *Saussurea stella*  *Saussurea stoliczkae*  *Saussurea stricta*  *Saussurea subulata*  *Saussurea subulisquama*  *Saussurea sylvatica*  *Saussurea tatsienensis*  *Saussurea vestita*  *Saussurea yunnanensis*  *Senecio megalanthus*  *Senecio muliensis*  *Soroseris erysimoides*  *Syncalathium souliei*  *Synotis erythropappa*  *Synurus deltoides*  *Taraxacum borealisinense*  *Taraxacum chionophilum*  *Taraxacum dasypodum*  *Taraxacum lanigerum*  *Taraxacum leucanthum*  *Taraxacum lugubre*  *Youngia paleacea*  *Impatiens lateristachys*  *Berberis agricola*  *Berberis amoena*  *Berberis approximata*  *Berberis concinna*  *Berberis davidii*  *Berberis dictyoneura*  *Berberis dictyophylla*  *Berberis ferdinandi coburgii*  *Berberis franchetiana*  *Berberis gyalaica*  *Berberis henryana*  *Berberis ignorata*  *Berberis insignis*  *Berberis jamesiana*  *Berberis julianae*  *Berberis kansuensis*  *Berberis lecomtei*  *Berberis metapolyantha*  *Berberis minutiflora*  *Berberis muliensis*  *Berberis obovatifolia*  *Berberis papillifera*  *Berberis pruinosa*  *Berberis reticulinervis*  *Berberis sublevis*  *Berberis tsarongensis*  *Berberis wilsonae*  *Berberis yunnanensis*  *Mahonia fortunei*  *Nandina domestica*  *Alnus cremastogyne*  *Alnus nepalensis*  *Betula platyphylla*  *Betula potaninii*  *Catalpa ovata*  *Incarvillea arguta*  *Incarvillea lutea*  *Incarvillea mairei*  *Antiotrema dunnianum*  *Cynoglossum amabile*  *Cynoglossum lanceolatum*  *Microula floribunda*  *Microula forrestii*  *Microula sikkimensis*  *Onosma exsertum*  *Onosma multiramosum*  *Onosma paniculatum*  *Onosma sinicum*  *Onosma waddellii*  *Arabis hirsuta*  *Capsella bursa pastoris*  *Cardamine impatiens*  *Cardamine macrophylla*  *Cardamine scutata*  *Cardamine tangutorum*  *Cardamine yunnanensis*  *Megacarpaea delavayi*  *Megacarpaea polyandra*  *Thlaspi arvense*  *Sarcococca hookeriana*  *Calycanthus floridus*  *Adenophora khasiana*  *Campanula chinensis*  *Campanula pallida*  *Codonopsis convolvulacea*  *Codonopsis foetens*  *Codonopsis gombalana*  *Codonopsis purpurea*  *Cyananthus delavayi*  *Cyananthus formosus*  *Cyananthus hookeri*  *Cyananthus incanus*  *Cyananthus inflatus*  *Cyananthus longiflorus*  *Cyananthus macrocalyx*  *Cyananthus microphyllus*  *Cannabis sativa*  *Lonicera caerulea*  *Lonicera cyanocarpa*  *Lonicera ligustrina*  *Lonicera maackii*  *Arenaria yunnanensis*  *Silene himalayensis*  *Stellaria yunnanensis*  *Celastrus angulatus*  *Celastrus gemmatus*  *Celastrus rosthornianus*  *Euonymus grandiflorus*  *Euonymus nanoides*  *Acroglochin persicarioides*  *Chenopodium album*  *Chenopodium ficifolium*  *Chenopodium glaucum*  *Chenopodium hybridum*  *Microgynoecium tibeticum*  *Circaeaster agrestis*  *Hypericum monogynum*  *Commelina paludosa*  *Cuscuta chinensis*  *Cuscuta europaea*  *Dinetus racemosus*  *Ipomoea purpurea*  *Merremia hederacea*  *Coriaria nepalensis*  *Coriaria terminalis*  *Cornus capitata*  *Cornus hemsleyi*  *Cornus macrophylla*  *Cornus oblonga*  *Platycladus orientalis*  *Blysmus sinocompressus*  *Carex atrata*  *Carex baccans*  *Carex composita*  *Carex cruciata*  *Carex filicina*  *Carex haematostoma*  *Carex hirtelloides*  *Carex kansuensis*  *Carex laeta*  *Carex lehmannii*  *Carex schneideri*  *Eleocharis yokoscensis*  *Fimbristylis ovata*  *Kobresia cuneata*  *Kobresia kansuensis*  *Kobresia setschwanensis*  *Schoenoplectus tabernaemontani*  *Dioscorea bulbifera*  *Dioscorea collettii*  *Dioscorea hemsleyi*  *Dioscorea kamoonensis*  *Dipsacus asper*  *Dipsacus atratus*  *Dipsacus chinensis*  *Dipsacus inermis*  *Dipsacus japonicus*  *Pterocephalus bretschneideri*  *Pterocephalus hookeri*  *Diospyros lotus*  *Elaeagnus delavayi*  *Elaeagnus lanceolata*  *Elaeagnus umbellata*  *Hippophae neurocarpa*  *Hippophae rhamnoides*  *Hippophae tibetana*  *Ephedra gerardiana*  *Ephedra likiangensis*  *Ephedra minuta*  *Rhododendron decorum*  *Rhododendron yunnanense*  *Eucommia ulmoides*  *Euphorbia esula*  *Euphorbia fischeriana*  *Euphorbia griffithii*  *Euphorbia jolkinii*  *Euphorbia micractina*  *Euphorbia stracheyi*  *Excoecaria acerifolia*  *Macaranga indica*  *Phyllanthus cochinchinensis*  *Phyllanthus emblica*  *Triadica sebifera*  *Euptelea pleiosperma*  *Acacia pennata*  *Apios carnea*  *Astragalus adsurgens*  *Astragalus degensis*  *Astragalus ernestii*  *Astragalus floridus*  *Astragalus monbeigii*  *Astragalus strictus*  *Astragalus tongolensis*  *Bauhinia brachycarpa*  *Cajanus cajan*  *Crotalaria pallida*  *Crotalaria sessiliflora*  *Dalbergia mimosoides*  *Dendrolobium triangulare*  *Desmodium elegans*  *Desmodium heterocarpon*  *Desmodium multiflorum*  *Desmodium sequax*  *Desmodium styracifolium*  *Desmodium yunnanense*  *Flemingia latifolia*  *Hedysarum citrinum*  *Hedysarum tanguticum*  *Indigofera amblyantha*  *Indigofera balfouriana*  *Indigofera delavayi*  *Indigofera lenticellata*  *Indigofera nigrescens*  *Indigofera pendula*  *Kummerowia striata*  *Lespedeza bicolor*  *Lespedeza pilosa*  *Leucaena leucocephala*  *Lotus corniculatus*  *Medicago edgeworthii*  *Medicago lupulina*  *Medicago minima*  *Melilotus indicus*  *Piptanthus nepalensis*  *Rhynchosia minima*  *Robinia pseudoacacia*  *Salweenia wardii*  *Senna occidentalis*  *Senna tora*  *Sophora davidii*  *Sophora velutina*  *Thermopsis barbata*  *Thermopsis lanceolata*  *Tibetia himalaica*  *Trifolium repens*  *Vicia bungei*  *Vicia cracca*  *Vicia unijuga*  *Vigna vexillata*  *Gentiana atuntsiensis*  *Gentiana macrophylla*  *Gentianopsis paludosa*  *Swertia macrosperma*  *Veratrilla baillonii*  *Geranium erianthum*  *Geranium nepalense*  *Geranium pseudosibiricum*  *Geranium strictipes*  *Geranium wilfordii*  *Iris bulleyana*  *Iris chrysographes*  *Iris clarkei*  *Iris delavayi*  *Iris goniocarpa*  *Iris japonica*  *Iris lactea*  *Iris ruthenica*  *Iris subdichotoma*  *Triglochin palustris*  *Ajuga lupulina*  *Albizia bracteata*  *Albizia kalkora*  *Albizia lucidior*  *Anisomeles indica*  *Chelonopsis souliei*  *Clinopodium chinense*  *Clinopodium confine*  *Clinopodium gracile*  *Clinopodium megalanthum*  *Clinopodium polycephalum*  *Clinopodium repens*  *Clinopodium urticifolium*  *Craniotome furcata*  *Dracocephalum calophyllum*  *Dracocephalum heterophyllum*  *Dracocephalum propinquum*  *Dracocephalum tanguticum*  *Elsholtzia bodinieri*  *Elsholtzia capituligera*  *Elsholtzia ciliata*  *Elsholtzia cyprianii*  *Elsholtzia densa*  *Elsholtzia eriostachya*  *Elsholtzia flava*  *Elsholtzia fruticosa*  *Elsholtzia pilosa*  *Elsholtzia souliei*  *Elsholtzia strobilifera*  *Eriophyton wallichii*  *Galeopsis bifida*  *Geniosporum coloratum*  *Isodon bulleyanus*  *Isodon coetsa*  *Isodon hispidus*  *Isodon japonicus*  *Isodon lophanthoides*  *Isodon pharicus*  *Isodon sculponeatus*  *Lagopsis supina*  *Lamiophlomis rotata*  *Lamium barbatum*  *Leonurus japonicus*  *Leonurus sibiricus*  *Leucas ciliata*  *Melissa axillaris*  *Microtoena delavayi*  *Mosla cavaleriei*  *Nepeta cataria*  *Nepeta laevigata*  *Nepeta prattii*  *Nepeta sibirica*  *Nepeta souliei*  *Nepeta stewartiana*  *Nepeta tenuiflora*  *Notochaete hamosa*  *Ocimum gratissimum*  *Origanum vulgare*  *Perilla frutescens*  *Phlomis agraria*  *Phlomis atropurpurea*  *Phlomis medicinalis*  *Phlomis setifera*  *Phlomis strigosa*  *Phlomis tibetica*  *Phlomis younghushandii*  *Pogostemon brevicorollus*  *Pogostemon glaber*  *Pogostemon nigrescens*  *Prunella hispida*  *Prunella vulgaris*  *Salvia castanea*  *salvia digitaloides*  *Salvia flava*  *Salvia japonica*  *Salvia prattii*  *Salvia przewalskii*  *Salvia roborowskii*  *Salvia smithii*  *Salvia subpalmatinervis*  *Salvia trijuga*  *Salvia yunnanensis*  *Scutellaria chungtienensis*  *Allium beesianum*  *Allium carolinianum*  *Allium cyaneum*  *Allium cyathophorum*  *Allium fasciculatum*  *Allium forrestii*  *Allium hookeri*  *Allium macranthum*  *Allium mairei*  *Allium ovalifolium*  *Allium prattii*  *Allium przewalskianum*  *Allium ramosum*  *Allium rude*  *Allium sikkimense*  *Allium tanguticum*  *Allium tuberosum*  *Allium wallichii*  *Asparagus filicinus*  *Asparagus meioclados*  *Asparagus setaceus*  *Eremurus chinensis*  *Lilium lophophorum*  *Polygonatum cirrhifolium*  *Polygonatum verticillatum*  *Manglietia insignis*  *Aspidopterys glabriuscula*  *Abutilon indicum*  *Abutilon paniculatum*  *Abutilon theophrasti*  *Hibiscus trionum*  *Kydia calycina*  *Malva verticillata*  *Triumfetta cana*  *Triumfetta pilosa*  *Triumfetta rhomboidea*  *Urena lobata*  *Sinomenium acutum*  *Stephania hernandiifolia*  *Broussonetia papyrifera*  *Ficus chapaensis*  *Acanthocalyx alba*  *Morina chinensis*  *Morina kokonorica*  *Ardisia virens*  *Embelia floribunda*  *Embelia ribes*  *Myrsine africana*  *Myrsine semiserrata*  *Oxybaphus himalaicus*  *Camptotheca acuminata*  *Nyssa sinensis*  *Fraxinus chinensis*  *Fraxinus sikkimensis*  *Jasminum humile*  *Jasminum officinale*  *Jasminum subhumile*  *Ligustrum compactum*  *Ligustrum confusum*  *Ligustrum delavayanum*  *Ligustrum lucidum*  *Ligustrum quihoui*  *Ligustrum sempervirens*  *Syringa yunnanensis*  *Adenia cardiophylla*  *Phryma leptostachya*  *Phytolacca acinosa*  *Phytolacca americana*  *Abies squamata*  *Picea asperata*  *Picea likiangensis*  *Picea purpurea*  *Pinus densata*  *Piper mullesua*  *Piper thomsonii*  *Pittosporum brevicalyx*  *Pittosporum crispulum*  *Pittosporum heterophyllum*  *Plantago asiatica*  *Plantago cavaleriei*  *Plantago depressa*  *Plantago gentianoides*  *Plantago major*  *Plumbagella micrantha*  *Achnatherum inebrians*  *Achnatherum sibiricum*  *Aristida triseta*  *Arundinella hirta*  *Avena chinensis*  *Avena fatua*  *Avena sativa*  *Beckmannia syzigachne*  *Bothriochloa ischaemum*  *Brachypodium pratense*  *Brachypodium sylvaticum*  *Bromus japonicus*  *Bromus plurinodis*  *Bromus porphyranthos*  *Bromus remotiflorus*  *Bromus sinensis*  *Bromus tectorum*  *Capillipedium assimile*  *Capillipedium parviflorum*  *Cymbopogon distans*  *Cymbopogon goeringii*  *Dactylis glomerata*  *Deyeuxia flavens*  *Deyeuxia pulchella*  *Digitaria cruciata*  *Digitaria sanguinalis*  *Echinochloa colona*  *Echinochloa crusgalli*  *Echinochloa frumentacea*  *Elymus antiquus*  *Elymus dahuricus*  *Elymus excelsus*  *Elymus nutans*  *Elymus sibiricus*  *Eragrostis atrovirens*  *Eulalia speciosa*  *Festuca gigantea*  *Festuca leptopogon*  *Festuca rubra*  *Festuca vierhapperi*  *Festuca yunnanensis*  *Heteropogon contortus*  *Ischaemum barbatum*  *Lolium perenne*  *Lophatherum gracile*  *Pennisetum alopecuroides*  *Pennisetum flaccidum*  *Phaenosperma globosa*  *Piptatherum munroi*  *Poa alpina*  *Poa perennis*  *Polypogon fugax*  *Ptilagrostis dichotoma*  *Schizachyrium delavayi*  *Setaria pumila*  *Setaria viridis*  *Setaria yunnanensis*  *Stipa capillacea*  *Stipa penicillata*  *Stipa purpurea*  *Stipa regeliana*  *Stipa roborowskyi*  *Antenoron filiforme*  *Fagopyrum esculentum*  *Fagopyrum gracilipes*  *Fallopia aubertii*  *Fallopia dumetorum*  *Fallopia multiflora*  *Oxyria digyna*  *Oxyria sinensis*  *Polygonum amphibium*  *Polygonum aviculare*  *Polygonum capitatum*  *Polygonum cathayanum*  *Polygonum chinense*  *Polygonum forrestii*  *Polygonum glaciale*  *Polygonum griffithii*  *Polygonum hydropiper*  *Polygonum jucundum*  *Polygonum lapathifolium*  *Polygonum macrophyllum*  *Polygonum milletii*  *Polygonum molle*  *Polygonum orientale*  *Polygonum perfoliatum*  *Polygonum polystachyum*  *Polygonum posumbu*  *Polygonum suffultoides*  *Polygonum viviparum*  *Rheum acuminatum*  *Rheum alexandrae*  *Rheum delavayi*  *Rheum kialense*  *Rheum likiangense*  *Rheum nobile*  *Rheum officinale*  *Rheum palmatum*  *Rheum tanguticum*  *Rumex acetosa*  *Rumex acetosella*  *Rumex amurensis*  *Rumex crispus*  *Rumex dentatus*  *Rumex japonicus*  *Rumex maritimus*  *Rumex nepalensis*  *Rumex patientia*  *Lysimachia candida*  *Lysimachia chenopodioides*  *Lysimachia chungdienensis*  *Lysimachia lobelioides*  *Lysimachia platypetala*  *Primula aurantiaca*  *Primula bathangensis*  *Aconitum hemsleyanum*  *Aconitum pulchellum*  *Anemone davidii*  *Anemone delavayi*  *Anemone demissa*  *Anemone dichotoma*  *Anemone flaccida*  *Anemone obtusiloba*  *Anemone rivularis*  *Batrachium bungei*  *Cimicifuga foetida*  *Cimicifuga yunnanensis*  *Clematis akebioides*  *Clematis apiifolia*  *Clematis brevicaudata*  *Clematis delavayi*  *Clematis gouriana*  *Clematis gracilifolia*  *Clematis lasiandra*  *Clematis macropetala*  *Clematis montana*  *Clematis orientalis*  *Clematis parviloba*  *Clematis peterae*  *Clematis pseudopogonandra*  *Clematis ranunculoides*  *Clematis rehderiana*  *Clematis tangutica*  *Delphinium grandiflorum*  *Pulsatilla millefolium*  *Ranunculus cantoniensis*  *Ranunculus chinensis*  *Souliea vaginata*  *Thalictrum aquilegiifolium*  *Thalictrum atriplex*  *Thalictrum cultratum*  *Thalictrum delavayi*  *Thalictrum finetii*  *Thalictrum foetidum*  *Thalictrum foliolosum*  *Thalictrum fortunei*  *Thalictrum javanicum*  *Thalictrum leuconotum*  *Thalictrum microgynum*  *Thalictrum minus*  *Thalictrum petaloideum*  *Thalictrum przewalskii*  *Thalictrum rostellatum*  *Thalictrum rutifolium*  *Thalictrum scabrifolium*  *Thalictrum squamiferum*  *Thalictrum uncatum*  *Thalictrum yunnanense*  *Berchemia floribunda*  *Berchemia yunnanensis*  *Hovenia acerba*  *Hovenia dulcis*  *Rhamnus dumetorum*  *Rhamnus gilgiana*  *Rhamnus leptophylla*  *Rhamnus tangutica*  *Rhamnus utilis*  *Rhamnus virgata*  *Sageretia horrida*  *Ziziphus montana*  *Agrimonia nipponica*  *Agrimonia pilosa*  *Amygdalus mira*  *Armeniaca sibirica*  *Armeniaca vulgaris*  *Cerasus cerasoides*  *Cerasus conadenia*  *Cerasus pusilliflora*  *Cerasus serrula*  *Cerasus serrulata*  *Coluria longifolia*  *Cotoneaster acuminatus*  *Cotoneaster acutifolius*  *Cotoneaster adpressus*  *Cotoneaster buxifolius*  *Cotoneaster coriaceus*  *Cotoneaster dammeri*  *Cotoneaster dielsianus*  *Cotoneaster divaricatus*  *Cotoneaster foveolatus*  *Cotoneaster franchetii*  *Cotoneaster harrysmithii*  *Cotoneaster hebephyllus*  *Cotoneaster horizontalis*  *Cotoneaster langei*  *Cotoneaster microphyllus*  *Cotoneaster moupinensis*  *Cotoneaster multiflorus*  *Cotoneaster pannosus*  *Cotoneaster rotundifolius*  *Cotoneaster rubens*  *Cotoneaster salicifolius*  *Cotoneaster subadpressus*  *Cotoneaster tenuipes*  *Crataegus chungtienensis*  *Crataegus cuneata*  *Dichotomanthes tristaniicarpa*  *Docynia delavayi*  *Duchesnea chrysantha*  *Fragaria gracilis*  *Fragaria vesca*  *Geum aleppicum*  *Geum japonicum*  *Kerria japonica*  *Malus hupehensis*  *Malus kansuensis*  *Malus rockii*  *Malus toringoides*  *Malus transitoria*  *Malus yunnanensis*  *Neillia serratisepala*  *Neillia thyrsiflora*  *Osteomeles schwerinae*  *Padus napaulensis*  *Padus perulata*  *Photinia glomerata*  *Photinia integrifolia*  *Photinia prionophylla*  *Photinia prunifolia*  *Photinia serratifolia*  *Potentilla bifurca*  *Potentilla chinensis*  *Potentilla coriandrifolia*  *Potentilla cuneata*  *Potentilla eriocarpa*  *Potentilla fallens*  *Potentilla glabra*  *Potentilla leuconota*  *Potentilla macrosepala*  *Potentilla multicaulis*  *Potentilla peduncularis*  *Potentilla saundersiana*  *Potentilla stenophylla*  *Potentilla tatsienluensis*  *Prunus mume*  *Pyracantha angustifolia*  *Pyracantha atalantioides*  *Pyracantha crenulata*  *Pyracantha fortuneana*  *Pyrus betulifolia*  *Pyrus pashia*  *Pyrus phaeocarpa*  *Rosa banksiopsis*  *Rosa brunonii*  *Rosa cymosa*  *Rosa duplicata*  *Rosa farreri*  *Rosa giraldii*  *Rosa glomerata*  *Rosa graciliflora*  *Rosa helenae*  *Rosa hugonis*  *Rosa longicuspis*  *Rosa macrophylla*  *Rosa mairei*  *Rosa moyesii*  *Rosa multibracteata*  *Rosa multiflora*  *Rosa odorata*  *Rosa omeiensis*  *Rosa prattii*  *Rosa roxburghii*  *Rosa sericea*  *Rosa sikangensis*  *Rosa soulieana*  *Rosa spinosissima*  *Rosa sweginzowii*  *Rosa webbiana*  *Rosa willmottiae*  *Rubus alexeterius*  *Rubus biflorus*  *Rubus buergeri*  *Rubus corchorifolius*  *Rubus delavayi*  *Rubus ellipticus*  *Rubus ichangensis*  *Rubus innominatus*  *Rubus lambertianus*  *Rubus lineatus*  *Rubus lutescens*  *Rubus mesogaeus*  *Rubus niveus*  *Rubus paniculatus*  *Rubus pentagonus*  *Rubus phoenicolasius*  *Rubus pileatus*  *Rubus pseudopileatus*  *Rubus subornatus*  *Sibbaldia cuneata*  *Sibbaldia procumbens*  *Sorbus alnifolia*  *Sorbus coronata*  *Sorbus corymbifera*  *Sorbus epidendron*  *Sorbus folgneri*  *Sorbus hemsleyi*  *Sorbus hupehensis*  *Sorbus insignis*  *Sorbus koehneana*  *Sorbus oligodonta*  *Sorbus pallescens*  *Sorbus pohuashanensis*  *Sorbus prattii*  *Sorbus reducta*  *Sorbus rufopilosa*  *Sorbus setschwanensis*  *Sorbus vilmorinii*  *Sorbus wallichii*  *Sorbus wilsoniana*  *Spenceria ramalana*  *Spiraea alpina*  *Spiraea bella*  *Spiraea calcicola*  *Spiraea canescens*  *Spiraea chinensis*  *Spiraea japonica*  *Spiraea mollifolia*  *Spiraea mongolica*  *Diplospora fruticosa*  *Galium bungei*  *Galium uliginosum*  *Hedyotis uncinella*  *Leptodermis pilosa*  *Rubia alata*  *Rubia cordifolia*  *Rubia podantha*  *Boenninghausenia albiflora*  *Skimmia arborescens*  *Zanthoxylum acanthopodium*  *Zanthoxylum myriacanthum*  *Meliosma cuneifolia*  *Salix myrtillacea*  *Koelreuteria paniculata*  *Sapindus delavayi*  *Bergenia purpurascens*  *Saxifraga litangensis*  *Tiarella polyphylla*  *Hemiphragma heterophyllum*  *Lagotis clarkei*  *Pedicularis superba*  *Ailanthus altissima*  *Datura stramonium*  *Nicandra physalodes*  *Solanum pseudocapsicum*  *Euscaphis japonica*  *Symplocos paniculata*  *Cryptomeria japonica*  *Tetracentron sinense*  *Anneslea fragrans*  *Eurya brevistyla*  *Eurya nitida*  *Schima argentea*  *Schima wallichii*  *Ternstroemia gymnanthera*  *Stellera chamaejasme*  *Trema angustifolia*  *Laportea bulbifera*  *Oreocnide integrifolia*  *Urtica hyperborea*  *Urtica laetevirens*  *Patrinia monandra*  *Callicarpa formosana*  *Callicarpa pseudorubella*  *Callicarpa rubella*  *Caryopteris forrestii*  *Caryopteris tangutica*  *Caryopteris trichosphaera*  *Clerodendrum bungei*  *Verbena officinalis*  *Vitex negundo*  *Ampelopsis acutidentata*  *Cayratia japonica*  *Cayratia pedata*  *Parthenocissus semicordata*  *Tetrastigma obtectum*  *Tetrastigma rumicispermum*  *Tetrastigma serrulatum*  *Tetrastigma yunnanense*  *Vitis betulifolia*  *Hedychium yunnanense*  *Zingiber officinale*  *Zingiber striolatum* | 2  2  2  2  2  2  2  2  2  2  2  5  3  1  1  1  1  1  1  1  1  1  1  1  1  1  1  1  1  1  1  1  1  6  5  1  1  1  1  1  1  1  1  1  2  2  2  2  2  2  2  2  2  2  2  2  2  2  2  2  2  2  2  2  2  2  2  2  2  2  2  2  2  2  2  2  2  2  2  2  2  2  2  2  2  2  2  2  2  2  2  2  2  2  2  2  2  2  2  2  2  2  2  2  2  2  2  2  2  2  2  2  2  2  2  2  2  2  7  4  2  3  1  5  5  5  5  5  2  5  5  1  1  1  1  1  1  1  1  1  1  1  1  1  1  1  1  1  1  1  1  1  1  1  1  1  1  1  1  1  1  1  1  1  1  1  1  1  1  1  1  1  1  1  1  1  1  1  1  1  1  1  1  1  1  1  1  1  1  1  1  1  1  1  1  1  1  1  1  1  1  1  1  1  1  1  1  1  1  1  1  1  1  1  1  1  1  1  1  1  1  1  1  1  1  1  1  1  1  1  1  1  1  1  1  1  1  1  1  1  1  1  1  1  1  1  1  1  1  1  1  1  1  1  1  1  1  1  1  1  1  1  1  1  1  1  1  1  1  1  1  1  1  1  1  1  1  1  1  1  1  1  1  1  1  1  1  1  1  1  1  1  1  1  1  1  1  1  1  1  1  1  1  1  1  1  1  1  1  1  7  2  2  6  8  2  2  4  1  2  5  2  4  7  2  1  2  2  4  2  4  2  2  3  3  1  2  5  3  2  3  1  1  1  1  103  148  186  120  4  4  4  4  4  4  4  4  4  4  4  80  40  30  16  40  14  18  2  2  16  2  35  111  701  743  87  109  115  112  27  25  35  32  33  30  31  30  1  8  8  5  6  4  79  6  6  6  6  6  8  1  1  1  1  1  1  1  649  3  4  4  1  6  4  5  5  2  2  2  2  12  1  1  1  1  1  1  1  1  1  1  1  1  1  1  1  1  1  1  6  2  2  2  1  1  1  1  1  1  1  8  1  1  1  1  1  1  2  2  2  732  225  1  3  3  3  3  3  3  3  1  6  6  3  3  12  21  9  4  6  5  8  6  6  4  6  30  15  2  6  6  7  7  10  6  7  2  4  4  18  11  15  5  8  12  1  1  1  25  40  12  1  10  1  8  2  11  7  40  25  5  4  13  20  11  3  8  6  7  18  79  214  1330  4  6  5  5  5  5  5  81  91  75  85  69  85  88  95  92  1  4  9  12  9  4  4  4  4  4  4  4  4  4  4  4  4  4  4  4  4  4  4  4  4  4  4  4  4  4  4  4  4  4  4  4  4  4  4  4  4  4  4  4  4  4  4  4  4  4  4  4  4  4  4  4  4  4  4  4  4  4  4  4  4  4  4  4  4  4  4  4  4  4  4  4  4  4  4  4  4  4  4  4  6  6  6  6  6  6  6  6  6  3  3  6  6  6  6  6  6  6  3  2  3  12  65  9  12  10  3  45  10  9  39  6  15  8  10  6  5  1  1  1  1  1  1  1  1  1  1  1  1  1  1  4  2  2  4  4  4  4  4  4  4  4  4  4  40  1  8  10  560  159  179  202  116  2  1  10  45  8  12  9  9  7  34  1  1  1  1  1  1  1  1  1  1  1  1  1  1  1  1  1  1  1  1  1  1  1  1  1  1  1  1  1  1  1  1  1  1  1  1  1  1  1  1  1  1  1  1  1  1  1  1  1  1  1  1  1  1  1  1  1  1  1  1  1  1  1  1  1  1  1  1  1  1  1  1  1  1  1  1  1  1  1  1  1  1  1  1  1  1  1  1  1  1  1  1  1  1  1  1  1  1  1  1  1  1  1  1  1  1  1  1  1  36  38  28  30  38  77  78  82  65  1  1  1  1  1  1  1  1  5  5  1  1  1  1  1  1  1  1  1  1  1  1  1  1  1  1  65  1  1  1  16  1  1  1  1  1  1  1  1  1  1  1  1  1  1  1  1  1  1  1  1  2  2  3  3  4  3  4  2  2  2  3  2  1  1  1  1  1  1  1  1  1  1  1  2  3  3  2  2  5  5  3  4  5  3  2  3  2  2  5  1  2  3  3  3  2  2  3  5  1  50  1  1  1  1  1  1  10  10  10  10  10  10  5  10  5  1  1  4  10  4  3  1  1  1  1  1  1  1  1  1  1  1  1  1  1  1  1  10  10  10  10  10  10  10  1  1  1  1  1  1  1  1  1  1  1  1  1  1  1  1  1  1  1  1  1  1  1  1  1  1  1  1  1  1  1  1  1  1  1  1  1  1  1  1  1  1  1  1  1  1  1  1  4  6  8  6  6  4  10  6  10  10  6  6  10  8  10  10  10  6  10  1  8  8  8  8  8  8  8  8  12  2  2  8  5  2  2  2  20  3  3  4  4  4  3  3  473  422  12  112  2  10  1  675  630  92  2  2  5  6  9  50  50  30  30  2  1  1  1  1  1  1  1  4  4  4  4  4  4  3  4  8  1  4  3  2  1  3  2  2  4  24  25  29 | FOS-a  FOS-a  FOS-a  FOS-a  FOS-a  FOS-a  FOS-a  FOS-a  FOS-a  FOS-a  FOS-a  FOS-a  FOS-a  FOS-a  FOS-a  FOS-a  FOS-a  FOS-a  FOS-a  FOS-a  FOS-a  FOS-a  FOS-a  FOS-a  FOS-a  FOS-a  FOS-b  FOS-b  FOS-b  FOS-b  FOS-b  FOS-b  FOS-b  FOS-a  FOS-b  FOS-b  FOS-b  FOS-b  FOS-b  FOS-b  FOS-b  FOS-b  FOS-b  FOS-b  FOS-a  FOS-b  FOS-b  FOS-b  FOS-b  FOS-b  FOS-b  FOS-b  FOS-b  FOS-b  FOS-b  FOS-b  FOS-b  FOS-b  FOS-b  FOS-b  FOS-b  FOS-b  FOS-b  FOS-b  FOS-b  FOS-b  FOS-b  FOS-b  FOS-b  FOS-b  FOS-b  FOS-b  FOS-b  FOS-b  FOS-b  FOS-b  FOS-b  FOS-b  FOS-b  FOS-b  FOS-b  FOS-b  FOS-b  FOS-b  FOS-b  FOS-b  FOS-b  FOS-b  FOS-b  FOS-b  FOS-b  FOS-b  FOS-b  FOS-b  FOS-b  FOS-b  FOS-b  FOS-b  FOS-b  FOS-b  FOS-b  FOS-b  FOS-b  FOS-b  FOS-b  FOS-b  FOS-b  FOS-b  FOS-b  FOS-b  FOS-b  FOS-b  FOS-b  FOS-b  FOS-b  FOS-b  FOS-b  FOS-b  FOS-a  FOS-a  FOS-a  FOS-a  FOS-a  FOS-a  FOS-b  FOS-b  FOS-b  FOS-b  FOS-a  FOS-b  FOS-b  FOS-a  FOS-a  FOS-a  FOS-a  FOS-a  FOS-a  FOS-a  FOS-a  FOS-a  FOS-a  FOS-a  FOS-a  FOS-a  FOS-a  FOS-a  FOS-a  FOS-a  FOS-a  FOS-a  FOS-a  FOS-a  FOS-a  FOS-a  FOS-a  FOS-a  FOS-a  FOS-a  FOS-a  FOS-a  FOS-a  FOS-a  FOS-a  FOS-a  FOS-a  FOS-a  FOS-a  FOS-a  FOS-a  FOS-a  FOS-a  FOS-a  FOS-a  FOS-a  FOS-a  FOS-a  FOS-a  FOS-a  FOS-a  FOS-a  FOS-a  FOS-a  FOS-a  FOS-a  FOS-a  FOS-a  FOS-a  FOS-a  FOS-a  FOS-a  FOS-a  FOS-a  FOS-a  FOS-a  FOS-a  FOS-a  FOS-a  FOS-a  FOS-a  FOS-a  FOS-a  FOS-a  FOS-a  FOS-a  FOS-a  FOS-a  FOS-a  FOS-a  FOS-a  FOS-a  FOS-a  FOS-a  FOS-a  FOS-a  FOS-a  FOS-a  FOS-a  FOS-a  FOS-a  FOS-a  FOS-a  FOS-a  FOS-a  FOS-a  FOS-a  FOS-a  FOS-a  FOS-a  FOS-a  FOS-a  FOS-a  FOS-a  FOS-a  FOS-a  FOS-a  FOS-a  FOS-a  FOS-a  FOS-a  FOS-a  FOS-a  FOS-a  FOS-a  FOS-a  FOS-a  FOS-a  FOS-a  FOS-a  FOS-a  FOS-a  FOS-a  FOS-a  FOS-a  FOS-a  FOS-a  FOS-a  FOS-a  FOS-a  FOS-a  FOS-a  FOS-a  FOS-a  FOS-a  FOS-a  FOS-a  FOS-a  FOS-a  FOS-a  FOS-a  FOS-a  FOS-a  FOS-a  FOS-a  FOS-a  FOS-a  FOS-a  FOS-a  FOS-a  FOS-a  FOS-a  FOS-a  FOS-a  FOS-a  FOS-a  FOS-a  FOS-a  FOS-a  FOS-a  FOS-a  FOS-a  FOS-a  FOS-a  FOS-a  FOS-a  FOS-a  FOS-a  FOS-a  FOS-a  FOS-a  FOS-a  FOS-a  FOS-a  FOS-a  FOS-a  FOS-a  FOS-a  FOS-a  FOS-a  FOS-a  FOS-a  FOS-a  FOS-b  FOS-b  FOS-b  FOS-b  FOS-b  FOS-b  FOS-b  FOS-b  FOS-b  FOS-b  FOS-b  FOS-b  FOS-b  FOS-b  FOS-b  FOS-b  FOS-b  FOS-b  FOS-b  FOS-b  FOS-b  FOS-b  FOS-b  FOS-b  FOS-b  FOS-a  FOS-b  FOS-b  FOS-b  FOS-b  FOS-b  FOS-b  FOS-a  FOS-a  GBOWS  GBOWS  GBOWS  GBOWS  FOS-a  FOS-a  FOS-a  FOS-a  FOS-a  FOS-a  FOS-a  FOS-a  FOS-a  FOS-a  FOS-a  FOS-b  FOS-b  FOS-b  FOS-b  FOS-b  FOS-b  FOS-b  FOS-a  FOS-a  FOS-a  FOS-a  FOS-a  GBOWS  GBOWS  GBOWS  GBOWS  GBOWS  GBOWS  GBOWS  GBOWS  GBOWS  GBOWS  GBOWS  GBOWS  GBOWS  GBOWS  GBOWS  FOS-a  GBOWS  GBOWS  GBOWS  GBOWS  FOS-a  GBOWS  FOS-a  FOS-a  FOS-a  FOS-a  FOS-a  FOS-a  FOS-b  FOS-b  FOS-b  FOS-b  FOS-b  FOS-b  FOS-a  GBOWS  FOS-a  FOS-a  FOS-a  FOS-a  FOS-a  FOS-a  FOS-b  FOS-b  FOS-a  FOS-a  FOS-a  FOS-a  FOS-a  FOS-a  FOS-a  FOS-a  FOS-a  FOS-a  FOS-a  FOS-a  FOS-a  FOS-a  FOS-a  FOS-a  FOS-a  FOS-a  FOS-a  FOS-a  FOS-a  FOS-a  FOS-a  FOS-a  FOS-a  FOS-a  FOS-a  FOS-a  FOS-a  FOS-a  FOS-a  FOS-a  FOS-a  FOS-a  FOS-a  FOS-a  FOS-a  FOS-a  FOS-a  FOS-a  FOS-a  FOS-a  FOS-a  FOS-a  GBOWS  GBOWS  FOS-a  FOS-b  FOS-b  FOS-b  FOS-b  FOS-b  FOS-b  FOS-b  FOS-b  FOS-b  FOS-b  FOS-a  FOS-b  FOS-a  FOS-a  GBOWS  FOS-a  FOS-a  FOS-a  FOS-a  FOS-a  GBOWS  FOS-a  FOS-a  FOS-a  FOS-a  FOS-a  FOS-a  FOS-a  FOS-a  FOS-a  FOS-a  FOS-a  FOS-a  FOS-a  FOS-a  FOS-a  FOS-b  FOS-b  FOS-b  GBOWS  FOS-a  FOS-b  FOS-a  FOS-b  FOS-a  FOS-a  FOS-b  FOS-a  FOS-a  FOS-a  FOS-a  FOS-a  FOS-a  FOS-a  FOS-a  FOS-a  FOS-a  FOS-a  FOS-a  FOS-b  FOS-b  GBOWS  FOS-b  FOS-a  FOS-a  FOS-a  FOS-a  GBOWS  GBOWS  GBOWS  FOS-a  GBOWS  FOS-a  FOS-a  FOS-a  FOS-a  FOS-a  GBOWS  GBOWS  GBOWS  GBOWS  GBOWS  GBOWS  GBOWS  GBOWS  GBOWS  FOS-a  FOS-a  FOS-a  FOS-a  FOS-a  FOS-a  FOS-a  FOS-a  FOS-a  FOS-a  FOS-a  FOS-a  FOS-a  FOS-a  FOS-a  FOS-a  FOS-a  FOS-a  FOS-a  FOS-a  FOS-a  FOS-a  FOS-a  FOS-a  FOS-a  FOS-a  FOS-a  FOS-a  FOS-a  FOS-a  FOS-a  FOS-a  FOS-a  FOS-a  FOS-a  FOS-a  FOS-a  FOS-a  FOS-a  FOS-a  FOS-a  FOS-a  FOS-a  FOS-a  FOS-a  FOS-a  FOS-a  FOS-a  FOS-a  FOS-a  FOS-a  FOS-a  FOS-a  FOS-a  FOS-a  FOS-a  FOS-a  FOS-a  FOS-a  FOS-a  FOS-a  FOS-a  FOS-a  FOS-a  FOS-a  FOS-a  FOS-a  FOS-a  FOS-a  FOS-a  FOS-a  FOS-a  FOS-a  FOS-a  FOS-a  FOS-a  FOS-a  FOS-a  FOS-a  FOS-a  FOS-a  FOS-a  FOS-a  FOS-a  FOS-b  FOS-b  FOS-b  FOS-b  FOS-b  FOS-b  FOS-b  FOS-b  FOS-b  FOS-b  FOS-b  FOS-b  FOS-b  FOS-b  FOS-b  FOS-b  FOS-b  FOS-b  FOS-a  FOS-a  FOS-b  FOS-a  GBOWS  FOS-a  FOS-a  FOS-a  FOS-b  FOS-b  FOS-a  FOS-b  GBOWS  FOS-a  FOS-b  FOS-a  FOS-b  FOS-a  FOS-b  FOS-a  FOS-a  FOS-a  FOS-a  FOS-a  FOS-a  FOS-a  FOS-a  FOS-a  FOS-a  FOS-a  FOS-a  FOS-b  FOS-a  FOS-a  FOS-a  FOS-a  FOS-b  FOS-b  FOS-b  FOS-a  FOS-a  FOS-a  FOS-a  FOS-a  FOS-a  FOS-b  GBOWS  FOS-a  FOS-b  FOS-b  GBOWS  GBOWS  GBOWS  GBOWS  GBOWS  FOS-b  FOS-b  FOS-a  FOS-a  FOS-a  FOS-a  FOS-a  FOS-a  FOS-a  FOS-a  FOS-b  FOS-a  FOS-a  FOS-a  FOS-a  FOS-a  FOS-a  FOS-a  FOS-a  FOS-a  FOS-a  FOS-a  FOS-a  FOS-a  FOS-a  FOS-a  FOS-a  FOS-a  FOS-a  FOS-a  FOS-a  FOS-a  FOS-a  FOS-a  FOS-a  FOS-a  FOS-a  FOS-a  FOS-a  FOS-a  FOS-a  FOS-a  FOS-a  FOS-a  FOS-a  FOS-a  FOS-a  FOS-a  FOS-a  FOS-a  FOS-a  FOS-a  FOS-a  FOS-a  FOS-a  FOS-a  FOS-a  FOS-a  FOS-a  FOS-a  FOS-a  FOS-a  FOS-a  FOS-a  FOS-a  FOS-a  FOS-a  FOS-a  FOS-a  FOS-a  FOS-a  FOS-a  FOS-a  FOS-a  FOS-a  FOS-a  FOS-a  FOS-a  FOS-a  FOS-a  FOS-a  FOS-a  FOS-a  FOS-a  FOS-a  FOS-a  FOS-a  FOS-a  FOS-a  FOS-a  FOS-a  FOS-a  FOS-a  FOS-a  FOS-a  FOS-a  FOS-a  FOS-a  FOS-a  FOS-a  FOS-a  FOS-a  FOS-a  FOS-a  FOS-a  FOS-a  FOS-a  FOS-a  FOS-a  FOS-a  FOS-a  FOS-a  FOS-a  FOS-a  FOS-a  FOS-a  FOS-a  FOS-a  FOS-a  GBOWS  GBOWS  GBOWS  GBOWS  GBOWS  GBOWS  GBOWS  FOS-a  FOS-a  FOS-a  FOS-a  FOS-a  FOS-a  FOS-a  FOS-a  FOS-a  FOS-a  FOS-b  FOS-a  FOS-b  FOS-b  FOS-b  FOS-b  FOS-b  FOS-b  FOS-a  FOS-b  FOS-b  FOS-b  FOS-a  FOS-b  FOS-b  FOS-a  FOS-b  FOS-b  GBOWS  FOS-a  FOS-a  FOS-a  FOS-a  FOS-a  FOS-a  FOS-a  FOS-a  FOS-a  FOS-a  FOS-a  FOS-a  FOS-a  FOS-a  FOS-a  FOS-a  FOS-a  FOS-a  FOS-a  FOS-a  FOS-a  FOS-a  FOS-a  FOS-a  FOS-a  FOS-a  FOS-a  FOS-a  FOS-a  FOS-a  FOS-a  FOS-a  FOS-a  FOS-a  FOS-a  FOS-a  FOS-a  FOS-a  FOS-a  FOS-a  FOS-a  FOS-a  FOS-a  FOS-a  FOS-a  FOS-a  FOS-a  FOS-a  FOS-a  FOS-a  FOS-a  FOS-a  FOS-a  FOS-a  FOS-a  FOS-a  FOS-a  FOS-a  FOS-a  FOS-a  FOS-a  FOS-a  FOS-a  FOS-a  FOS-a  FOS-a  FOS-a  FOS-a  FOS-a  FOS-a  FOS-a  FOS-a  FOS-a  FOS-a  FOS-a  FOS-a  FOS-a  FOS-a  FOS-a  FOS-a  FOS-a  FOS-a  FOS-a  FOS-a  FOS-a  FOS-a  FOS-a  FOS-a  FOS-a  FOS-a  FOS-a  FOS-a  FOS-a  GBOWS  FOS-a  FOS-a  FOS-a  FOS-a  FOS-a  FOS-a  FOS-a  FOS-a  FOS-a  FOS-a  FOS-a  FOS-a  FOS-a  FOS-a  FOS-a  FOS-a  FOS-a  FOS-a  FOS-a  FOS-a  FOS-a  FOS-b  FOS-b  FOS-b  FOS-b  FOS-b  FOS-b  FOS-b  FOS-b  FOS-b  FOS-b  FOS-b  FOS-b  FOS-b  FOS-b  FOS-b  FOS-b  FOS-b  FOS-b  FOS-b  FOS-b  FOS-b  FOS-b  FOS-b  FOS-b  FOS-b  FOS-b  FOS-b  FOS-b  FOS-b  FOS-b  FOS-a  FOS-a  FOS-a  FOS-a  FOS-a  FOS-a  FOS-a  FOS-a  FOS-a  FOS-a  FOS-a  FOS-a  FOS-a  FOS-a  FOS-a  FOS-a  FOS-a  FOS-a  FOS-a  FOS-a  FOS-a  FOS-a  FOS-a  FOS-a  FOS-a  FOS-a  FOS-a  FOS-a  FOS-a  FOS-a  FOS-a  FOS-a  FOS-a  FOS-a  FOS-a  FOS-a  FOS-a  FOS-a  FOS-a  FOS-a  FOS-a  FOS-b  FOS-b  FOS-b  FOS-b  FOS-b  FOS-b  FOS-b  FOS-b  FOS-a  FOS-b  FOS-b  FOS-a  FOS-a  FOS-a  FOS-a  FOS-a  FOS-a  FOS-a  FOS-b  FOS-b  FOS-b  FOS-a  FOS-a  FOS-b  GBOWS  GBOWS  FOS-a  GBOWS  FOS-a  GBOWS  FOS-a  GBOWS  GBOWS  GBOWS  FOS-a  FOS-a  FOS-a  FOS-a  FOS-a  FOS-b  FOS-b  FOS-b  FOS-b  FOS-a  FOS-a  FOS-a  FOS-a  FOS-b  FOS-a  FOS-a  FOS-a  FOS-a  FOS-a  FOS-a  FOS-b  FOS-b  FOS-b  FOS-a  FOS-b  FOS-b  FOS-a  FOS-a  FOS-a  FOS-a  FOS-a  FOS-a  FOS-a  FOS-a  FOS-a  GBOWS  GBOWS  GBOWS | <http://frps.eflora.cn/frps/Acer>  <http://frps.eflora.cn/frps/Acer>  <http://frps.eflora.cn/frps/Acer>  <http://frps.eflora.cn/frps/Acer>  <http://frps.eflora.cn/frps/Acer>  <http://frps.eflora.cn/frps/Acer>  <http://frps.eflora.cn/frps/Acer>  <http://frps.eflora.cn/frps/Acer>  <http://frps.eflora.cn/frps/Acer>  <http://frps.eflora.cn/frps/Acer>  <http://frps.eflora.cn/frps/Acer>  <http://frps.eflora.cn/frps/Sambucus>  <http://frps.eflora.cn/frps/Sambucus%20williamsii>  <http://frps.eflora.cn/frps/Viburnum>  <http://frps.eflora.cn/frps/Viburnum>  <http://frps.eflora.cn/frps/Viburnum>  <http://frps.eflora.cn/frps/Viburnum>  <http://frps.eflora.cn/frps/Viburnum>  <http://frps.eflora.cn/frps/Viburnum>  <http://frps.eflora.cn/frps/Viburnum>  <http://frps.eflora.cn/frps/Viburnum>  <http://frps.eflora.cn/frps/Viburnum>  <http://frps.eflora.cn/frps/Viburnum>  <http://frps.eflora.cn/frps/Viburnum>  <http://frps.eflora.cn/frps/Achyranthes>  <http://frps.eflora.cn/frps/Achyranthes>  <http://frps.eflora.cn/frps/Amaranthus>  <http://frps.eflora.cn/frps/Amaranthus>  <http://frps.eflora.cn/frps/Amaranthus>  <http://frps.eflora.cn/frps/Amaranthus>  <http://frps.eflora.cn/frps/Celosia%20argentea>  <http://frps.eflora.cn/frps/Cyathula>  <http://frps.eflora.cn/frps/Cyathula>  <http://frps.eflora.cn/frps?id=%E6%B5%86%E6%9E%9C%E8%8B%8B>  <http://frps.eflora.cn/frps/Choerospondias>  <http://frps.eflora.cn/frps/Pistacia>  <http://frps.eflora.cn/frps/Pistacia>  <http://frps.eflora.cn/frps/Rhus>  <http://frps.eflora.cn/frps/Rhus>  <http://frps.eflora.cn/frps/Rhus>  <http://frps.eflora.cn/frps/Toxicodendron>  <http://frps.eflora.cn/frps/Toxicodendron>  <http://frps.eflora.cn/frps/Toxicodendron>  <http://frps.eflora.cn/frps/Toxicodendron>  [http://frps.eflora.cn/frps/Polyalthia](http://frps.eflora.cn/frps/Polyalthia%20cerasoides)  <http://frps.eflora.cn/frps/Umbelliferae>  <http://frps.eflora.cn/frps/Umbelliferae>  <http://frps.eflora.cn/frps/Umbelliferae>  <http://frps.eflora.cn/frps/Umbelliferae>  <http://frps.eflora.cn/frps/Umbelliferae>  <http://frps.eflora.cn/frps/Umbelliferae>  <http://frps.eflora.cn/frps/Umbelliferae>  <http://frps.eflora.cn/frps/Umbelliferae>  <http://frps.eflora.cn/frps/Umbelliferae>  <http://frps.eflora.cn/frps/Umbelliferae>  <http://frps.eflora.cn/frps/Umbelliferae>  <http://frps.eflora.cn/frps/Umbelliferae>  <http://frps.eflora.cn/frps/Umbelliferae>  <http://frps.eflora.cn/frps/Umbelliferae>  <http://frps.eflora.cn/frps/Umbelliferae>  <http://frps.eflora.cn/frps/Umbelliferae>  <http://frps.eflora.cn/frps/Umbelliferae>  <http://frps.eflora.cn/frps/Umbelliferae>  <http://frps.eflora.cn/frps/Umbelliferae>  <http://frps.eflora.cn/frps/Umbelliferae>  <http://frps.eflora.cn/frps/Umbelliferae>  <http://frps.eflora.cn/frps/Umbelliferae>  <http://frps.eflora.cn/frps/Umbelliferae>  <http://frps.eflora.cn/frps/Umbelliferae>  <http://frps.eflora.cn/frps/Umbelliferae>  <http://frps.eflora.cn/frps/Umbelliferae>  <http://frps.eflora.cn/frps/Umbelliferae>  <http://frps.eflora.cn/frps/Umbelliferae>  <http://frps.eflora.cn/frps/Umbelliferae>  <http://frps.eflora.cn/frps/Umbelliferae>  <http://frps.eflora.cn/frps/Umbelliferae>  <http://frps.eflora.cn/frps/Umbelliferae>  <http://frps.eflora.cn/frps/Umbelliferae>  <http://frps.eflora.cn/frps/Umbelliferae>  <http://frps.eflora.cn/frps/Umbelliferae>  <http://frps.eflora.cn/frps/Umbelliferae>  <http://frps.eflora.cn/frps/Umbelliferae>  <http://frps.eflora.cn/frps/Umbelliferae>  <http://frps.eflora.cn/frps/Umbelliferae>  <http://frps.eflora.cn/frps/Umbelliferae>  <http://frps.eflora.cn/frps/Umbelliferae>  <http://frps.eflora.cn/frps/Umbelliferae>  <http://frps.eflora.cn/frps/Umbelliferae>  <http://frps.eflora.cn/frps/Umbelliferae>  <http://frps.eflora.cn/frps/Umbelliferae>  <http://frps.eflora.cn/frps/Umbelliferae>  <http://frps.eflora.cn/frps/Umbelliferae>  <http://frps.eflora.cn/frps/Umbelliferae>  <http://frps.eflora.cn/frps/Umbelliferae>  <http://frps.eflora.cn/frps/Umbelliferae>  <http://frps.eflora.cn/frps/Umbelliferae>  <http://frps.eflora.cn/frps/Umbelliferae>  <http://frps.eflora.cn/frps/Umbelliferae>  <http://frps.eflora.cn/frps/Umbelliferae>  <http://frps.eflora.cn/frps/Umbelliferae>  <http://frps.eflora.cn/frps/Umbelliferae>  <http://frps.eflora.cn/frps/Umbelliferae>  <http://frps.eflora.cn/frps/Umbelliferae>  <http://frps.eflora.cn/frps/Umbelliferae>  <http://frps.eflora.cn/frps/Umbelliferae>  <http://frps.eflora.cn/frps/Umbelliferae>  <http://frps.eflora.cn/frps/Umbelliferae>  <http://frps.eflora.cn/frps/Umbelliferae>  <http://frps.eflora.cn/frps/Umbelliferae>  <http://frps.eflora.cn/frps/Umbelliferae>  <http://frps.eflora.cn/frps/Umbelliferae>  <http://frps.eflora.cn/frps/Umbelliferae>  <http://frps.eflora.cn/frps/Umbelliferae>  <http://frps.eflora.cn/frps/Umbelliferae>  <http://frps.eflora.cn/frps/Umbelliferae>  <http://frps.eflora.cn/frps/Umbelliferae>  <http://frps.eflora.cn/frps/Umbelliferae>  <http://frps.eflora.cn/frps/Umbelliferae>  <http://frps.eflora.cn/frps/Ilex%20polyneura>  <http://frps.eflora.cn/frps/Ser.%20Repandae>  <http://frps.eflora.cn/frps/Arisaema%20heterophyllum>  <http://frps.eflora.cn/frps/Arisaema%20flavum>  <http://frps.eflora.cn/frps/Arisaema%20heterophyllum>  <http://frps.eflora.cn/frps/Arisaema%20tortuosum>  <http://frps.eflora.cn/frps/Aralia>  <http://frps.eflora.cn/frps/Aralia>  <http://frps.eflora.cn/frps/Aralia>  <http://frps.eflora.cn/frps/Aralia>  http://frps.eflora.cn/frps/Brassaiopsis  http://frps.eflora.cn/frps?id=%E7%BA%A2%E6%AF%9B%E4%BA%94%E5%8A%A0  <http://frps.eflora.cn/frps/Schefflera%20minutistellata>  <http://frps.eflora.cn/frps/Compositae>  <http://frps.eflora.cn/frps/Compositae>  <http://frps.eflora.cn/frps/Compositae>  <http://frps.eflora.cn/frps/Compositae>  <http://frps.eflora.cn/frps/Compositae>  <http://frps.eflora.cn/frps/Compositae>  <http://frps.eflora.cn/frps/Compositae>  <http://frps.eflora.cn/frps/Compositae>  <http://frps.eflora.cn/frps/Compositae>  <http://frps.eflora.cn/frps/Compositae>  <http://frps.eflora.cn/frps/Compositae>  <http://frps.eflora.cn/frps/Compositae>  <http://frps.eflora.cn/frps/Compositae>  <http://frps.eflora.cn/frps/Compositae>  <http://frps.eflora.cn/frps/Compositae>  <http://frps.eflora.cn/frps/Compositae>  <http://frps.eflora.cn/frps/Compositae>  <http://frps.eflora.cn/frps/Compositae>  <http://frps.eflora.cn/frps/Compositae>  <http://frps.eflora.cn/frps/Compositae>  <http://frps.eflora.cn/frps/Compositae>  <http://frps.eflora.cn/frps/Compositae>  <http://frps.eflora.cn/frps/Compositae>  <http://frps.eflora.cn/frps/Compositae>  <http://frps.eflora.cn/frps/Compositae>  <http://frps.eflora.cn/frps/Compositae>  <http://frps.eflora.cn/frps/Compositae>  <http://frps.eflora.cn/frps/Compositae>  <http://frps.eflora.cn/frps/Compositae>  <http://frps.eflora.cn/frps/Compositae>  <http://frps.eflora.cn/frps/Compositae>  <http://frps.eflora.cn/frps/Compositae>  <http://frps.eflora.cn/frps/Compositae>  <http://frps.eflora.cn/frps/Compositae>  <http://frps.eflora.cn/frps/Compositae>  <http://frps.eflora.cn/frps/Compositae>  <http://frps.eflora.cn/frps/Compositae>  <http://frps.eflora.cn/frps/Compositae>  <http://frps.eflora.cn/frps/Compositae>  <http://frps.eflora.cn/frps/Compositae>  <http://frps.eflora.cn/frps/Compositae>  <http://frps.eflora.cn/frps/Compositae>  <http://frps.eflora.cn/frps/Compositae>  <http://frps.eflora.cn/frps/Compositae>  <http://frps.eflora.cn/frps/Compositae>  <http://frps.eflora.cn/frps/Compositae>  <http://frps.eflora.cn/frps/Compositae>  <http://frps.eflora.cn/frps/Compositae>  <http://frps.eflora.cn/frps/Compositae>  <http://frps.eflora.cn/frps/Compositae>  <http://frps.eflora.cn/frps/Compositae>  <http://frps.eflora.cn/frps/Compositae>  <http://frps.eflora.cn/frps/Compositae>  <http://frps.eflora.cn/frps/Compositae>  <http://frps.eflora.cn/frps/Compositae>  <http://frps.eflora.cn/frps/Compositae>  <http://frps.eflora.cn/frps/Compositae>  <http://frps.eflora.cn/frps/Compositae>  <http://frps.eflora.cn/frps/Compositae>  http://frps.eflora.cn/frps/Compositae  <http://frps.eflora.cn/frps/Compositae>  <http://frps.eflora.cn/frps/Compositae>  <http://frps.eflora.cn/frps/Compositae>  <http://frps.eflora.cn/frps/Compositae>  http://frps.eflora.cn/frps/Compositae  <http://frps.eflora.cn/frps/Compositae>  <http://frps.eflora.cn/frps/Compositae>  <http://frps.eflora.cn/frps/Compositae>  <http://frps.eflora.cn/frps/Compositae>  <http://frps.eflora.cn/frps/Compositae>  <http://frps.eflora.cn/frps/Compositae>  <http://frps.eflora.cn/frps/Compositae>  <http://frps.eflora.cn/frps/Compositae>  <http://frps.eflora.cn/frps/Compositae>  <http://frps.eflora.cn/frps/Compositae>  <http://frps.eflora.cn/frps/Compositae>  <http://frps.eflora.cn/frps/Compositae>  <http://frps.eflora.cn/frps/Compositae>  <http://frps.eflora.cn/frps/Compositae>  <http://frps.eflora.cn/frps/Compositae>  <http://frps.eflora.cn/frps/Compositae>  <http://frps.eflora.cn/frps/Compositae>  <http://frps.eflora.cn/frps/Compositae>  <http://frps.eflora.cn/frps/Compositae>  <http://frps.eflora.cn/frps/Compositae>  <http://frps.eflora.cn/frps/Compositae>  <http://frps.eflora.cn/frps/Compositae>  <http://frps.eflora.cn/frps/Compositae>  <http://frps.eflora.cn/frps/Compositae>  <http://frps.eflora.cn/frps/Compositae>  <http://frps.eflora.cn/frps/Compositae>  <http://frps.eflora.cn/frps/Compositae>  <http://frps.eflora.cn/frps/Compositae>  <http://frps.eflora.cn/frps/Compositae>  <http://frps.eflora.cn/frps/Compositae>  <http://frps.eflora.cn/frps/Compositae>  <http://frps.eflora.cn/frps/Compositae>  <http://frps.eflora.cn/frps/Compositae>  <http://frps.eflora.cn/frps/Compositae>  <http://frps.eflora.cn/frps/Compositae>  <http://frps.eflora.cn/frps/Compositae>  <http://frps.eflora.cn/frps/Compositae>  <http://frps.eflora.cn/frps/Compositae>  <http://frps.eflora.cn/frps/Compositae>  <http://frps.eflora.cn/frps/Compositae>  <http://frps.eflora.cn/frps/Compositae>  <http://frps.eflora.cn/frps/Compositae>  <http://frps.eflora.cn/frps/Compositae>  <http://frps.eflora.cn/frps/Compositae>  <http://frps.eflora.cn/frps/Compositae>  <http://frps.eflora.cn/frps/Compositae>  <http://frps.eflora.cn/frps/Compositae>  <http://frps.eflora.cn/frps/Compositae>  <http://frps.eflora.cn/frps/Compositae>  <http://frps.eflora.cn/frps/Compositae>  <http://frps.eflora.cn/frps/Compositae>  <http://frps.eflora.cn/frps/Compositae>  <http://frps.eflora.cn/frps/Compositae>  <http://frps.eflora.cn/frps/Compositae>  <http://frps.eflora.cn/frps/Compositae>  <http://frps.eflora.cn/frps/Compositae>  <http://frps.eflora.cn/frps/Compositae>  <http://frps.eflora.cn/frps/Compositae>  <http://frps.eflora.cn/frps/Compositae>  <http://frps.eflora.cn/frps/Compositae>  <http://frps.eflora.cn/frps/Compositae>  <http://frps.eflora.cn/frps/Compositae>  <http://frps.eflora.cn/frps/Compositae>  <http://frps.eflora.cn/frps/Compositae>  <http://frps.eflora.cn/frps/Compositae>  <http://frps.eflora.cn/frps/Compositae>  <http://frps.eflora.cn/frps/Compositae>  <http://frps.eflora.cn/frps/Compositae>  <http://frps.eflora.cn/frps/Compositae>  <http://frps.eflora.cn/frps/Compositae>  <http://frps.eflora.cn/frps/Compositae>  <http://frps.eflora.cn/frps/Compositae>  <http://frps.eflora.cn/frps/Compositae>  <http://frps.eflora.cn/frps/Compositae>  <http://frps.eflora.cn/frps/Compositae>  <http://frps.eflora.cn/frps/Compositae>  <http://frps.eflora.cn/frps/Compositae>  <http://frps.eflora.cn/frps/Compositae>  <http://frps.eflora.cn/frps/Compositae>  <http://frps.eflora.cn/frps/Compositae>  <http://frps.eflora.cn/frps/Compositae>  <http://frps.eflora.cn/frps/Compositae>  <http://frps.eflora.cn/frps/Compositae>  <http://frps.eflora.cn/frps/Compositae>  <http://frps.eflora.cn/frps/Compositae>  <http://frps.eflora.cn/frps/Compositae>  <http://frps.eflora.cn/frps/Compositae>  <http://frps.eflora.cn/frps/Compositae>  <http://frps.eflora.cn/frps/Compositae>  <http://frps.eflora.cn/frps/Compositae>  http://frps.eflora.cn/frps/Compositae  <http://frps.eflora.cn/frps/Compositae>  <http://frps.eflora.cn/frps/Compositae>  <http://frps.eflora.cn/frps/Compositae>  <http://frps.eflora.cn/frps/Compositae>  <http://frps.eflora.cn/frps/Compositae>  <http://frps.eflora.cn/frps/Compositae>  <http://frps.eflora.cn/frps/Compositae>  <http://frps.eflora.cn/frps/Compositae>  <http://frps.eflora.cn/frps/Compositae>  <http://frps.eflora.cn/frps/Compositae>  <http://frps.eflora.cn/frps/Compositae>  <http://frps.eflora.cn/frps/Compositae>  http://frps.eflora.cn/frps/Compositae  <http://frps.eflora.cn/frps/Compositae>  <http://frps.eflora.cn/frps/Compositae>  <http://frps.eflora.cn/frps/Compositae>  <http://frps.eflora.cn/frps/Compositae>  <http://frps.eflora.cn/frps/Compositae>  <http://frps.eflora.cn/frps/Compositae>  <http://frps.eflora.cn/frps/Compositae>  <http://frps.eflora.cn/frps/Compositae>  <http://frps.eflora.cn/frps/Compositae>  <http://frps.eflora.cn/frps/Compositae>  <http://frps.eflora.cn/frps/Impatiens%20lateristachys>  <http://frps.eflora.cn/frps/Berberis%20agricola>  <http://frps.eflora.cn/frps/Berberis%20amoena>  http://frps.eflora.cn/frps/Berberis%20approximata  http://frps.eflora.cn/frps/Berberis%20concinna  http://frps.eflora.cn/frps/Berberis%20davidii  http://frps.eflora.cn/frps/Berberis%20dictyoneura  http://frps.eflora.cn/frps/Berberis%20dictyophylla  http://frps.eflora.cn/frps/Berberis%20ferdinandi-coburgii  http://frps.eflora.cn/frps/Berberis%20franchetiana  http://frps.eflora.cn/frps/Berberis%20gyalaica  http://frps.eflora.cn/frps/Berberis%20henryana  http://frps.eflora.cn/frps/Berberis%20ignorata  http://frps.eflora.cn/frps/Berberis%20insignis  http://frps.eflora.cn/frps/Berberis%20jamesiana  http://frps.eflora.cn/frps/Berberis%20julianae  http://frps.eflora.cn/frps/Berberis%20kansuensis  http://frps.eflora.cn/frps/Berberis%20lecomtei  http://frps.eflora.cn/frps/Berberis%20metapolyantha  http://frps.eflora.cn/frps/Berberis%20minutiflora  http://frps.eflora.cn/frps/Berberis%20muliensis  http://frps.eflora.cn/frps/Berberis%20obovatifolia  http://frps.eflora.cn/frps/Berberis%20papillifera  http://frps.eflora.cn/frps/Berberis%20pruinosa  http://frps.eflora.cn/frps/Berberis%20reticulinervis  http://frps.eflora.cn/frps/Berberis%20sublevis  http://frps.eflora.cn/frps/Berberis%20tsarongensis  http://frps.eflora.cn/frps/Berberis%20wilsonae  http://frps.eflora.cn/frps/Berberis%20yunnanensis  http://frps.eflora.cn/frps/Mahonia%20fortunei  http://frps.eflora.cn/frps/Nandina%20domestica  http://frps.eflora.cn/frps/Alnus  http://frps.eflora.cn/frps/Alnus  http://frps.eflora.cn/frps/Betula  http://frps.eflora.cn/frps/Betula  http://frps.eflora.cn/frps/Boraginaceae  http://frps.eflora.cn/frps/Boraginaceae  http://frps.eflora.cn/frps/Boraginaceae  http://frps.eflora.cn/frps/Boraginaceae  http://frps.eflora.cn/frps/Boraginaceae  http://frps.eflora.cn/frps/Boraginaceae  http://frps.eflora.cn/frps/Boraginaceae  http://frps.eflora.cn/frps/Boraginaceae  http://frps.eflora.cn/frps/Boraginaceae  http://frps.eflora.cn/frps/Boraginaceae  <http://frps.eflora.cn/frps/Boraginaceae>  http://foc.eflora.cn/content.aspx?TaxonId=200009220  http://foc.eflora.cn/content.aspx?TaxonId=105575  http://foc.eflora.cn/content.aspx?TaxonId=200009318  http://foc.eflora.cn/content.aspx?TaxonId=200009328  http://foc.eflora.cn/content.aspx?TaxonId=200009343  http://foc.eflora.cn/content.aspx?TaxonId=200009347  <http://foc.eflora.cn/content.aspx?TaxonId=200009354>  http://frps.eflora.cn/frps/Megacarpaea  http://frps.eflora.cn/frps/Megacarpaea  http://frps.eflora.cn/frps/Thlaspi%20arvense  http://frps.eflora.cn/frps/Sarcococca  <http://frps.eflora.cn/frps/Calycanthus%20floridus>  http://frps.eflora.cn/frps/Cannabis%20sativa  http://frps.eflora.cn/frps/Arenaria%20yunnanensis  http://frps.eflora.cn/frps/Stellaria%20yunnanensis  http://frps.eflora.cn/frps/Celastrus  http://frps.eflora.cn/frps/Celastrus  http://frps.eflora.cn/frps/Celastrus  http://frps.eflora.cn/frps/Euonymus  http://frps.eflora.cn/frps/Sect.%20Euonymus  http://frps.eflora.cn/frps/Chenopodiaceae  http://frps.eflora.cn/frps/Chenopodiaceae  http://frps.eflora.cn/frps/Chenopodiaceae  http://frps.eflora.cn/frps/Chenopodiaceae  http://frps.eflora.cn/frps/Chenopodiaceae  http://frps.eflora.cn/frps/Chenopodiaceae  http://frps.eflora.cn/frps/Circaeaster  http://frps.eflora.cn/frps/Commelina%20paludosa  http://frps.eflora.cn/frps/Cuscuta  http://frps.eflora.cn/frps/Cuscuta  http://frps.eflora.cn/frps?id=%E9%A3%9E%E8%9B%BE%E8%97%A4  http://frps.eflora.cn/frps/Pharbitis  http://frps.eflora.cn/frps/Merremia%20hederacea  http://frps.eflora.cn/frps/Coriaria%20nepalensis  http://frps.eflora.cn/frps/Coriaria%20terminalis  http://frps.eflora.cn/frps/Swida  http://frps.eflora.cn/frps/Swida  http://frps.eflora.cn/frps/Swida  http://frps.eflora.cn/frps/Swida  http://frps.eflora.cn/frps/Platycladus  http://frps.eflora.cn/frps/Blysmus%20sinocompressus  http://frps.eflora.cn/frps/Cyperaceae  http://frps.eflora.cn/frps/Cyperaceae  http://frps.eflora.cn/frps/Cyperaceae  http://frps.eflora.cn/frps/Cyperaceae  http://frps.eflora.cn/frps/Cyperaceae  http://frps.eflora.cn/frps/Cyperaceae  http://frps.eflora.cn/frps/Cyperaceae  http://frps.eflora.cn/frps/Cyperaceae  http://frps.eflora.cn/frps/Cyperaceae  http://frps.eflora.cn/frps/Cyperaceae  http://frps.eflora.cn/frps/Cyperaceae  http://frps.eflora.cn/frps?id=%E7%89%9B%E6%AF%9B%E6%AF%A1  http://frps.eflora.cn/frps/Cyperaceae  http://frps.eflora.cn/frps/Cyperaceae  http://frps.eflora.cn/frps/Cyperaceae  http://frps.eflora.cn/frps/Cyperaceae  http://frps.eflora.cn/frps/Cyperaceae  http://frps.eflora.cn/frps/Dioscorea%20bulbifera  http://frps.eflora.cn/frps/Dioscorea%20collettii  http://frps.eflora.cn/frps/Dioscorea%20hemsleyi  http://frps.eflora.cn/frps/Dioscorea%20kamoonensis  http://frps.eflora.cn/frps/Dipsacus  http://frps.eflora.cn/frps/Dipsacus  http://frps.eflora.cn/frps/Dipsacus  http://frps.eflora.cn/frps/Dipsacus  http://frps.eflora.cn/frps/Dipsacus  http://frps.eflora.cn/frps/Dipsacus  http://frps.eflora.cn/frps/Dipsacus  http://frps.eflora.cn/frps/Diospyros%20lotus  http://frps.eflora.cn/frps/Elaeagnus  http://frps.eflora.cn/frps/Elaeagnus  http://frps.eflora.cn/frps/Elaeagnus  http://frps.eflora.cn/frps/Hippophae  http://frps.eflora.cn/frps/Hippophae  http://frps.eflora.cn/frps/Hippophae  http://frps.eflora.cn/frps/Ephedra%20gerardiana  http://frps.eflora.cn/frps/Ephedra%20likiangensis  http://frps.eflora.cn/frps/Ephedra%20minuta  http://frps.eflora.cn/frps/Eucommia  http://frps.eflora.cn/frps/Euphorbia  http://frps.eflora.cn/frps/Euphorbia  http://frps.eflora.cn/frps/Euphorbia  http://frps.eflora.cn/frps/Euphorbia  http://frps.eflora.cn/frps/Euphorbia  http://frps.eflora.cn/frps/Euphorbia  http://frps.eflora.cn/frps/Excoecaria  http://frps.eflora.cn/frps/Macaranga%20indica  http://frps.eflora.cn/frps/Sect.%20Phyllanthus  http://frps.eflora.cn/frps/Sect.%21Phyllanthus  http://frps.eflora.cn/frps?id=%E4%B9%8C%E6%A1%95  http://frps.eflora.cn/frps?id=%E9%A2%86%E6%98%A5%E6%9C%A8  http://frps.eflora.cn/frps/Acacia%20pennata  http://frps.eflora.cn/frps/Apios%20carnea  http://frps.eflora.cn/frps/Astragalus%20degensis  http://frps.eflora.cn/frps/Astragalus%20ernestii  http://frps.eflora.cn/frps/Astragalus%20floridus  http://frps.eflora.cn/frps/Astragalus%20monbeigii  http://frps.eflora.cn/frps/Astragalus%20strictus  http://frps.eflora.cn/frps/Bauhinia%20brachycarpa  http://frps.eflora.cn/frps/Cajanus%20cajan  http://frps.eflora.cn/frps/Crotalaria%20pallida  http://frps.eflora.cn/frps/Crotalaria%20sessiliflora  http://frps.eflora.cn/frps/Dalbergia%20mimosoides  http://frps.eflora.cn/frps/Dendrolobium%20triangulare  http://frps.eflora.cn/frps/Desmodium%20elegans  http://frps.eflora.cn/frps/Desmodium%20heterocarpon  http://frps.eflora.cn/frps/Desmodium%20multiflorum  http://frps.eflora.cn/frps/Desmodium%20sequax  http://frps.eflora.cn/frps/Desmodium%20styracifolium  http://frps.eflora.cn/frps/Desmodium%20yunnanense  http://frps.eflora.cn/frps/Flemingia%20latifolia  http://frps.eflora.cn/frps/Hedysarum%20citrinum  http://frps.eflora.cn/frps/Hedysarum%20tanguticum  <http://frps.eflora.cn/frps/Indigofera%20amblyantha>  http://frps.eflora.cn/frps/Indigofera%20balfouriana  http://frps.eflora.cn/frps/Indigofera%20delavayi  http://frps.eflora.cn/frps/Indigofera%20nigrescens  http://frps.eflora.cn/frps/Indigofera%20pendula  http://frps.eflora.cn/frps/Kummerowia  http://frps.eflora.cn/frps/Lespedeza  http://frps.eflora.cn/frps/Lespedeza  http://frps.eflora.cn/frps/Leucaena%20leucocephala  http://frps.eflora.cn/frps/Lotus%20corniculatus  http://frps.eflora.cn/frps/Medicago%20edgeworthii  http://frps.eflora.cn/frps/Medicago%20lupulina  http://frps.eflora.cn/frps/Medicago%20minima  http://frps.eflora.cn/frps/Melilotus%20indicus  http://frps.eflora.cn/frps/Piptanthus%20nepalensis  http://frps.eflora.cn/frps/Rhynchosia%20minima  http://frps.eflora.cn/frps/Robinia%20pseudoacacia  http://frps.eflora.cn/frps/Salweenia%20wardii  http://frps.eflora.cn/frps?id=%E6%9C%9B%E6%B1%9F%E5%8D%97  http://frps.eflora.cn/frps?id=%E5%86%B3%E6%98%8E  http://frps.eflora.cn/frps/Sophora%20davidii  http://frps.eflora.cn/frps/Sophora%20velutina%20var.%20velutina  http://frps.eflora.cn/frps/Thermopsis%20barbata  http://frps.eflora.cn/frps/Thermopsis%20lanceolata  http://frps.eflora.cn/frps/Trifolium%20repens  http://frps.eflora.cn/frps/Vicia%20bungei  http://frps.eflora.cn/frps/Vicia%20cracca  http://frps.eflora.cn/frps/Vicia%20unijuga  http://frps.eflora.cn/frps/Vigna%20vexillata  http://frps.eflora.cn/frps/Swertia%20macrosperma  http://frps.eflora.cn/frps/Geranium  http://frps.eflora.cn/frps/Geranium  http://frps.eflora.cn/frps/Geranium  http://frps.eflora.cn/frps/Geranium  http://frps.eflora.cn/frps/Geranium  http://frps.eflora.cn/frps/Triglochin  http://frps.eflora.cn/frps/Labiatae  http://frps.eflora.cn/frps/Albizia%20bracteata  http://frps.eflora.cn/frps/Albizia%20kalkora  http://frps.eflora.cn/frps/Albizia%20lucidior  http://frps.eflora.cn/frps/Labiatae  http://frps.eflora.cn/frps/Labiatae  http://frps.eflora.cn/frps/Labiatae  http://frps.eflora.cn/frps/Labiatae  http://frps.eflora.cn/frps/Labiatae  http://frps.eflora.cn/frps/Labiatae  http://frps.eflora.cn/frps/Labiatae  http://frps.eflora.cn/frps/Labiatae  http://frps.eflora.cn/frps/Labiatae  http://frps.eflora.cn/frps/Labiatae  http://frps.eflora.cn/frps/Labiatae  http://frps.eflora.cn/frps/Labiatae  http://frps.eflora.cn/frps/Labiatae  http://frps.eflora.cn/frps/Labiatae  http://frps.eflora.cn/frps/Labiatae  http://frps.eflora.cn/frps/Labiatae  http://frps.eflora.cn/frps/Labiatae  http://frps.eflora.cn/frps/Labiatae  http://frps.eflora.cn/frps/Labiatae  http://frps.eflora.cn/frps/Labiatae  http://frps.eflora.cn/frps/Labiatae  http://frps.eflora.cn/frps/Labiatae  http://frps.eflora.cn/frps/Labiatae  http://frps.eflora.cn/frps/Labiatae  http://frps.eflora.cn/frps/Labiatae  http://frps.eflora.cn/frps/Labiatae  http://frps.eflora.cn/frps/Labiatae  http://frps.eflora.cn/frps/Labiatae  http://frps.eflora.cn/frps/Labiatae  http://frps.eflora.cn/frps/Labiatae  http://frps.eflora.cn/frps/Labiatae  http://frps.eflora.cn/frps/Labiatae  http://frps.eflora.cn/frps/Labiatae  http://frps.eflora.cn/frps/Labiatae  http://frps.eflora.cn/frps/Labiatae  http://frps.eflora.cn/frps/Labiatae  http://frps.eflora.cn/frps/Labiatae  http://frps.eflora.cn/frps/Labiatae  http://frps.eflora.cn/frps/Labiatae  http://frps.eflora.cn/frps/Labiatae  http://frps.eflora.cn/frps/Labiatae  http://frps.eflora.cn/frps/Labiatae  http://frps.eflora.cn/frps/Labiatae  http://frps.eflora.cn/frps/Labiatae  http://frps.eflora.cn/frps/Labiatae  http://frps.eflora.cn/frps/Labiatae  <http://frps.eflora.cn/frps/Labiatae>  http://frps.eflora.cn/frps/Labiatae  http://frps.eflora.cn/frps/Labiatae  http://frps.eflora.cn/frps/Labiatae  http://frps.eflora.cn/frps/Labiatae  http://frps.eflora.cn/frps/Labiatae  http://frps.eflora.cn/frps/Labiatae  http://frps.eflora.cn/frps/Labiatae  http://frps.eflora.cn/frps/Labiatae  http://frps.eflora.cn/frps/Labiatae  http://frps.eflora.cn/frps/Labiatae  http://frps.eflora.cn/frps/Labiatae  http://frps.eflora.cn/frps/Labiatae  http://frps.eflora.cn/frps/Labiatae  http://frps.eflora.cn/frps/Labiatae  http://frps.eflora.cn/frps/Labiatae  http://frps.eflora.cn/frps/Labiatae  http://frps.eflora.cn/frps/Labiatae  http://frps.eflora.cn/frps/Labiatae  http://frps.eflora.cn/frps/Labiatae  http://frps.eflora.cn/frps/Labiatae  http://frps.eflora.cn/frps/Labiatae  http://frps.eflora.cn/frps/Labiatae  http://frps.eflora.cn/frps/Labiatae  http://frps.eflora.cn/frps/Labiatae  http://frps.eflora.cn/frps/Labiatae  http://frps.eflora.cn/frps/Labiatae  http://frps.eflora.cn/frps/Labiatae  http://frps.eflora.cn/frps/Labiatae  http://frps.eflora.cn/frps/Labiatae  http://frps.eflora.cn/frps/Labiatae  http://frps.eflora.cn/frps/Labiatae  http://frps.eflora.cn/frps/Labiatae  http://frps.eflora.cn/frps/Sect.%21Rhiziridium  http://frps.eflora.cn/frps/Sect.%20Rhiziridium  http://frps.eflora.cn/frps/Sect.%23Rhiziridium  http://frps.eflora.cn/frps/Sect.%30Rhiziridium  http://frps.eflora.cn/frps/Sect.%29Rhiziridium  http://frps.eflora.cn/frps/Sect.%24Rhiziridium  http://frps.eflora.cn/frps/Sect.%36Rhiziridium  http://frps.eflora.cn/frps/Sect.%33Rhiziridium  http://frps.eflora.cn/frps/Sect.%31Rhiziridium  http://frps.eflora.cn/frps/Sect.%35Rhiziridium  http://frps.eflora.cn/frps/Sect.%34Rhiziridium  http://frps.eflora.cn/frps/Sect.%28Rhiziridium  http://frps.eflora.cn/frps/Sect.%37Rhiziridium  http://frps.eflora.cn/frps/Sect.%22Rhiziridium  http://frps.eflora.cn/frps/Sect.%25Rhiziridium  http://frps.eflora.cn/frps/Sect.%27Rhiziridium  http://frps.eflora.cn/frps/Sect.%26Rhiziridium  http://frps.eflora.cn/frps/Sect.%32Rhiziridium  http://frps.eflora.cn/frps/Asparagus%20filicinus  http://frps.eflora.cn/frps/Asparagus%20meioclados  http://frps.eflora.cn/frps/Asparagus%20setaceus  http://frps.eflora.cn/frps/Eremurus  http://frps.eflora.cn/frps/Polygonatum%20cirrhifolium  http://frps.eflora.cn/frps/Polygonatum%20verticillatum  http://frps.eflora.cn/frps/Manglietia  http://frps.eflora.cn/frps/Malpighiaceae  http://frps.eflora.cn/frps/Abutilon  http://frps.eflora.cn/frps/Abutilon%20paniculatum  http://frps.eflora.cn/frps/Abutilon  http://frps.eflora.cn/frps/Kydia  http://frps.eflora.cn/frps/Malva  http://frps.eflora.cn/frps/Triumfetta%20cana  http://frps.eflora.cn/frps/Triumfetta  http://frps.eflora.cn/frps/Triumfetta%20rhomboidea  http://frps.eflora.cn/frps/Urena  http://frps.eflora.cn/frps/Sinomenium%20acutum  http://frps.eflora.cn/frps/Stephania  http://frps.eflora.cn/frps/Moraceae  http://frps.eflora.cn/frps/Moraceae  http://frps.eflora.cn/frps/Dipsacus  http://frps.eflora.cn/frps/Dipsacus  http://frps.eflora.cn/frps/Dipsacus  http://frps.eflora.cn/frps/Ardisia  http://frps.eflora.cn/frps/Embelia  http://frps.eflora.cn/frps/Embelia  http://frps.eflora.cn/frps/Myrsine  http://frps.eflora.cn/frps/Myrsine  http://frps.eflora.cn/frps/Nyctaginaceae  http://frps.eflora.cn/frps/Camptotheca  http://frps.eflora.cn/frps/Nyssa%20sinensis  http://frps.eflora.cn/frps/Fraxinus  http://frps.eflora.cn/frps/Fraxinus  http://frps.eflora.cn/frps/Jasminum  http://frps.eflora.cn/frps/Jasminum  http://frps.eflora.cn/frps/Jasminum  http://frps.eflora.cn/frps/Ligustrum  http://frps.eflora.cn/frps/Ligustrum  http://frps.eflora.cn/frps/Ligustrum  http://frps.eflora.cn/frps/Ligustrum  http://frps.eflora.cn/frps/Ligustrum  http://frps.eflora.cn/frps/Ligustrum  http://frps.eflora.cn/frps/Syringa  http://frps.eflora.cn/frps/Phryma%20leptostachya  <http://frps.eflora.cn/frps/Phytolacca%20acinosa>  http://frps.eflora.cn/frps/Phytolacca%20americana  http://frps.eflora.cn/frps/Piper  http://frps.eflora.cn/frps/Piper  http://frps.eflora.cn/frps/Pittosporum%20brevicalyx  http://frps.eflora.cn/frps/Pittosporum%20crispulum  http://frps.eflora.cn/frps/Pittosporum%20heterophyllum  http://frps.eflora.cn/frps/Plantago%20asiatica  http://frps.eflora.cn/frps/Plantago%20cavaleriei  http://frps.eflora.cn/frps/Plantago%20depressa  http://frps.eflora.cn/frps/Plantago%20gentianoides  http://frps.eflora.cn/frps/Plantago%20major  http://frps.eflora.cn/frps/Plumbaginaceae  http://frps.eflora.cn/frps/Gramineae  http://frps.eflora.cn/frps/Gramineae  http://frps.eflora.cn/frps/Gramineae  http://frps.eflora.cn/frps/Gramineae  http://frps.eflora.cn/frps/Gramineae  http://frps.eflora.cn/frps/Gramineae  http://frps.eflora.cn/frps/Gramineae  http://frps.eflora.cn/frps/Gramineae  http://frps.eflora.cn/frps/Gramineae  http://frps.eflora.cn/frps/Gramineae  http://frps.eflora.cn/frps/Gramineae  http://frps.eflora.cn/frps/Gramineae  http://frps.eflora.cn/frps/Gramineae  http://frps.eflora.cn/frps/Gramineae  http://frps.eflora.cn/frps/Gramineae  http://frps.eflora.cn/frps/Gramineae  http://frps.eflora.cn/frps/Gramineae  http://frps.eflora.cn/frps/Gramineae  http://frps.eflora.cn/frps/Gramineae  http://frps.eflora.cn/frps/Gramineae  http://frps.eflora.cn/frps/Gramineae  http://frps.eflora.cn/frps/Gramineae  http://frps.eflora.cn/frps/Gramineae  http://frps.eflora.cn/frps/Gramineae  http://frps.eflora.cn/frps/Gramineae  http://frps.eflora.cn/frps/Gramineae  http://frps.eflora.cn/frps/Gramineae  http://frps.eflora.cn/frps/Gramineae  http://frps.eflora.cn/frps/Gramineae  http://frps.eflora.cn/frps/Gramineae  http://frps.eflora.cn/frps/Gramineae  http://frps.eflora.cn/frps/Gramineae  http://frps.eflora.cn/frps/Gramineae  http://frps.eflora.cn/frps/Gramineae  http://frps.eflora.cn/frps/Gramineae  http://frps.eflora.cn/frps/Gramineae  http://frps.eflora.cn/frps/Gramineae  http://frps.eflora.cn/frps/Gramineae  http://frps.eflora.cn/frps/Gramineae  http://frps.eflora.cn/frps/Gramineae  http://frps.eflora.cn/frps/Gramineae  http://frps.eflora.cn/frps/Gramineae  http://frps.eflora.cn/frps/Gramineae  http://frps.eflora.cn/frps/Gramineae  http://frps.eflora.cn/frps/Gramineae  http://frps.eflora.cn/frps/Gramineae  http://frps.eflora.cn/frps/Gramineae  http://frps.eflora.cn/frps/Gramineae  http://frps.eflora.cn/frps/Gramineae  http://frps.eflora.cn/frps/Gramineae  http://frps.eflora.cn/frps/Gramineae  http://frps.eflora.cn/frps/Gramineae  http://frps.eflora.cn/frps/Gramineae  http://frps.eflora.cn/frps/Gramineae  http://frps.eflora.cn/frps/Gramineae  http://frps.eflora.cn/frps/Gramineae  http://frps.eflora.cn/frps/Gramineae  http://frps.eflora.cn/frps/Gramineae  http://frps.eflora.cn/frps/Gramineae  http://frps.eflora.cn/frps/Gramineae  http://frps.eflora.cn/frps/Gramineae  http://frps.eflora.cn/frps/Gramineae  http://frps.eflora.cn/frps/Antenoron%20filiforme  http://frps.eflora.cn/frps/Polygonaceae  http://frps.eflora.cn/frps/Polygonaceae  http://frps.eflora.cn/frps/Fallopia  http://frps.eflora.cn/frps/Polygonaceae  http://frps.eflora.cn/frps/Polygonaceae  http://frps.eflora.cn/frps/Polygonaceae  http://frps.eflora.cn/frps/Polygonaceae  http://frps.eflora.cn/frps/Polygonaceae  http://frps.eflora.cn/frps/Polygonaceae  http://frps.eflora.cn/frps/Polygonaceae  http://frps.eflora.cn/frps/Polygonaceae  http://frps.eflora.cn/frps/Polygonaceae  http://frps.eflora.cn/frps/Polygonaceae  http://frps.eflora.cn/frps/Polygonaceae  http://frps.eflora.cn/frps/Polygonaceae  http://frps.eflora.cn/frps/Polygonaceae  http://frps.eflora.cn/frps/Polygonaceae  http://frps.eflora.cn/frps/Polygonaceae  http://frps.eflora.cn/frps/Polygonaceae  <http://frps.eflora.cn/frps/Polygonaceae>  http://frps.eflora.cn/frps/Polygonum  http://frps.eflora.cn/frps/Polygonaceae  http://frps.eflora.cn/frps/Polygonaceae  http://frps.eflora.cn/frps/Polygonum  http://frps.eflora.cn/frps/Polygonaceae  http://frps.eflora.cn/frps/Polygonaceae  http://frps.eflora.cn/frps/Polygonaceae  http://frps.eflora.cn/frps/Polygonaceae  http://frps.eflora.cn/frps/Polygonaceae  http://frps.eflora.cn/frps/Polygonaceae  http://frps.eflora.cn/frps/Polygonaceae  http://frps.eflora.cn/frps/Polygonaceae  http://frps.eflora.cn/frps/Polygonaceae  http://frps.eflora.cn/frps/Polygonaceae  http://frps.eflora.cn/frps/Polygonaceae  http://frps.eflora.cn/frps/Polygonaceae  http://frps.eflora.cn/frps/Polygonaceae  http://frps.eflora.cn/frps/Polygonaceae  http://frps.eflora.cn/frps/Polygonaceae  http://frps.eflora.cn/frps/Polygonaceae  http://frps.eflora.cn/frps/Polygonaceae  http://frps.eflora.cn/frps/Polygonaceae  http://frps.eflora.cn/frps/Polygonaceae  http://frps.eflora.cn/frps/Polygonaceae  http://frps.eflora.cn/frps/Polygonaceae  http://frps.eflora.cn/frps/Anemone  http://frps.eflora.cn/frps/Anemone  http://frps.eflora.cn/frps/Anemone  http://frps.eflora.cn/frps/Anemone  http://frps.eflora.cn/frps/Anemone  http://frps.eflora.cn/frps/Anemone  http://frps.eflora.cn/frps/Anemone  http://frps.eflora.cn/frps/Anemone  http://frps.eflora.cn/frps/Anemone  http://frps.eflora.cn/frps/Batrachium  http://frps.eflora.cn/frps/Cimicifuga%20foetida  http://frps.eflora.cn/frps/Cimicifuga%20yunnanensis  http://frps.eflora.cn/frps/Ranunculaceae  http://frps.eflora.cn/frps/Ranunculaceae  http://frps.eflora.cn/frps/Ranunculaceae  http://frps.eflora.cn/frps/Ranunculaceae  http://frps.eflora.cn/frps/Ranunculaceae  http://frps.eflora.cn/frps/Ranunculaceae  http://frps.eflora.cn/frps/Clematis%20lasiandra  http://frps.eflora.cn/frps/Ranunculaceae  http://frps.eflora.cn/frps/Ranunculaceae  http://frps.eflora.cn/frps/Ranunculaceae  http://frps.eflora.cn/frps/Clematis%20parviloba  http://frps.eflora.cn/frps/Ranunculaceae  http://frps.eflora.cn/frps/Ranunculaceae  http://frps.eflora.cn/frps/Clematis%20ranunculoides  http://frps.eflora.cn/frps/Ranunculaceae  http://frps.eflora.cn/frps/Ranunculaceae  http://frps.eflora.cn/frps/Pulsatilla%20millefolium  http://frps.eflora.cn/frps/Ranunculus  http://frps.eflora.cn/frps/Ranunculus  http://frps.eflora.cn/frps/Souliea%20vaginata  http://frps.eflora.cn/frps/Thalictrum  http://frps.eflora.cn/frps/Thalictrum  http://frps.eflora.cn/frps/Thalictrum  http://frps.eflora.cn/frps/Thalictrum  http://frps.eflora.cn/frps/Thalictrum  http://frps.eflora.cn/frps/Thalictrum  http://frps.eflora.cn/frps/Thalictrum  http://frps.eflora.cn/frps/Thalictrum  http://frps.eflora.cn/frps/Thalictrum  http://frps.eflora.cn/frps/Thalictrum  http://frps.eflora.cn/frps/Thalictrum  http://frps.eflora.cn/frps/Thalictrum  http://frps.eflora.cn/frps/Thalictrum  http://frps.eflora.cn/frps/Thalictrum  http://frps.eflora.cn/frps/Thalictrum  http://frps.eflora.cn/frps/Thalictrum  http://frps.eflora.cn/frps/Thalictrum  http://frps.eflora.cn/frps/Thalictrum  http://frps.eflora.cn/frps/Thalictrum  http://frps.eflora.cn/frps/Thalictrum  http://frps.eflora.cn/frps/Berchemia  http://frps.eflora.cn/frps/Berchemia  http://frps.eflora.cn/frps/Hovenia  http://frps.eflora.cn/frps/Hovenia  http://frps.eflora.cn/frps/Rhamnus%20dumetorum  http://frps.eflora.cn/frps/Rhamnus%20gilgiana  http://frps.eflora.cn/frps/Rhamnus%20leptophylla  http://frps.eflora.cn/frps/Rhamnus%20tangutica  http://frps.eflora.cn/frps/Rhamnus%20utilis  http://frps.eflora.cn/frps/Rhamnus%20virgata  http://frps.eflora.cn/frps/Sageretia%20horrida  http://frps.eflora.cn/frps/Ziziphus%20montana  http://frps.eflora.cn/frps/Agrimonia  http://frps.eflora.cn/frps/Agrimonia  <http://frps.eflora.cn/frps/Amygdalus>  http://frps.eflora.cn/frps/Armeniaca  http://frps.eflora.cn/frps/Armeniaca  http://frps.eflora.cn/frps/Cerasus  http://frps.eflora.cn/frps/Cerasus  http://frps.eflora.cn/frps/Cerasus  http://frps.eflora.cn/frps/Cerasus  http://frps.eflora.cn/frps/Cerasus  http://frps.eflora.cn/frps/Coluria  http://frps.eflora.cn/frps/Cotoneaster%20acuminatus  http://frps.eflora.cn/frps/Cotoneaster%20acutifolius  http://frps.eflora.cn/frps/Cotoneaster%20adpressus  http://frps.eflora.cn/frps/Cotoneaster%20buxifolius  http://frps.eflora.cn/frps/Cotoneaster%20coriaceus  http://frps.eflora.cn/frps?id=%E7%9F%AE%E7%94%9F%E6%A0%92%E5%AD%90  http://frps.eflora.cn/frps/Cotoneaster%20dielsianus  http://frps.eflora.cn/frps/Cotoneaster%20divaricatus  http://frps.eflora.cn/frps/Cotoneaster%20foveolatus  http://frps.eflora.cn/frps/Cotoneaster%20franchetii  http://frps.eflora.cn/frps/Cotoneaster%20harrysmithii  http://frps.eflora.cn/frps/Cotoneaster%20hebephyllus  http://frps.eflora.cn/frps/Cotoneaster%20horizontalis  http://frps.eflora.cn/frps/Cotoneaster%20langei  http://frps.eflora.cn/frps/Cotoneaster%20microphyllus  http://frps.eflora.cn/frps/Cotoneaster%20moupinensis  http://frps.eflora.cn/frps/Cotoneaster%20multiflorus  http://frps.eflora.cn/frps/Cotoneaster%20pannosus  http://frps.eflora.cn/frps/Cotoneaster%20rotundifolius  http://frps.eflora.cn/frps/Cotoneaster%20rubens  http://frps.eflora.cn/frps/Cotoneaster%20salicifolius  http://frps.eflora.cn/frps/Cotoneaster%20subadpressus  http://frps.eflora.cn/frps/Cotoneaster%20tenuipes  http://frps.eflora.cn/frps/Crataegus%20chungtienensis  http://frps.eflora.cn/frps/Crataegus%20cuneata  http://frps.eflora.cn/frps/Dichotomanthus  http://frps.eflora.cn/frps/Docynia  http://frps.eflora.cn/frps/Duchesnea  http://frps.eflora.cn/frps/Fragaria  http://frps.eflora.cn/frps/Fragaria  http://frps.eflora.cn/frps/Geum  http://frps.eflora.cn/frps/Geum  http://frps.eflora.cn/frps/Kerria%20japonica  http://frps.eflora.cn/frps/Malus  http://frps.eflora.cn/frps/Malus  http://frps.eflora.cn/frps/Malus  http://frps.eflora.cn/frps/Malus  http://frps.eflora.cn/frps/Malus  http://frps.eflora.cn/frps/Malus  http://frps.eflora.cn/frps/Neillia%20serratisepala  http://frps.eflora.cn/frps/Neillia%20thyrsiflora  http://frps.eflora.cn/frps/Osteomeles%20schwerinae  http://frps.eflora.cn/frps/Padus  http://frps.eflora.cn/frps/Padus  http://frps.eflora.cn/frps/Photinia%20glomerata  http://frps.eflora.cn/frps/Photinia  http://frps.eflora.cn/frps/Photinia%20prunifolia  http://frps.eflora.cn/frps?id=%E7%9F%B3%E6%A5%A0  http://frps.eflora.cn/frps/Potentilla  http://frps.eflora.cn/frps/Potentilla  http://frps.eflora.cn/frps/Potentilla  http://frps.eflora.cn/frps/Potentilla  http://frps.eflora.cn/frps/Potentilla  http://frps.eflora.cn/frps/Potentilla  http://frps.eflora.cn/frps/Potentilla  http://frps.eflora.cn/frps/Potentilla  http://frps.eflora.cn/frps/Potentilla  http://frps.eflora.cn/frps/Potentilla  http://frps.eflora.cn/frps/Potentilla  http://frps.eflora.cn/frps/Potentilla  http://frps.eflora.cn/frps/Potentilla  http://frps.eflora.cn/frps/Potentilla  http://frps.eflora.cn/frps/Prunus  http://frps.eflora.cn/frps/Pyracantha  http://frps.eflora.cn/frps/Pyracantha  http://frps.eflora.cn/frps/Pyracantha  http://frps.eflora.cn/frps/Pyracantha  http://frps.eflora.cn/frps/Pyrus  http://frps.eflora.cn/frps/Pyrus  http://frps.eflora.cn/frps/Pyrus  http://frps.eflora.cn/frps/Rosa  http://frps.eflora.cn/frps/Rosa  http://frps.eflora.cn/frps/Rosa  http://frps.eflora.cn/frps/Rosa  http://frps.eflora.cn/frps/Rosa  http://frps.eflora.cn/frps/Rosa  http://frps.eflora.cn/frps/Rosa  http://frps.eflora.cn/frps/Rosa  http://frps.eflora.cn/frps/Rosa  http://frps.eflora.cn/frps/Rosa  http://frps.eflora.cn/frps/Rosa  http://frps.eflora.cn/frps/Rosa  http://frps.eflora.cn/frps/Rosa  http://frps.eflora.cn/frps/Rosa  http://frps.eflora.cn/frps/Rosa  http://frps.eflora.cn/frps/Rosa  http://frps.eflora.cn/frps/Rosa  http://frps.eflora.cn/frps/Rosa  http://frps.eflora.cn/frps/Rosa  http://frps.eflora.cn/frps/Rosa  <http://frps.eflora.cn/frps/Rosa>  http://frps.eflora.cn/frps/Rosa  http://frps.eflora.cn/frps/Rosa  http://frps.eflora.cn/frps/Rosa  http://frps.eflora.cn/frps/Rosa  http://frps.eflora.cn/frps/Rosa  http://frps.eflora.cn/frps/Rosa  http://frps.eflora.cn/frps/Rubus  http://frps.eflora.cn/frps/Rubus  http://frps.eflora.cn/frps/Rubus  http://frps.eflora.cn/frps/Rubus  http://frps.eflora.cn/frps/Rubus  http://frps.eflora.cn/frps/Rubus  http://frps.eflora.cn/frps/Rubus  http://frps.eflora.cn/frps/Rubus  http://frps.eflora.cn/frps/Rubus  http://frps.eflora.cn/frps/Rubus  http://frps.eflora.cn/frps/Rubus  http://frps.eflora.cn/frps/Rubus  http://frps.eflora.cn/frps/Rubus  http://frps.eflora.cn/frps/Rubus  http://frps.eflora.cn/frps/Rubus  http://frps.eflora.cn/frps/Rubus  http://frps.eflora.cn/frps/Rubus  http://frps.eflora.cn/frps/Rubus  http://frps.eflora.cn/frps/Rubus  http://frps.eflora.cn/frps/Sibbaldia  http://frps.eflora.cn/frps/Sibbaldia  http://frps.eflora.cn/frps/Sorbus%20alnifolia  http://frps.eflora.cn/frps/Sorbus%20coronata  http://frps.eflora.cn/frps?id=%E7%96%A3%E6%9E%9C%E8%8A%B1%E6%A5%B8  http://frps.eflora.cn/frps/Sorbus%20epidendron  http://frps.eflora.cn/frps/Sorbus%20folgneri  http://frps.eflora.cn/frps/Sorbus%20hemsleyi  http://frps.eflora.cn/frps/Sorbus%20hupehensis  http://frps.eflora.cn/frps/Sorbus%20insignis  http://frps.eflora.cn/frps/Sorbus%20koehneana  http://frps.eflora.cn/frps/Sorbus%20oligodonta  http://frps.eflora.cn/frps/Sorbus%20pallescens  http://frps.eflora.cn/frps/Sorbus%20pohuashanensis  http://frps.eflora.cn/frps/Sorbus  http://frps.eflora.cn/frps/Sorbus%20reducta  http://frps.eflora.cn/frps/Sorbus%20rufopilosa  http://frps.eflora.cn/frps/Sorbus%20setschwanensis  http://frps.eflora.cn/frps/Sorbus%20vilmorinii  http://frps.eflora.cn/frps/Sorbus%20wallichii  http://frps.eflora.cn/frps/Sorbus%20wilsoniana  http://frps.eflora.cn/frps/Spenceria%20ramalana  http://frps.eflora.cn/frps/Spiraea  http://frps.eflora.cn/frps/Spiraea  http://frps.eflora.cn/frps/Spiraea  http://frps.eflora.cn/frps/Spiraea  http://frps.eflora.cn/frps/Spiraea  http://frps.eflora.cn/frps/Spiraea  http://frps.eflora.cn/frps/Spiraea  http://frps.eflora.cn/frps/Spiraea  http://frps.eflora.cn/frps/Diplospora  http://frps.eflora.cn/frps/Galium  http://frps.eflora.cn/frps/Galium  http://frps.eflora.cn/frps/Sect.%20Diplophragma  http://frps.eflora.cn/frps/Leptodermis  http://frps.eflora.cn/frps/Rubia  http://frps.eflora.cn/frps/Rubia  http://frps.eflora.cn/frps/Rubia  http://frps.eflora.cn/frps/Boenninghausenia%20albiflora  http://frps.eflora.cn/frps/Skimmia%20arborescens  http://frps.eflora.cn/frps/Zanthoxylum%20acanthopodium  http://frps.eflora.cn/frps/Zanthoxylum%20myriacanthum  http://frps.eflora.cn/frps/Meliosma  http://foc.eflora.cn/content.aspx?TaxonId=10787  http://frps.eflora.cn/frps/Koelreuteria  http://frps.eflora.cn/frps/Sapindus  http://frps.eflora.cn/frps/Tiarella  http://frps.eflora.cn/frps/Lagotis  http://frps.eflora.cn/frps/Ailanthus  http://frps.eflora.cn/frps/Euscaphis  http://frps.eflora.cn/frps/Symplocos%20paniculata  http://frps.eflora.cn/frps/Cryptomeria%20japonica  http://frps.eflora.cn/frps/Tetracentron%20sinense  http://frps.eflora.cn/frps/Anneslea%20fragrans  http://frps.eflora.cn/frps/Sect.%20Eurya  http://frps.eflora.cn/frps/Sect.%21Eurya  http://frps.eflora.cn/frps/Schima  http://frps.eflora.cn/frps/Schima  http://frps.eflora.cn/frps/Ternstroemia%20gymnanthera  http://frps.eflora.cn/frps/Stellera  http://frps.eflora.cn/frps/Trema  http://frps.eflora.cn/frps/Laportea%20bulbifera  http://frps.eflora.cn/frps/Urticaceae  http://frps.eflora.cn/frps/Urtica%20hyperborea  http://frps.eflora.cn/frps/Urtica%20laetevirens  http://frps.eflora.cn/frps/Dipsacus  http://frps.eflora.cn/frps/Callicarpa  http://frps.eflora.cn/frps/Callicarpa  http://frps.eflora.cn/frps/Callicarpa  http://frps.eflora.cn/frps/Caryopteris  http://frps.eflora.cn/frps/Caryopteris  http://frps.eflora.cn/frps/Caryopteris  http://frps.eflora.cn/frps/Clerodendrum  http://frps.eflora.cn/frps/Verbena  http://frps.eflora.cn/frps/Vitex  http://frps.eflora.cn/frps/Ampelopsis%20acutidentata  http://frps.eflora.cn/frps/Cayratia%20japonica  http://frps.eflora.cn/frps/Cayratia%20pedata  http://frps.eflora.cn/frps/Parthenocissus%20semicordata  http://frps.eflora.cn/frps/Tetrastigma%20obtectum  http://frps.eflora.cn/frps/Tetrastigma%20rumicispermum  http://frps.eflora.cn/frps/Tetrastigma%20serrulatum  http://frps.eflora.cn/frps/Tetrastigma%20yunnanense  http://frps.eflora.cn/frps/Vitis |

**TABLE S3** Means, standard deviations (SD) and ranges for seed mass (g), seed number and time to germination (days) at the order level for 1119 species from the Mountains of Southwest China.

| Family | Number of Species | Seed mass (g) | | Seed number | | Time to germination (days) | |
| --- | --- | --- | --- | --- | --- | --- | --- |
|  |  | Mean (SD) | Range | Mean (SD) | Range | Mean (SD) | Range |
| Alismatales | 5 | 13.54 (7.48) | 0.62–19.19 | 2.40 (1.67) | 1–5 | 18.39 (4.34) | 14.00–23.58 |
| Apiales | 83 | 3.59 (3.97) | 0.40–27.08 | 2.90 (4.86) | 2–45 | 36.46 (33.50) | 4.08–247.33 |
| Asparagales | 9 | 14.06 (8.38) | 4.08–34.15 | 85.45 (8.39) | 69–95 | 60.74 (51.70) | 20.25–190.59 |
| Asterales | 194 | 2.37 (2.71) | 0.03–24.19 | 12.37 (74.74) | 1–743 | 12.08 (5.48) | 3.50–45.92 |
| Boraginales | 11 | 3.69 (4.64) | 0.62–17.05 | 4.00 (4) | 4–4 | 17.43 (20.69) | 7.00–75.63 |
| Brassicales | 10 | 3.38 (5.43) | 0.07–14.61 | 25.8 (23.29) | 2–80 | 17.23 (12.17) | 3.50–39.44 |
| Caryophyllales | 66 | 4.25 (4.39) | 0.26–17.61 | 1.32 (1.51) | 1–10 | 14.64 (8.88) | 5.25–47.10 |
| Celastrales | 5 | 15.60 (15.01) | 5.11–41.61 | 6.40 (0.89) | 6–8 | 24.16 (24.55) | 2.68–57.08 |
| Cornales | 6 | 69.81 (100.72) | 14.55–273.93 | 2.17 (0.98) | 1–4 | 32.68 (31.83) | 5.92–91.39 |
| Dioscoreales | 4 | 9.24 (0.51) | 8.65–9.74 | 3.00 (2) | 2–6 | 18.46 (4.47) | 12.86–23.80 |
| Dipsacales | 31 | 10.64 (9.72) | 0.11–31.81 | 4.77 (13.96) | 1–79 | 37.79 (39.06) | 6.87–161.59 |
| Ericales | 23 | 18.55 (30.49) | 0.08–116.65 | 64.13 (153.26) | 1–732 | 24.50 (18.63) | 0.64–63.00 |
| Fabales | 55 | 17.04 (32.05) | 0.47–223.05 | 9.86 (8.55) | 1–40 | 11.43 (2.88) | 7.00–18.09 |
| Fagales | 4 | 0.49 (0.09) | 0.37–0.56 | 1.00 (0) | 1–1 | 13.44 (4.50) | 7.75–18.41 |
| Gentianales | 18 | 3.41 (4.16) | 0.16–13.78 | 93.05 (312.69) | 2–1330 | 17.33 (10.75) | 7.00–48.24 |
| Gnetales | 3 | 18.92 (2.89) | 16.71–22.19 | 2.00 (0) | 2–2 | 19.48 (12.01) | 11.20–33.25 |
| Lamiales | 117 | 5.00 (9.30) | 0.04–57.65 | 10.14 (27.52) | 1–186 | 14.01 (9.32) | 2.33–54.25 |
| Liliales | 25 | 3.94 (4.46) | 0.27–14.01 | 8.32 (12.04) | 2–65 | 20.54 (17.78) | 3.50–60.99 |
| Magnoliales | 3 | 47.59 (36.11) | 15.70–86.79 | 5.00 (4.36) | 2–10 | 11.17 (3.80) | 7.75–15.27 |
| Malpighiales | 13 | 29.24 (46.19) | 0.06–152.29 | 55.85 (178.51) | 1–649 | 15.79 (8.13) | 7.00–38.05 |
| Malvales | 11 | 6.06 (5.06) | 2.29–14.74 | 14.00 (14.35) | 1–45 | 17.73 (6.44) | 7.50–29.07 |
| Pinales | 7 | 9.48 (6.17) | 2.01–21.42 | 176.14 (185.85) | 5–560 | 11.38 (3.53) | 6.26–17.74 |
| Poales | 80 | 2.18 (2.31) | 0.37–12.90 | 1.00 (0) | 1–1 | 16.08 (17.91) | 5.19–162.09 |
| Ranunculales | 87 | 5.64 (5.46) | 0.42–29.60 | 4.38 (12.86) | 1–82 | 24.15 (14.98) | 0.70–97.47 |
| Rosales | 184 | 56.83 (233.73) | 0.05–1787.55 | 3.46 (4.71) | 1–50 | 33.39 (30.69) | 1.00–193.67 |
| Sapindales | 28 | 163.03 (473.30) | 0.24–2145.21 | 2.61 (3.55) | 1–20 | 35.55 (32.46) | 2.52–121.23 |
| Saxifragales | 3 | 0.11 (0.07) | 0.03–0.16 | 302.33 (252.73) | 12–473 | 19.83 (15.78) | 4.67–36.16 |
| Solanales | 8 | 7.14 (7.40) | 0.64–19.32 | 177.00 (295.31) | 1–675 | 14.81 (3.82) | 7.00–18.81 |
| Vitales | 9 | 24.03 (9.24) | 4.26–35.87 | 2.44 (1.13) | 1–4 | 14.88 (8.63) | 4.73–27.30 |
| Zingiberales | 3 | 15.9 (15.28) | 7.01–33.55 | 26.00 (2.65) | 24–29 | 15.33 (2.70) | 12.44–17.79 |

**TABLE S4** Means, standard deviations (SD) and ranges for seed mass (g), seed number and time to germination (days) at the family level for 1119 species from the Mountains of Southwest China.

| Family | Number  of  Species | Seed mass (g) | | Seed number | | Time to germination (days) | |
| --- | --- | --- | --- | --- | --- | --- | --- |
|  |  | Mean (SD) | Range | Mean (SD) | Range | Mean (SD) | Range |
| Aceraceae | 11 | 47.65 (28.06) | 26.59–125.20 | 2.00 (0) | 2–2 | 54.85 (42.02) | 4.66–121.23 |
| Adoxaceae | 13 | 18.85 (9.74) | 1.56–31.80 | 1.46 (1.20) | 1–5 | 58.59 (50.73) | 6.87–161.58 |
| Amaranthaceae | 10 | 1.12 (0.98) | 0.26–2.84 | 1.50 (1.58) | 1–6 | 11.63 (1.85) | 8.09–13.95 |
| Anacardiaceae | 10 | 239.62 (669.80) | 7.63–2145.20 | 1.40 (1.26) | 1–5 | 22.99 (13.14) | 3.95–46.82 |
| Apiaceae | 73 | 3.09 (2.81) | 0.39–15.65 | 2.00 (0) | 2–2 | 34.23 (24.88) | 4.08–116.20 |
| Araceae | 4 | 16.77 (2.24) | 13.92–19.19 | 2.75 (1.70) | 1–5 | 18.51 (5.01) | 14.00–23.57 |
| Araliaceae | 7 | 6.04 (9.43) | 1.33–27.07 | 4.57 (1.13) | 2–5 | 59.13 (84.00) | 4.90–247.33 |
| Asteraceae | 179 | 2.54 (2.74) | 0.56–24.19 | 1.00 (0) | 1–1 | 11.99 (5.46) | 3.50–45.91 |
| Berberidaceae | 30 | 11.30 (4.07) | 5.35–29.60 | 3.00 (1.74) | 1–8 | 26.47 (14.32) | 5.25–77.00 |
| Betulaceae | 4 | 0.48 (0.09) | 0.36–0.56 | 1.00 (0) | 1–1 | 13.44 (4.49) | 7.75–18.41 |
| Bignoniaceae | 4 | 2.71 (1.80) | 0.31–4.68 | 139.25 (36.27) | 103–186 | 12.67 (7.63) | 7.26–23.96 |
| Boraginaceae | 11 | 3.68 (4.63) | 0.62–17.04 | 4.00 (0) | 4–4 | 17.42 (20.68) | 7.00–75.62 |
| Brassicaceae | 10 | 3.38 (5.43) | 0.06–14.60 | 25.80 (23.29) | 2–80 | 17.22 (12.17) | 3.50–39.43 |
| Campanulaceae | 15 | 0.23 (0.13) | 0.03–0.53 | 148.06 (235.97) | 25–743 | 13.05 (5.70) | 7.00–26.62 |
| Caprifoliaceae | 4 | 2.97 (1.43) | 0.93–4.26 | 6.75 (1.50) | 5–8 | 33.80 (26.47) | 8.94–70.85 |
| Caryophyllaceae | 3 | 0.14 (0.06) | 0.10–0.22 | 29.66 (42.73) | 4–79 | 11.49 (4.89) | 7.00–16.71 |
| Celastraceae | 5 | 15.60 (15.01) | 5.10–41.60 | 6.40 (0.89) | 6–8 | 24.16 (24.54) | 2.68–57.08 |
| Chenopodiaceae | 6 | 0.76 (0.43) | 0.37–1.58 | 1.00 (0) | 1–1 | 17.97 (10.88) | 5.25–32.19 |
| Convolvulaceae | 5 | 9.49 (8.60) | 0.63–19.31 | 3.80 (1.78) | 1–6 | 13.99 (4.53) | 7.00–18.36 |
| Cornaceae | 4 | 26.25 (13.92) | 14.55–42.53 | 2.00 (0) | 2–2 | 44.41 (33.62) | 15.75–91.39 |
| Cyperaceae | 18 | 0.74 (0.28) | 0.37–1.29 | 1.00 (0) | 1–1 | 29.84 (33.86) | 5.18–162.09 |
| Dioscoreaceae | 4 | 9.24 (0.50) | 8.65–9.74 | 3.00 (2) | 2–6 | 18.46 (4.47) | 12.85–23.80 |
| Dipsacaceae | 7 | 6.65 (1.63) | 4.19–8.62 | 1.00 (0) | 1–1 | 26.78 (16.59) | 9.06–54.46 |
| Elaeagnaceae | 6 | 12.46 (3.89) | 8.23–18.84 | 1.00 (0) | 1–1 | 17.69 (16.17) | 5.65–50.16 |
| Ephedraceae | 3 | 18.92 (2.88) | 16.71–22.18 | 2.00 (0) | 2–2 | 19.48 (12.01) | 11.19–33.25 |
| Euphorbiaceae | 11 | 24.80 (42.97) | 1.85–152.29 | 3.36 (1.43) | 1–6 | 15.62 (8.88) | 7.00–38.05 |
| Fabaceae | 55 | 17.03 (32.04) | 0.47–223.05 | 9.85 (8.78) | 1–40 | 11.43 (2.88) | 7.00–18.09 |
| Gentianaceae | 5 | 0.68 (0.56) | 0.15–1.53 | 326.60 (567.37) | 4–1330 | 18.64 (16.60) | 9.28–48.24 |
| Geraniaceae | 5 | 3.76 (1.94) | 1.12–6.04 | 5.00 (0) | 5–5 | 13.97 (7.09) | 8.50–25.64 |
| Iridaceae | 9 | 14.06 (8.37) | 4.07–34.15 | 85.45 (8.39) | 69–95 | 60.73 (51.70) | 20.25–190.59 |
| Lamiaceae | 83 | 3.06 (7.41) | 0.03–57.65 | 4.00 (0) | 4–4 | 11.62 (6.73) | 2.33–47.40 |
| Liliaceae | 25 | 3.93 (4.45) | 0.27–14.01 | 8.32 (12.04) | 2–65 | 20.54 (17.77) | 3.50–60.99 |
| Malvaceae | 10 | 6.43 (5.16) | 2.28–14.73 | 15.30 (14.42) | 5–45 | 17.52 (6.75) | 7.50–29.06 |
| Morinaceae | 3 | 8.43 (1.96) | 6.16–9.68 | 1.00 (0) | 1–1 | 13.51 (4.50) | 8.43–16.98 |
| Myrsinaceae | 5 | 47.45 (43.25) | 13.20–116.65 | 1.00 (0) | 1–1 | 30.68 (21.30) | 0.63–60.03 |
| Oleaceae | 12 | 22.16 (10.05) | 7.03–37.86 | 3.66 (0.78) | 2–4 | 26.76 (12.31) | 12.95–47.17 |
| Pinaceae | 5 | 7.28 (3.90) | 2.01–11.25 | 243.20 (179.25) | 116–560 | 11.26 (4.29) | 6.26–17.73 |
| Pittosporaceae | 3 | 9.87 (1.47) | 8.94–11.57 | 21.00 (20.80) | 8–45 | 37.83 (14.18) | 24.51–52.74 |
| Plantaginaceae | 5 | 0.27 (0.02) | 0.23–0.31 | 14.20 (11.21) | 7–34 | 10.11 (2.40) | 7.00–12.56 |
| Poaceae | 62 | 2.60 (2.46) | 0.36–12.89 | 1.00 (0) | 1–1 | 12.08 (4.83) | 5.88–27.31 |
| Polygonaceae | 46 | 5.03 (4.52) | 0.38–17.61 | 1.00 (0) | 1–1 | 14.54 (9.46) | 5.60–47.10 |
| Primulaceae | 7 | 0.21 (0.11) | 0.11–0.45 | 44.26 (21.57) | 28–78 | 28.27 (20.63) | 8.68–63.00 |
| Ranunculaceae | 53 | 2.17 (1.71) | 0.41–7.45 | 5.37 (16.41) | 1–82 | 23.81 (15.23) | 6.60–97.47 |
| Rhamnaceae | 12 | 165.76 (510.70) | 0.70–1787.54 | 2.66 (0.78) | 2–4 | 33.91 (53.38) | 2.00–193.66 |
| Rosaceae | 158 | 52.70 (210.39) | 0.04–1423.77 | 3.74 (5.01) | 1–50 | 34.30 (29.42) | 1.00–145.59 |
| Rubiaceae | 8 | 4.88 (5.64) | 0.33–13.77 | 4.38 (3.77) | 2–12 | 18.59 (9.10) | 7.00–31.03 |
| Rutaceae | 4 | 11.93 (10.95) | 0.24–26.70 | 7.50 (8.35) | 3–20 | 19.41 (24.75) | 2.52–56.00 |
| Saxifragaceae | 3 | 0.10 (0.07) | 0.02–0.15 | 302.33 (252.72) | 12–473 | 19.82 (15.78) | 4.66–36.16 |
| Scrophulariaceae | 3 | 4.98 (5.02) | 0.07–10.10 | 41.33 (61.32) | 2–112 | 18.45 (4.22) | 15.94–23.33 |
| Solanaceae | 3 | 3.21 (2.55) | 0.74–5.85 | 465.66 (324.38) | 92–675 | 16.15 (2.34) | 14.35–18.80 |
| Theaceae | 6 | 11.15 (12.47) | 0.53–27.67 | 28.50 (20.06) | 2–50 | 17.06 (16.94) | 2.80–49.43 |
| Urticaceae | 4 | 12.26 (23.29) | 0.49–47.21 | 1.00 (0) | 1–1 | 26.97 (10.43) | 12.15–36.27 |
| Verbenaceae | 9 | 3.69 (6.62) | 0.30–20.55 | 4.33 (1.41) | 3–8 | 19.97 (13.96) | 6.31–54.25 |
| Vitaceae | 9 | 24.02 (9.23) | 4.26–35.87 | 2.44 (1.13) | 1–4 | 14.87 (8.62) | 4.73–27.30 |
| Zingiberaceae | 3 | 15.90 (15.28) | 7.01–33.54 | 26.00 (2.65) | 24–29 | 15.32 (2.69) | 12.44–17.78 |

**TABLE S5** Ordinary Pearson correlations for seed mass by seed number as well as for seed mass by time to germination at the family level for 1119 species from the Mountains of Southwest China. NA indicates insufficient data to complete the analysis.

| Family | Number  of  species | Traits | | | |
| --- | --- | --- | --- | --- | --- |
|  |  | Seed mass by Seed number | | Seed mass by Time to germination | |
|  |  | Coefficient | *P*-value | Coefficient | *P*-value |
| Aceraceae | 11 | NA | NA | 0.29 | >0.05 |
| Adoxaceae | 13 | -0.90 | <0.001 | 0.56 | 0.04 |
| Amaranthaceae | 10 | -0.47 | >0.05 | 0.19 | >0.05 |
| Anacardiaceae | 10 | 0.86 | 0.001 | -0.32 | >0.05 |
| Apiaceae | 73 | NA | NA | 0.37 | 0.001 |
| Araceae | 4 | -0.07 | >0.05 | -0.27 | >0.05 |
| Araliaceae | 7 | -0.88 | 0.009 | 0.04 | >0.05 |
| Asteraceae | 179 | NA | NA | 0.16 | 0.03 |
| Berberidaceae | 30 | -0.22 | >0.05 | -0.09 | >0.05 |
| Betulaceae | 4 | NA | NA | -0.28 | >0.05 |
| Bignoniaceae | 4 | -0.01 | >0.05 | 0.02 | >0.05 |
| Boraginaceae | 11 | NA | NA | 0.12 | >0.05 |
| Brassicaceae | 10 | -0.84 | <0.001 | 0.06 | >0.05 |
| Campanulaceae | 15 | -0.68 | 0.006 | 0.03 | >0.05 |
| Caprifoliaceae | 4 | 0.15 | >0.05 | 0.36 | >0.05 |
| Caryophyllaceae | 3 | -0.60 | >0.05 | 0.11 | >0.05 |
| Celastraceae | 5 | 0.86 | >0.05 | 0.74 | >0.05 |
| Chenopodiaceae | 6 | NA | NA | 0.01 | >0.05 |
| Convolvulaceae | 5 | -0.25 | >0.05 | -0.45 | >0.05 |
| Cornaceae | 4 | NA | NA | -0.36 | >0.05 |
| Cyperaceae | 18 | NA | NA | 0.47 | 0.04 |
| Dioscoreaceae | 4 | 0.65 | >0.05 | 0.42 | >0.05 |
| Dipsacaceae | 7 | NA | NA | -0.14 | >0.05 |
| Elaeagnaceae | 6 | NA | NA | -0.22 | >0.05 |
| Ephedraceae | 3 | NA | NA | 0.99 | 0.04 |
| Euphorbiaceae | 11 | -0.34 | >0.05 | -0.56 | >0.05 |
| Fabaceae | 58 | 0.19 | >0.05 | 0.04 | >0.05 |
| Gentianaceae | 5 | -0.69 | >0.05 | 0.19 | >0.05 |
| Geraniaceae | 5 | NA | NA | 0.15 | >0.05 |
| Iridaceae | 9 | -0.89 | 0.001 | 0.84 | 0.005 |
| Lamiaceae | 80 | NA | NA | 0.05 | >0.05 |
| Liliaceae | 25 | -0.01 | >0.05 | 0.62 | 0.001 |
| Malvaceae | 10 | -0.54 | >0.05 | 0.28 | >0.05 |
| Morinaceae | 3 | NA | NA | -0.61 | >0.05 |
| Myrsinaceae | 5 | NA | NA | -0.69 | >0.05 |
| Oleaceae | 12 | 0.51 | >0.05 | -0.06 | >0.05 |
| Pinaceae | 5 | 0.22 | >0.05 | 0.19 | >0.05 |
| Pittosporaceae | 3 | -0.62 | >0.05 | 0.86 | >0.05 |
| Plantaginaceae | 5 | -0.09 | >0.05 | 0.13 | >0.05 |
| Poaceae | 62 | NA | NA | -0.09 | >0.05 |
| Polygonaceae | 46 | NA | NA | -0.04 | >0.05 |
| Primulaceae | 7 | -0.55 | >0.05 | -0.64 | >0.05 |
| Ranunculaceae | 53 | -0.12 | >0.05 | 0.10 | >0.05 |
| Rhamnaceae | 12 | -0.31 | >0.05 | 0.75 | 0.004 |
| Rosaceae | 158 | -0.13 | >0.05 | 0.16 | 0.04 |
| Rubiaceae | 8 | -0.39 | >0.05 | 0.83 | 0.01 |
| Rutaceae | 4 | -0.98 | 0.016 | -0.04 | >0.05 |
| Saxifragaceae | 3 | -0.40 | >0.05 | -0.80 | >0.05 |
| Scrophulariaceae | 3 | -0.99 | 0.02 | 0.60 | >0.05 |
| Solanaceae | 3 | -0.16 | >0.05 | -0.01 | >0.05 |
| Theaceae | 6 | -0.93 | 0.008 | -0.31 | >0.05 |
| Urticaceae | 4 | NA | NA | 0.61 | >0.05 |
| Verbenaceae | 9 | 0.17 | >0.05 | -0.02 | >0.05 |
| Vitaceae | 9 | 0.40 | >0.05 | 0.45 | >0.05 |
| Zingiberaceae | 3 | 0.98 | >0.05 | 0.15 | >0.05 |


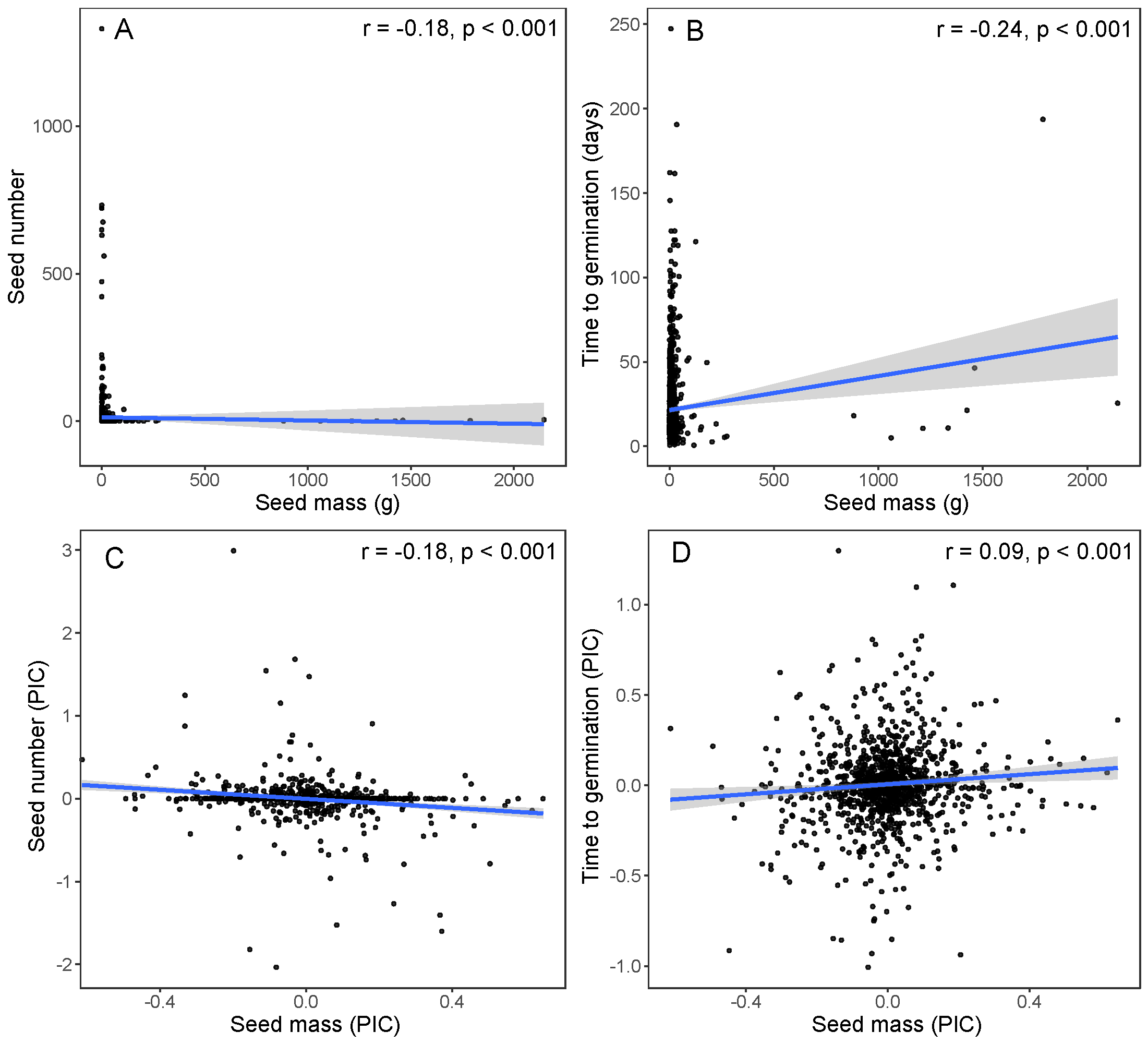


**FIGURE S1** Ordinary Pearson correlations between seed mass and seed number (A) as well as between seed mass and time to germination (B) for 1119 seed plants from the Mountains of Southwest China. (C) and (D) represent Ordinary Pearson correlations with phylogenetically independent contrasts for the same response variables. Raw data shown but analysis based on transformed data. The grey areas represent 95% confidence intervals of models.


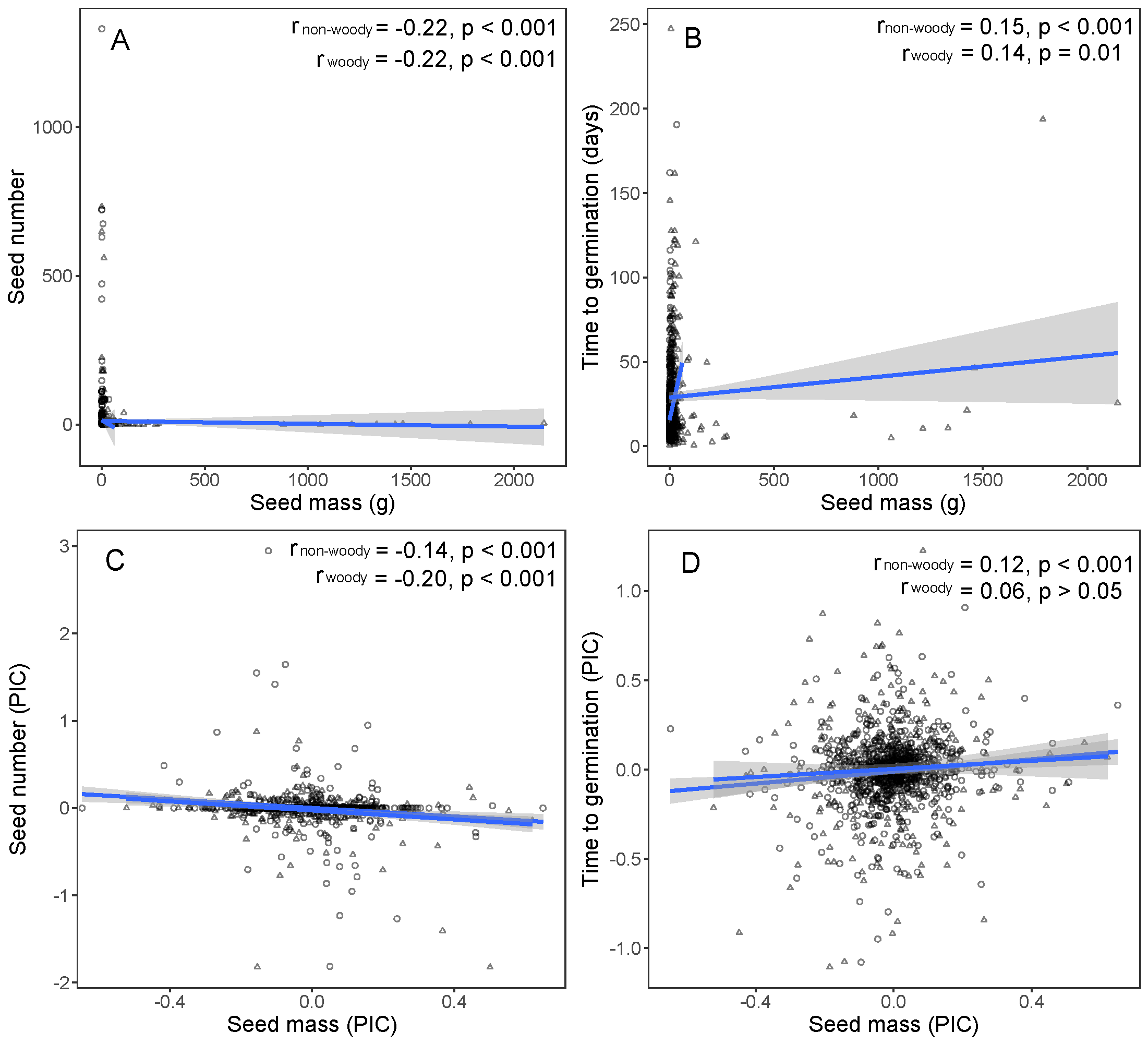


**FIGURE S2** Ordinary Pearson correlations between seed mass and seed number (A) as well as between seed mass and time to germination (B) for 726 non-woody (circles) and 393 woody species (triangles) from the Mountains of Southwest China. (C) and (D) represent Ordinary Pearson correlations with phylogenetically independent contrasts for the same response variables and sources of variation. Raw data shown but analysis based on transformed data. The grey areas represent 95% confidence intervals of models.


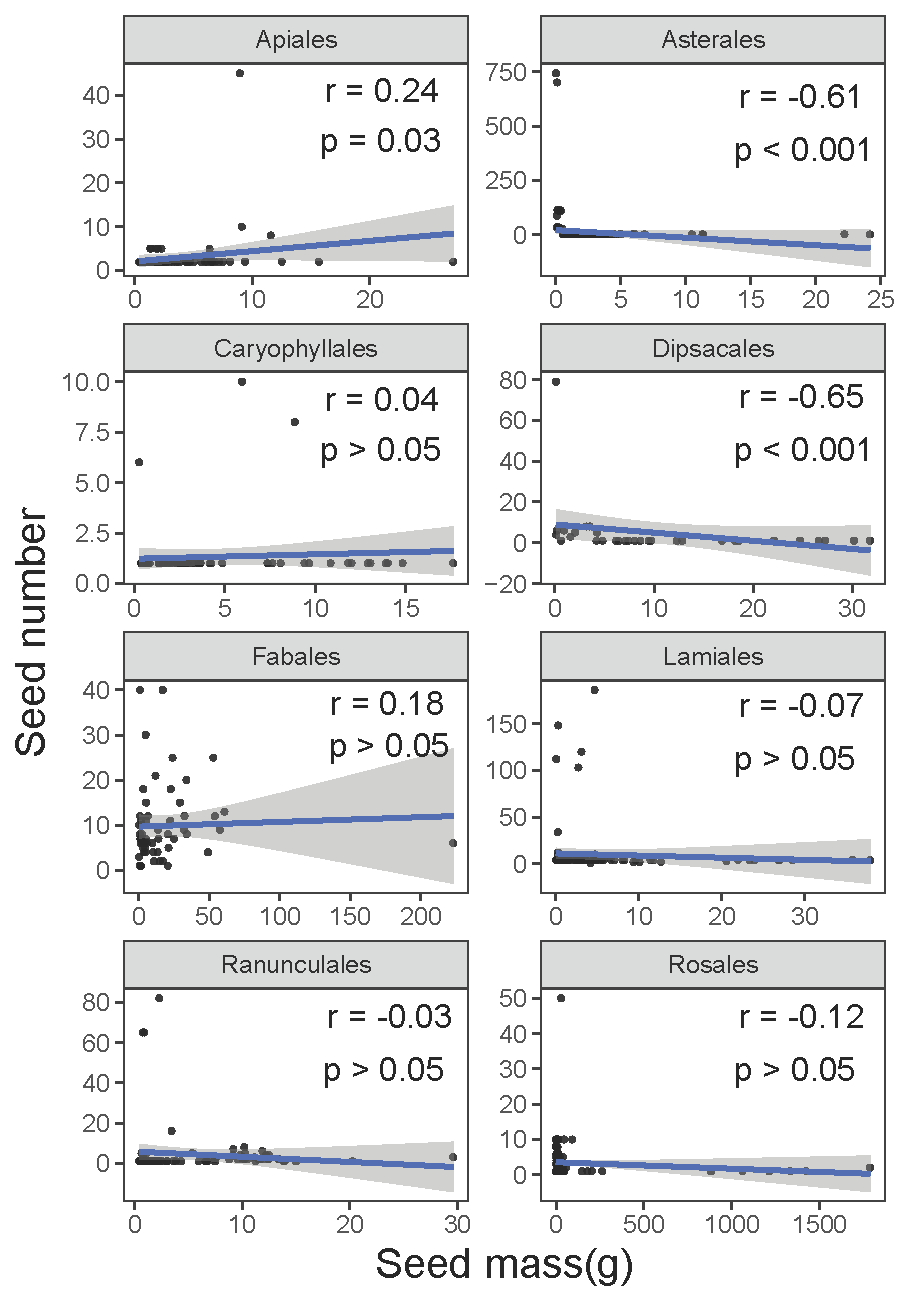


**FIGURE S3** Ordinary Pearson correlations between seed mass and seed number for 1119 seed plants from the Mountains of Southwest China. Shown are Orders in our analysis that have greater than 30 species. All analyses were based on transformed data but each graph depicts raw values for each Order. The grey areas represent 95% confidence intervals of models.


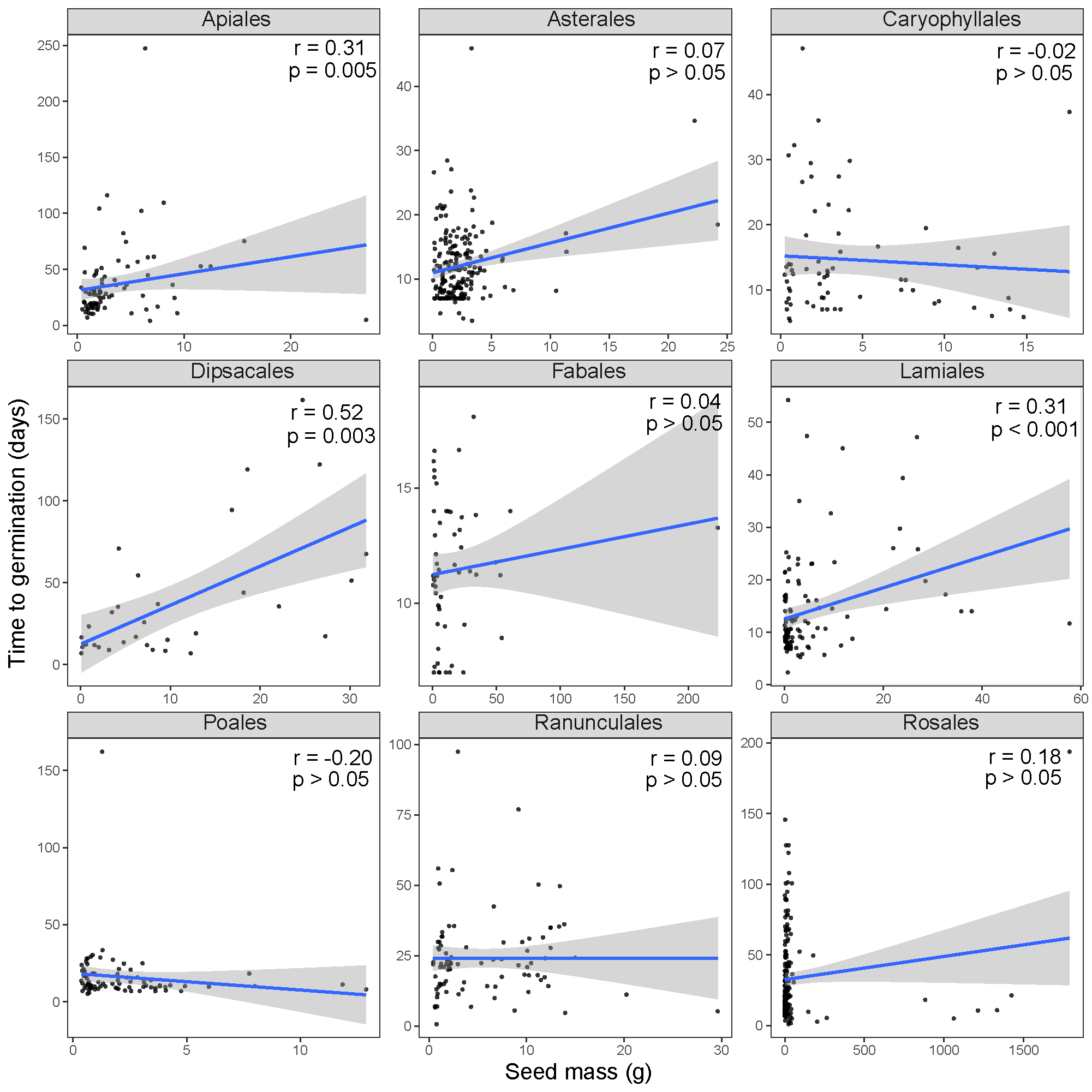


**FIGURE S4** Ordinary Pearson correlations between seed mass and time to germination for 1119 seed plants from the Mountains of Southwest China. Shown are Orders in our analysis that have greater than 30 species. All analyses were based on transformed data but each graph depicts raw values for each Order. The grey areas represent 95% confidence intervals of models.


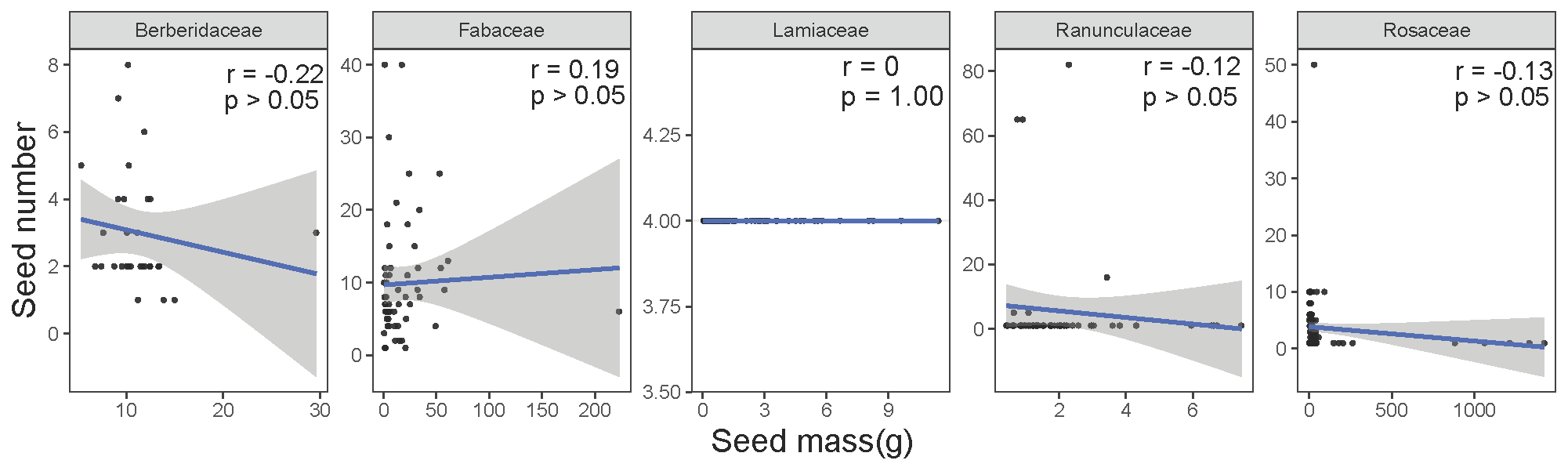


**FIGURE S5** Ordinary Pearson correlations between seed mass and seed number for 1119 seed plants from the Mountains of Southwest China. Shown are Families in our analysis that have greater than 30 species. All analyses were based on transformed data but each graph depicts raw values for each Family. The grey areas represent 95% confidence intervals of models.


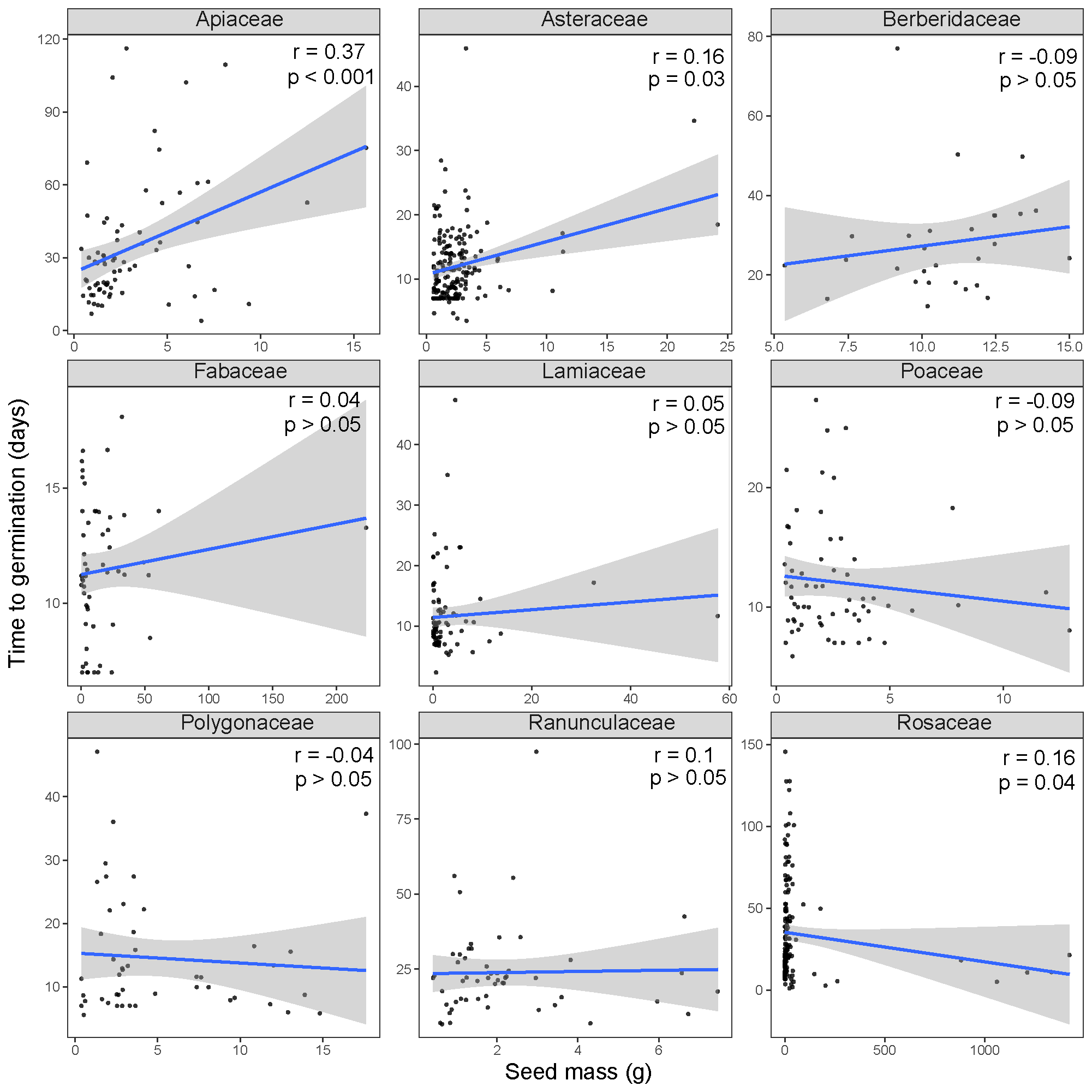


**FIGURE S6** Ordinary Pearson correlations between seed mass and time to germination for 1119 seed plants from the Mountains of Southwest China. Shown are Families in our analysis that have greater than 30 species. All analyses were based on transformed data but each graph depicts raw values for each Families. The grey areas represent 95% confidence intervals of models.


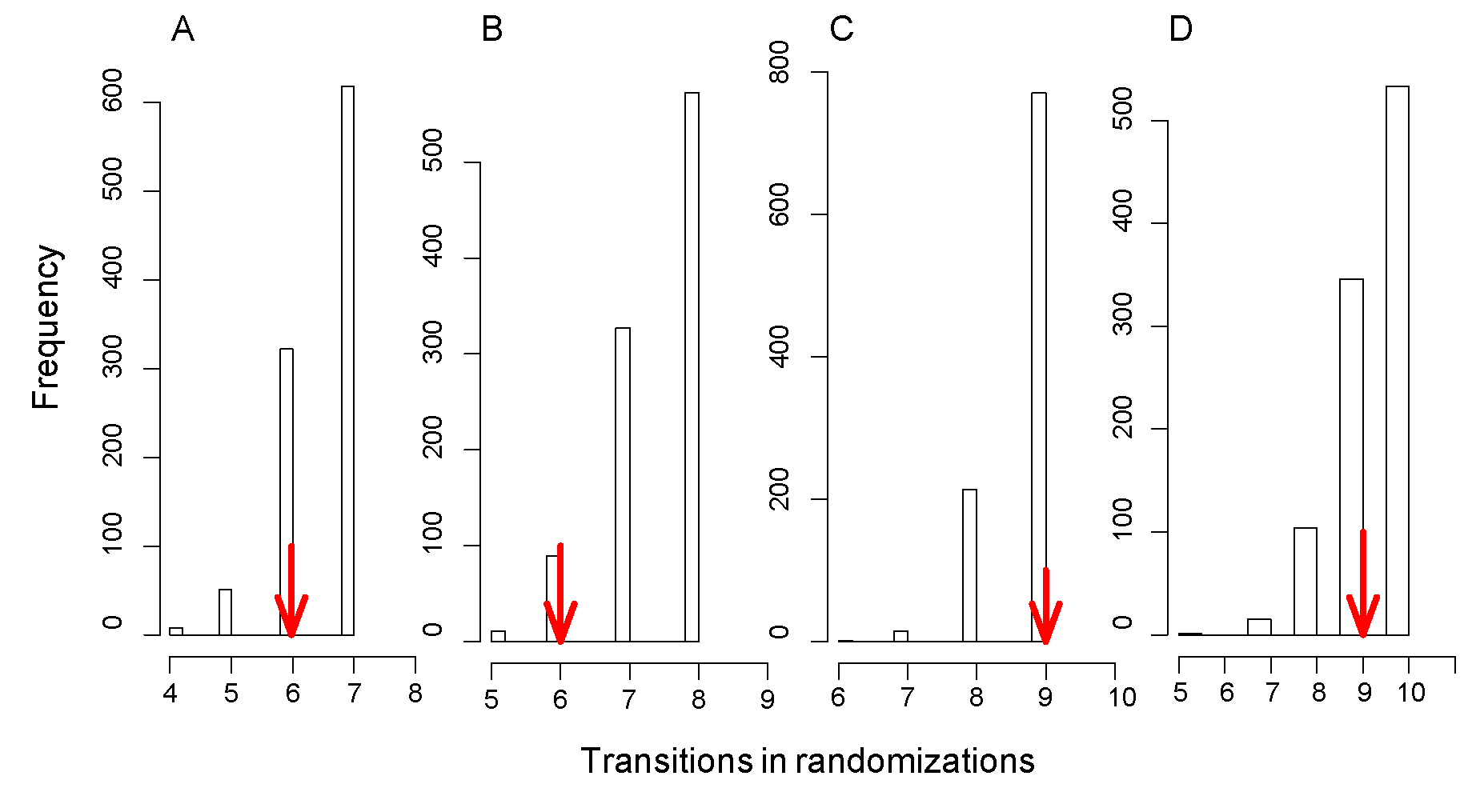


**FIGURE S7** Distributions of numbers of evolutionary transitions under the null model in which data were reshuffled 1000 times across the tips of the phylogeny and the number of observed evolutionary transitions (red arrows) at the order (A,B) and family (C,D) levels. Graphs illustrate the phylogenetic signal for the negative, positive and lack of relation between seed mass and seed number (A,C) as well as the negative, positive and lack of relation between seed mass and time to germination (B,D).


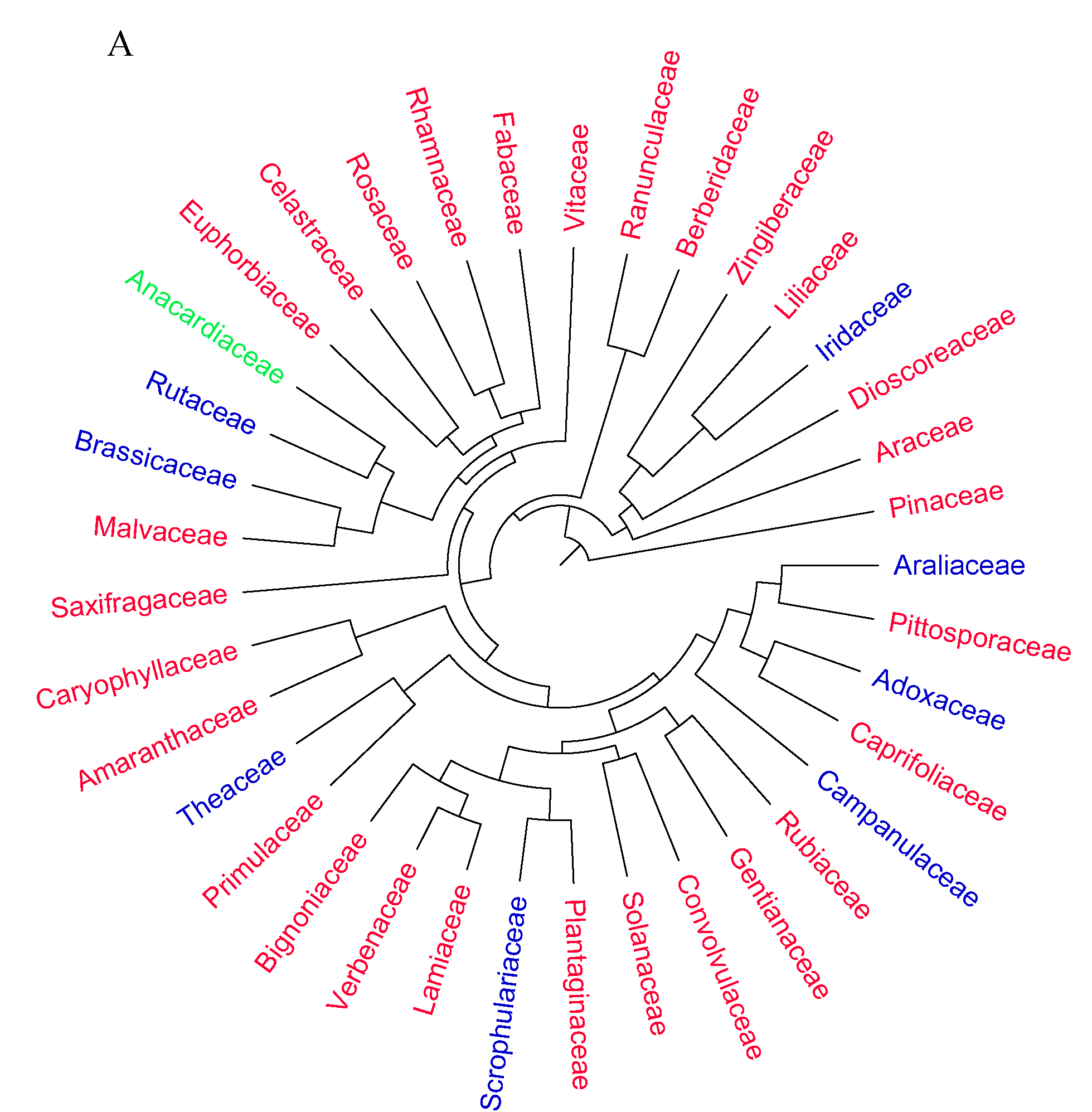


Fig. S8 A


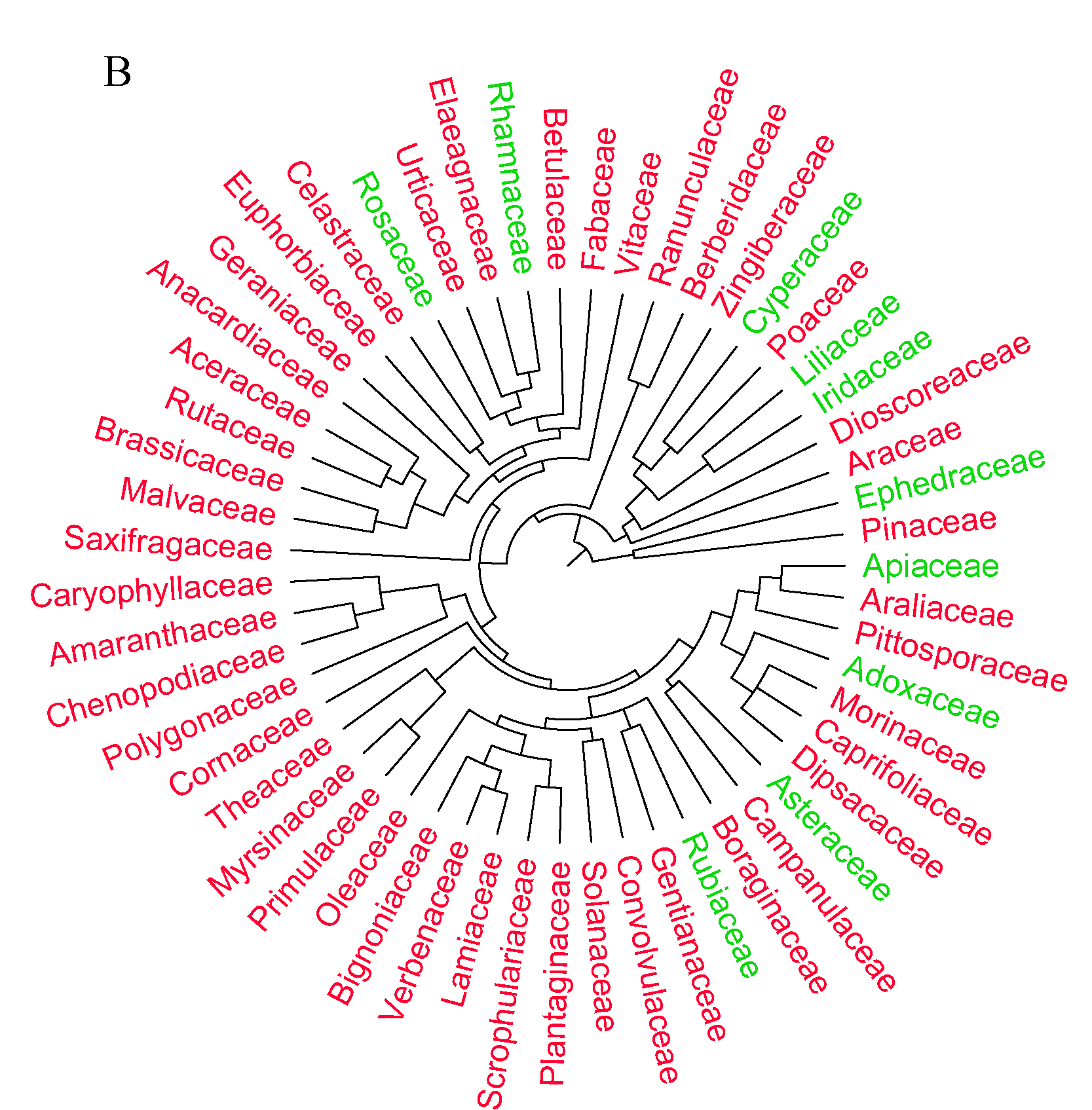


Fig. S8 B

**FIGURE S8** Phylogenetic tree based on APGIII at the family level for 1119 samples collected from our study site in the Mountains of Southwest China. Tree depicts the phylogenetic dispersion of the relation between (A) seed mass and seed number as well as (B) seed mass and time to germination. The taxa in BLUE indicate the presence of a significant negative relation while the taxa highlighted GREEN indicate the occurrence of a significant positive relation. Taxa highlighted in RED did not show a significant relation among seed traits.
